# Photoreversible defect complexation dynamically modulates guest adsorption in metal-organic frameworks

**DOI:** 10.1038/s41467-026-78352-9

**Published:** 2026-10-15

**Authors:** Qi Huang, Simon Astley, Nikita Rog, Boon Kee Voon, Tushar Singh Verma, Wenyu Shen, Ruixue Sun, Wai Fen Yong, Ravichandar Babarao, Yi Huang, Xianfeng Fan, Cher Hon Lau

**Affiliations:** 1 School of Engineering, The University of Edinburgh, Edinburgh, UK; 2 Department of Physics, Aberystwyth University, Aberystwyth, UK; 3 Centre for Advanced Materials & Industrial Chemistry (CAMIC) and ARC Centre of Excellence for Electrochemical Transformation of Carbon Dioxide, School of Science, RMIT University, Melbourne, VIC Australia; 4 Commonwealth Scientific and Industrial Research Organisation (CSIRO), Clayton, VIC Australia; 5 Academy of Scientific and Innovative Research (AcSIR), Ghaziabad, India; 6 Physical Chemistry Division, CSIR-National Chemical Laboratory, Pune, India; 7 Centre of Excellence for Green and Advanced Technologies in Efficient Separations (GATES), School of Energy and Chemical Engineering, Xiamen University Malaysia, Sepang, Malaysia; 8 State Key Laboratory of Physical Chemistry of Solid Surfaces, College of Chemistry and Chemical Engineering, Xiamen University, Xiamen, China

**Keywords:** Metal-organic frameworks, Chemical engineering

## Abstract

Reversible light-driven adsorption in framework materials offers a route to energy-efficient separations, yet current mechanisms deliver modest working capacities that constrain practical utility. Here we introduce photo-reversible defect complexation as a design principle for enhancing light-induced working capacities, coupling photoswitch isomerisation to active binding site modulation rather than pore geometry changes alone. We exploit coordinatively unsaturated Zr sites in defective UiO-67 as reversible binding sites, where a grafted spiropyran (SP) photoswitch undergoes UV-induced isomerisation to the zwitterionic merocyanine (MC) form, whose phenolate oxygen coordinates an additional open Zr site, suppressing guest binding availability. Visible light fully reverses this process. This mechanism delivers an 83% enhancement in photo-modulated CO_2_ working capacity, reaching 1.1 mmol g^−1^, nearly double that of the structural analog of P-UiO that does not enable photo-reversible MC-defect switching. Light-controlled metal cation and CO_2_ uptake confirms that binding site availability, rather than pore geometry, drives this switching response. Density functional theory (DFT) simulations and multimodal X-ray photoelectron spectroscopy (XPS) confirm reversible MC-Zr site complexation as the operative mechanism with performance retained across multiple photocycles. These findings establish photo-reversible defect engineering as a robust and broadly applicable principle for light-responsive frameworks with modulable guest adsorption.

## Abstract (web summary)

Here the authors describe a metal-organic framework in which UV light blocks metal binding sites and visible light reopens them, raising usable carbon dioxide capacity by 83 % and enabling gas capture driven by light rather than heat or pressure.

## Introduction

Biological porous systems dynamically regulate pore structures and functions in response to external stimuli^1–3^, yet achieving comparable remote and precise control in synthetic porous materials remains a fundamental challenge^4–6^. Metal-organic frameworks (MOFs) have emerged as a leading platform for stimuli-responsive materials owing to their high specific surface areas, tunable pore architectures, and chemically addressable active adsorption sites (e.g., open metal sites and functional groups)^7,8^. Exploiting these properties, photo-responsive MOFs have been developed for sustainable metal separation^9–11^, light-induced gas separation^12–14^, and on-demand release of dyes^15^ and uranium^16^. These applications rely on light-driven swing adsorption (LISA), in which photoisomerization remotely modulates pore size^17,18^, polarity^19^, or guest binding energy^20–22^ through the incorporation of photo-switchable units into MOF backbones, side chains, or pore environments^23–28^. Despite significant progress in photo-modulating pore structure, polarity, and host-guest interactions, photo-controlled modulation of active adsorption sites, the primary mechanism underpinning uptake capacity at low pressures remains underdeveloped.

This gap is significant. Under low-pressure conditions, open metal sites in MOFs act as high-affinity binding sites that dominate adsorption^20^, yet this same affinity causes incomplete desorption and reduces working capacities over repeated cycles^29^. Grafting photoswitchable units directly onto active adsorption sites would offer reversible on-off control of binding energy, reducing regeneration energy and improving cycling durability. However, no strategy for achieving this has been established.

Spiropyrans (SPs) are amongst the most widely studied molecular photoswitches, undergoing ultrafast and reversible photoisomerization between a neutral closed form and a zwitterionic merocyanine (MC) form^10,11,30^. Critically, the negatively charged phenolate oxygen in the MC form is known to complex with metal ions in solution^31,32^, suggesting that MC moieties incorporated into defective MOFs could similarly complex with open metal sites. This will effectively enable light-driven, reversible modulation of defect chemistry. When appropriately designed, this photo-reversible MC-open metal site complexation would provide a fundamentally LISA mechanism governed by reversible switching of active adsorption site availability rather than steric pore-size changes or polarity shifts^33–35^. Despite recent progress incorporating SP into defective MOFs to achieve ultrafast switching^36^, the deliberate exploitation of MC-open metal site complexation to modulate defect engineering and guest uptake has received limited attention.

**Fig. 1:**
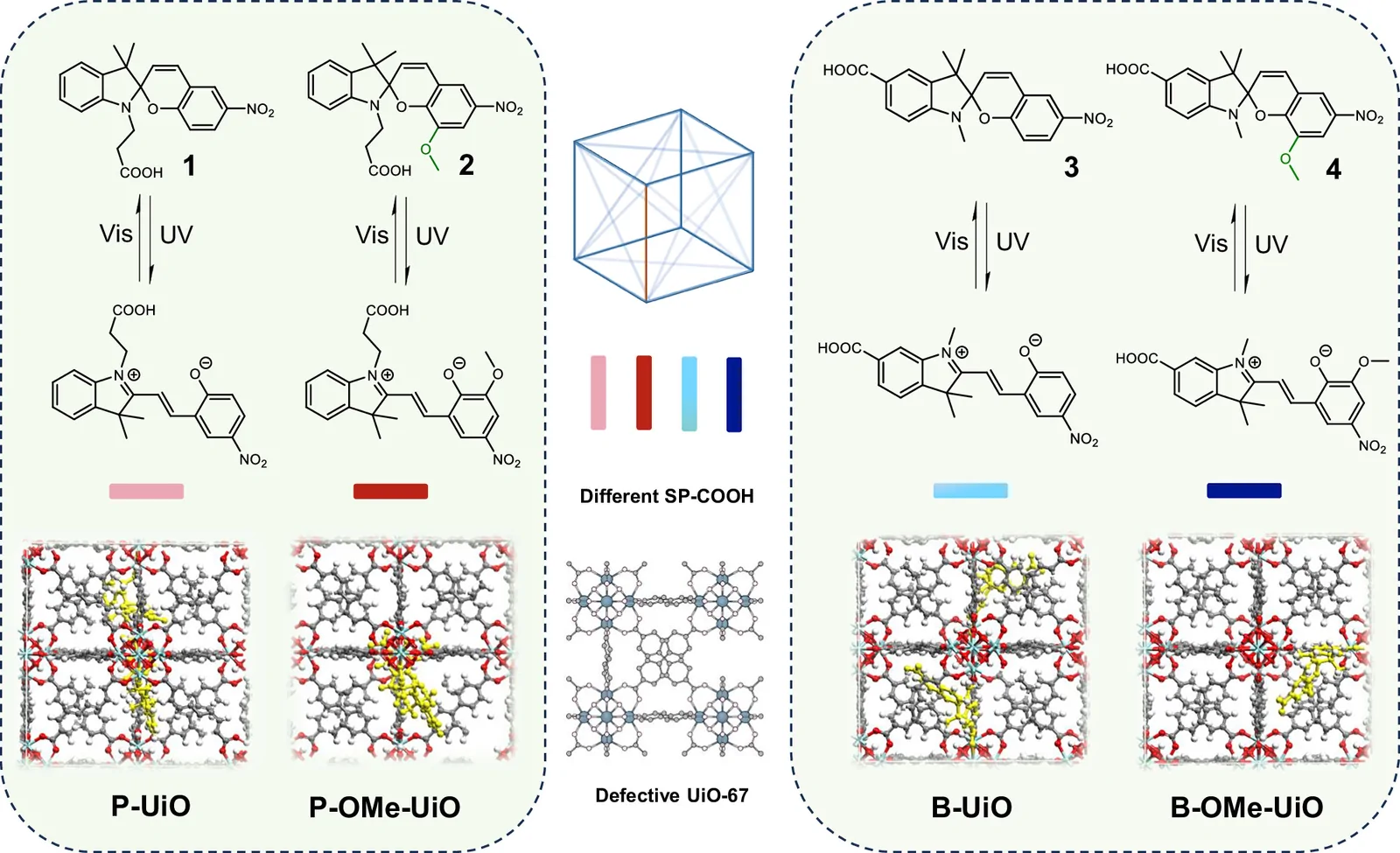
Chemical structures of mono-carboxylated spiropyran (SP) ligands 1- 4 with carboxylic acid groups located at the indoline pyrrole ring (1: P-UiO) or benzene ring (3: B-UiO), with a methoxy (OMe) substituent (2: P-OMe-UiO, 4: B-OMe-UiO). Simulated structures of a unit cell of UiO-67 grafted with 1- 4 show that there is only one SP unit in each of P-OMe-UiO and B-OMe-UiO, and two SP units in each of P-UiO and B-UiO. Atoms in the molecular structures are color-coded as follows: C, gray; H, white; N, blue; Zr, cyan; and O, red.

Here, we investigate this mechanism and establish photo-reversible defect complexation as a distinct and designable route to light-controlled guest adsorption in MOFs. We synthesize and graft a series of mono-carboxylated SP ligands, **1**–**4**, onto the open metal sites of defective UiO-67 (Fig. 1). In our approach, we exploit the carboxylic acid group to graft the ligand onto the partially open Zr node, leaving the SP unit free to photo-isomerize into its zwitterionic MC form^37^. The phenolate oxygen in MC reversibly coordinates with open Zr nodes, reducing CO_2_ affinity and binding energy under UV irradiation and restoring them under visible light. Ex situ, in situ, near-ambient pressure (NAP) X-ray photoelectron spectroscopy (XPS) and gas adsorption analyses show that the presence of a methoxy group next to the phenyl oxygen in SP is key for promoting MC complexation with open Zr sites. At 273 K, the CO_2_ working capacities of MOFs displaying photo-reversible MC-Zr complexation reach 1.1 mmol g^−1^, 83% higher than the structurally analogous P-UiO. Experimental correlation analysis confirms that MC-Zr complexation underpins this enhancement, with the methoxy substituent in P-OMe-UiO identified as the critical structural feature that activates it. Density functional theory (DFT) simulations attribute this enhancement to stronger CO_2_ binding energies. These findings, alongside a life-cycle assessment (LCA), show that photo-reversible defect modulation of MOFs provides an efficient and low-energy strategy for CO_2_ capture and for designing next-generation light-driven sorbents.

## Results and discussion

### Structural characterization of SP-UiOs

**Fig. 2:**
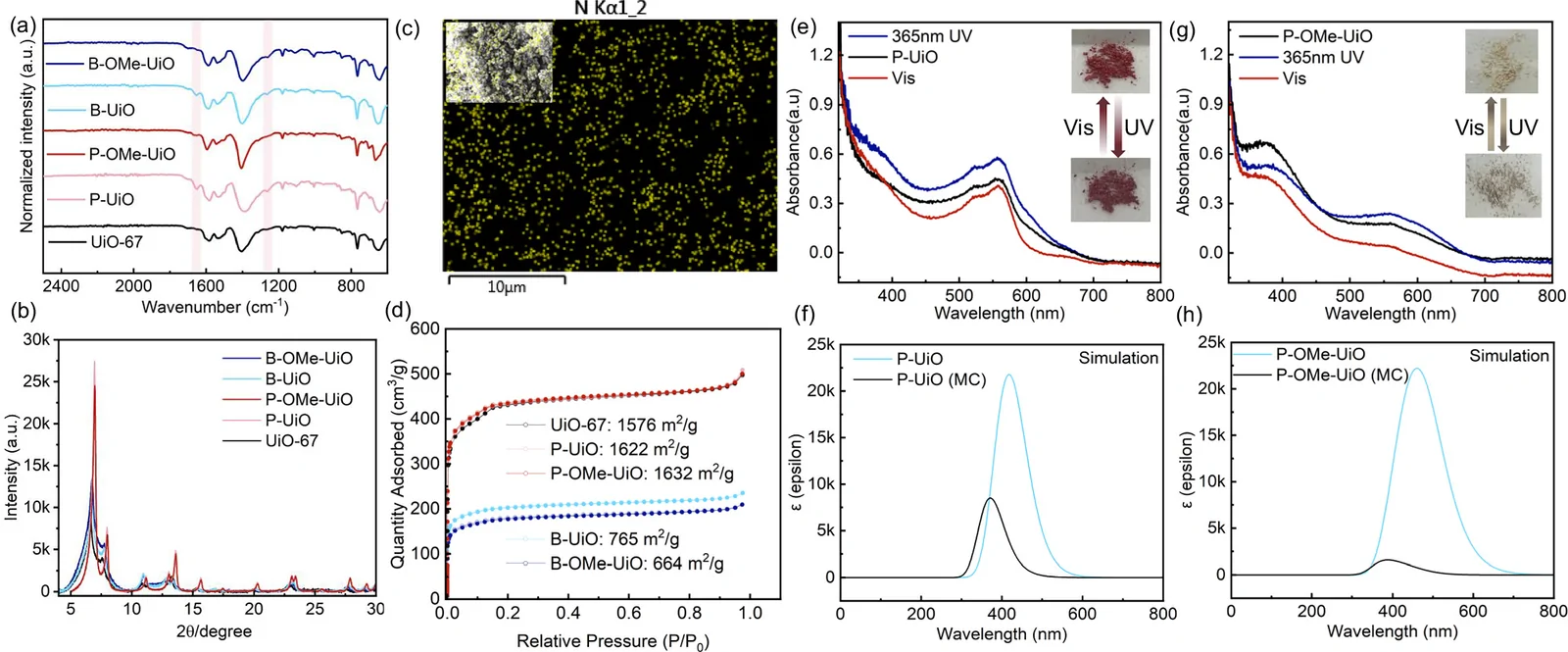
Characterization of spiropyran (SP)-functionalized UiO-67. **a** Fourier-transform infrared spectroscopy (FTIR) spectra confirming SP incorporation via characteristic non-aromatic C=C stretching (1680 cm^−1^) and C–O/C–N stretching (1250 cm^−1^) vibrations (Shaded regions). **b** powder X-ray diffraction (PXRD) patterns of SP-UiO and defective UiO-67 indicated the successful formation of SP-UiOs Frameworks. **c** energy-dispersive X-ray spectroscopy (EDX) N mapping of P-OMe-UiO suggested a uniform distribution of **2** across samples. **d** N_2_ adsorption (●) – desorption (○) isotherms of activated SP-UiO metal-organic frameworks (MOFs) at 77 K showing that pyrrole-COOH ligands enhanced Brunauer–Emmett–Teller (BET) surface area by 3% while benzene-COOH ligands reduced BET surface area by ∼54 %. Photo-switching of **e** P-UiO and **g** P-OMe-UiO is observed with ultraviolet-visible (UV–Vis) diffuse reflectance spectroscopy (DRS) before (365 nm UV) and after (450 nm visible light) irradiation (top) and validated with **f**, **h** time-dependent density functional theory (TD-DFT) calculations that match SP and merocyanine (MC) states.

Following the protocol of Thaggard et al.^36^, we grafted **1**–**4** on to the open Zr nodes of defective UiO-67 to synthesize a series of SP-UiOs. The carboxylic acid group in **1** and **2** is located on the indoline pyrrole (P) and on the indoline benzene (B) for **3** and **4** (Fig. S7). Meanwhile the difference between **1,** **3** and **2,** **4** is the presence of a methoxy (OMe) group at the nitrobenzopyran unit of the latter ligands. As such, we named these MOFs as P-UiO, P-OMe-UiO, B-UiO, and B-OMe-UiO. Based on ¹H nuclear magnetic resonance (NMR; Figs. S8–11) and thermogravimetric analysis (TGA; Fig. S6), there were 12.7, 7.3, 12.4, and 10.2 wt% of SP in P-UiO, P-OMe-UiO, B-UiO, and B-OMe-UiO, respectively. The presence of SP in these MOFs was also validated with Fourier-transform infrared (FTIR) analyses (Fig. S5) where we observed peaks characteristic of non-aromatic C=C stretching vibration and C-O/C-N stretching of SP at approximately 1680 cm^−1^ and 1250 cm^−1^ (Fig. 2a).

The intensities of these peaks in the FTIR spectra of UiO-67 comprising **2** (P-OMe-UiO) and **4** (B-OMe-UiO) were lower, possibly due to the bulky methoxy group limiting SP accessibility into MOF pore cavities, thereby lowering SP content. Powder X-ray Diffraction (PXRD) demonstrated the successful formation of SP-UiOs frameworks. Besides, the position of the carboxylic acid group also impacted MOF ordered range (Fig. 2b). The P-series materials exhibit four times smaller full width at half maximum (FWHM) of the primary diffraction peak at 2*θ* = 6.6° than the B-series, corresponding to four times larger crystallite sizes by Scherrer analysis (Table S1). This directly demonstrates that alkyl chain connectivity promotes significantly more ordered framework assembly than the rigid benzene-ring-tethered carboxylic acid in ligands 3 and 4. The alkyl chains in ligands 1 and 2 could reduce steric hindrance and enable grafting onto defect sites in the UiO-67^38^, enhancing the framework ordering of resultant MOFs.

The different SP species grafted in the UiO-67 framework also impacted MOF porosity. The Brunauer–Emmett–Teller (BET) surface areas of P-UiO and P-OMe-UiO were ~3% higher than those of parent defective UiO-67 (Fig. 2d), reaching up to 1622 and 1632 m²g⁻¹, respectively. Grafting 1 and 2 onto missing-linker sites in defective UiO-67 thus slightly enhances rather than reduces porosity. Their carboxylic acid groups can displace residual modulator species at open Zr sites, reopening previously occluded pore channels^39–41^. Meanwhile, coordination to the open Zr nodes restores partial framework connectivity and reduces local disorder. These are evidenced by the narrower PXRD peak widths of P-UiO and P-OMe-UiO relative to the parent framework. In contrast, with the carboxylic acid group located on the indoline benzene ring, the BET surface areas and pore volumes of B-UiO and B-OMe-UiO were significantly reduced by >50% (Table S2). This was most likely due to steric hindrance caused by the benzene ring, leading to SP units filling up MOF pore cavities and reducing the amount of accessible pore volume. Meanwhile, the pore sizes of SP-UiO MOFs developed here were similar to those in the parent defective UiO-67 (Fig. S12).

Scanning electron microscopy (SEM) combined with energy-dispersive X-ray spectroscopy (EDX) revealed that grafting **1**–**4** into UiO-67 did not affect MOF particle morphology (Fig. S13). EDX N mapping revealed a relatively uniform distribution of nitrogen across these samples (Figs. 2c and S14), indicating homogeneous incorporation of **1**–**4** within the MOF matrix.

TGA analyses showed that **1**–**4** reduced the thermal stability of defective UiO-67. This could be attributed to the lower decomposition temperature of **1**–**4**, relative to the H_2_BPDC ligand (Fig. S6d). Based on our analyses, we concluded that SP was uniformly grafted onto the defects of UiO-67 and the morphologies of SP-UiO MOFs were maintained regardless of SP content and type. Optimized cluster models showed that there were only 1–2 SP molecule(s) per unit cell of SP-UiOs.

### Photo-reversible properties of SP-UiO-67 MOFs

The uniform distribution of SP in SP-UiO MOFs was key to fully reversible, distinct color changes in the solid state upon exposure to UV (*λ* = 365 nm) and visible (*λ* = 400–780 nm) light (Figs. 2e, g and S16) from off-the-shelf torches. For example, the color of P-OMe-UiO (red) darkened immediately upon UV irradiation, and this color change was reversed when the MOF was in darkness or within 5 min of visible light irradiation. In addition, P-UiO, B-UiO, and B-OMe-UiO also suggested distinct color changes in the solid state. Time-dependent density functional theory (TD-DFT) calculations validated that these color changes were due to switching between the SP and MC states in the MOF. These fast color changes between SP and MC states could be due to the low concentration and spatial distribution of SP that prevented close packing, minimizing π-π stacking or hydrogen-bonding interactions that impede photo-isomerization in the solid state^30^.

**Fig. 3:**
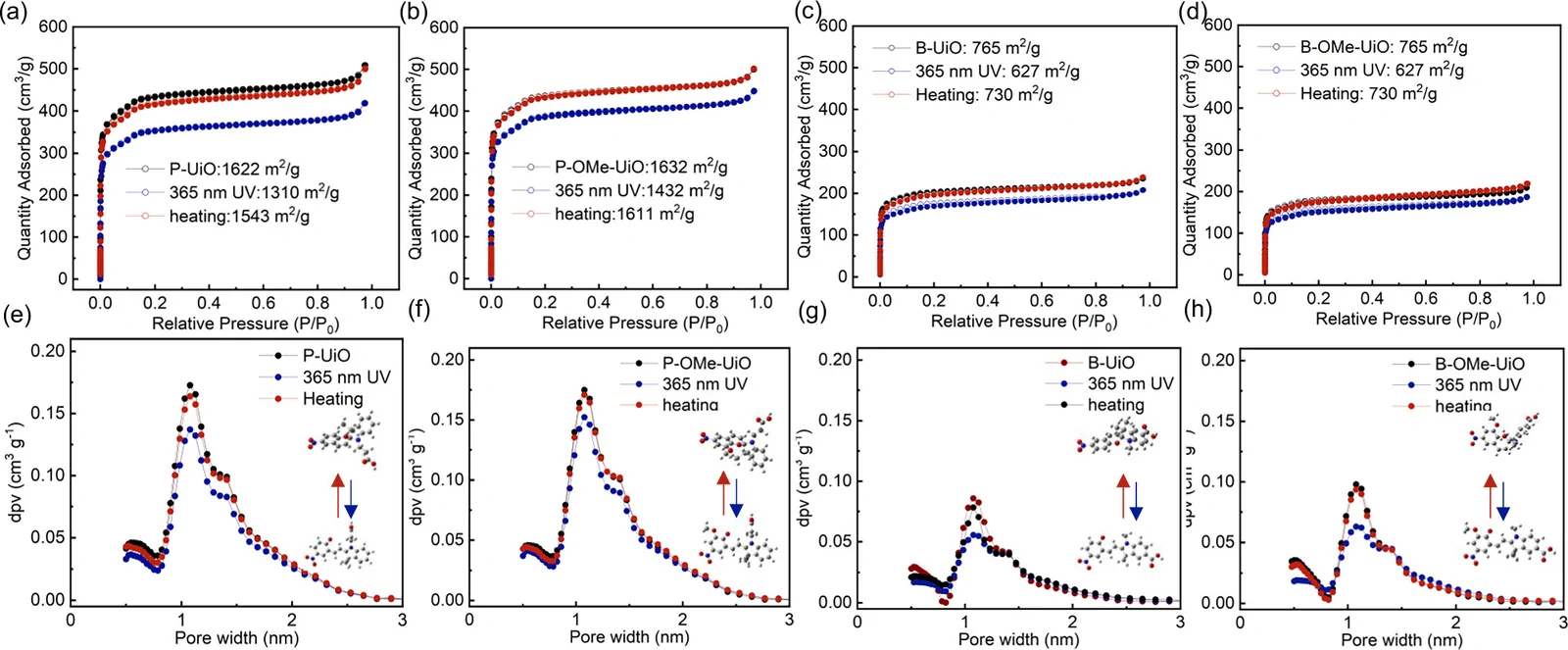
Photo-responsive conversion of porous structures. **a**–**d** N_2_ adsorption (●) – desorption (○) isotherms and **e**–**h** pore size distributions of P-UiO, P-OMe-UiO, B-UiO, and B-OMe-UiO in the pristine state (black), after photo-irradiation of a sealed Brunauer–Emmett–Teller (BET) cell with ultraviolet (UV) light from a light source for 1 h (blue), and lateral thermal regeneration at 100 °C (red). Atoms in the molecular structures are color-coded as follows: C, gray; H, white; N, blue; and O, red.

In addition to photo-reversible color changes, the porous structures of SP-UiO MOFs studied here could also be photo-modulated (Fig. 3). The BET surface areas and total pore volumes of UV-irradiated SP-UiOs were lower than samples that were not exposed to UV light. Upon UV irradiation, the BET surface areas of P-UiO and P-OMe-UiO were reduced by 19% and 12%, from 1622 and 1632 m^2^g^−^^1^ to 1310 and 1432 m^2^g^−^^1^, respectively (Fig. 3a, b). The total pore volumes of these MOFs were reduced by 18% and 11% (Table S2), while their pore sizes (centered at 0.5 and 1.2 nm) remained intact during photo-isomerization. The UV-induced pore volume changes in SP-UiOs studied here were fully reversible upon lateral thermal heating. These photo-modulated reductions in BET surface areas and accessible pore volumes were attributed to the photo-isomerization of SP (8 Å) to the open, extended MC form (12 Å), which likely occupied more pore space or restricted gas diffusion within the MOF framework^30^. These photo-modulated changes in SP-UiOs pore volumes were validated with calculations based on DFT-optimized crystal structures of the lowest-energy SP and MC configurations (Fig. S18).

**Fig. 4:**
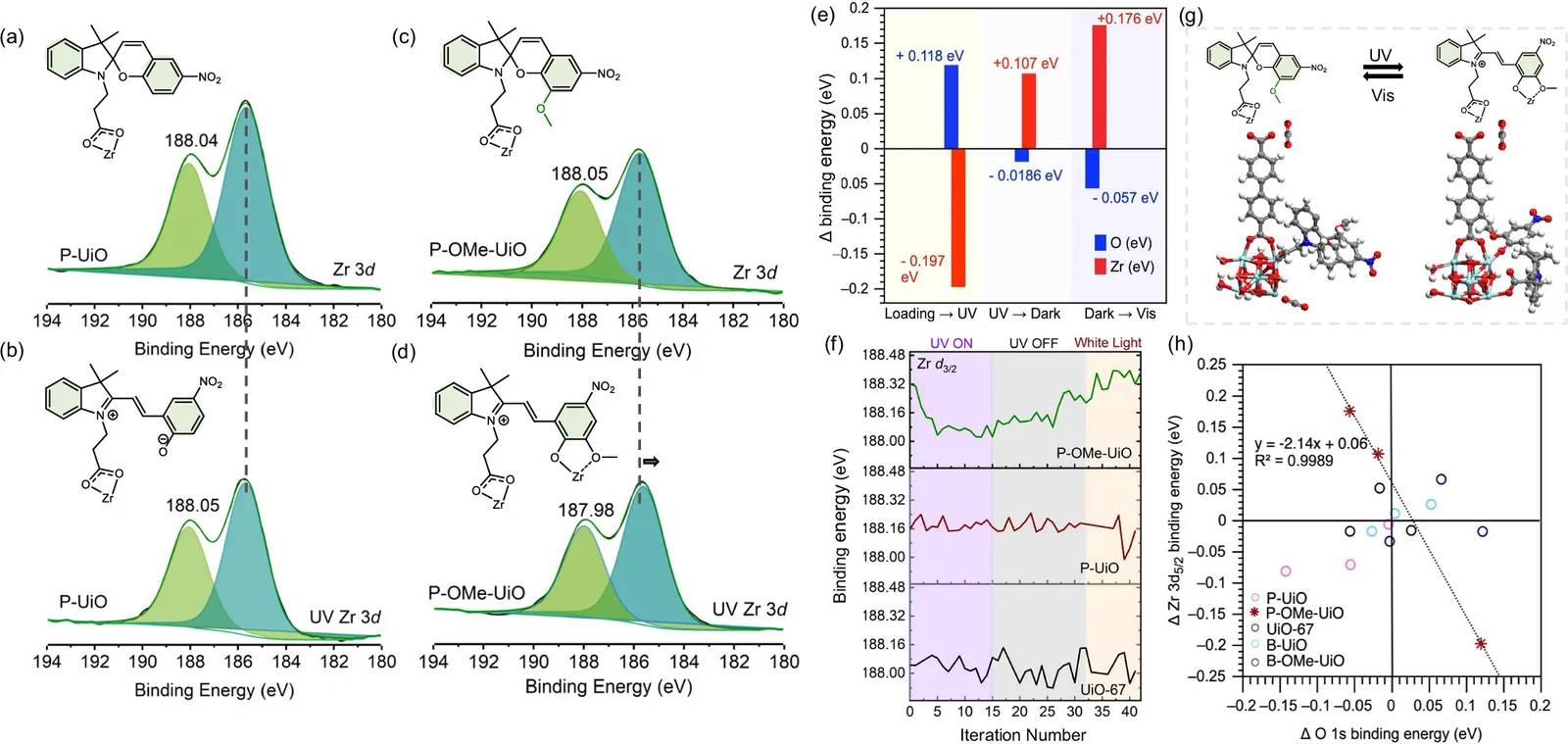
X-ray photoelectron spectroscopy (XPS) evidence for photo-reversible merocyanine (MC)-Zr complexation in P-OMe-UiO. The ex-situ Zr 3*d* XPS spectra of **a**, **b** P-UiO and **c**, **d** P-OMe-UiO before and after ultraviolet (UV) light exposure. The binding energy of Zr 3*d*_5/2_ in P-OMe-UiO was decreased by ~0.07 eV, while P-UiO remained unchanged. This indicated MC-Zr complexation in P-OMe-UiO after the spiropyran (SP) → MC transition. **e** Changes in binding energies of O 1*s* (blue) and Zr 3*d*_5/2_ (red) across three phase transitions: Load → UV, UV → Dark, Dark → visible light (Vis). The values are obtained by calculating the difference between the average O 1*s* and Zr 3*d*_5/2_ binding energies of different phase transitions. **f** In situ Zr 3*d* XPS spectra of parent defective UiO-67, P-UiO, and P-OMe-UiO with UV switched on/off and visible light irradiation. **g** Density functional theory (DFT)-optimized structures showing SP (left) and MC (right) monomers complexed to the Zr-oxo cluster, with MC exhibiting bidentate phenolate-zwitterion binding to open Zr site. **h** Opposite shifts between ΔZr 3*d*₅/₂ and ΔO 1*s* binding energies were only observed in P-OMe-UiO across all photo-induced phase transitions. Atoms in the molecular structures are color-coded as follows: C, gray; H, white; N, blue; Zr, cyan; and O, red.

The phenolate oxygen in MC can coordinate with metal ions and has been widely utilized for metal recovery and sensing in solutions^9,10^. A neighboring methoxy group can further enhance binding strength through cooperative interactions^42^. We therefore hypothesize that the methoxy substituent in P-OMe-UiO can facilitate photo-reversible complexation of MC with open Zr sites in defective UiO-67 MOFs. To test this hypothesis, we performed ex situ XPS to determine the shifts in binding energies of Zr and O core levels in P-OMe-UiO as a function of photo-irradiation wavelength. From the O 1*s* ex situ XPS spectra of P-OMe-UiO studied here (Fig. S19), we observed a distinct shift toward lower binding energies upon UV irradiation. This confirmed the ring-opening isomerization behavior of SP and the formation of phenoxy oxygen species. These were consistent with other SP-loaded systems^43,44^. The Zr 3*d* spectra of P-OMe-UiO comprised two characteristic peaks at around 185.50 eV and 188.0 eV, corresponding to Zr 3*d*_5/2_ and Zr 3*d*_3/2_, respectively (Fig. 4c). Upon UV irradiation, the binding energy of Zr 3*d*_5/2_ in P-OMe-UiO decreased by ~0.07 eV, while P-UiO remained unchanged. A decrease in the binding energy of Zr 3*d* can occur when Zr complexes with the phenolic oxygen and interacts with the nearby methoxy group. This was also in line with well-known SP–metal complexation behavior in solution^32^ and in other SP-loaded MOFs^11^. This validated the formation of an MC-Zr complex upon UV irradiation of the P-OMe-UiO MOF. We did not observe such trends from the ex-situ XPS spectra of P-UiO, B-UiO and B-OMe-UiO MOFs (Figs. 4a and S20–22). This showed that the formation of Zr-MC complexes upon UV irradiation was observed only for P-OMe-UiO studied in this work.

The reversible nature of MC-Zr complexation in P-OMe-UiO was clearly demonstrated by tracking the changes in its O 1*s* and Zr 3*d*_5/2_ binding energies across various phase transitions of photo-irradiation, from loading to UV-On, UV-On to Dark, and Dark to vis-irradiation, i.e., white-light-On (Fig. 4f and S23–25). During the Load→UV transition, the O 1*s* binding energy increased by +0.118 eV while the binding energy of Zr 3*d*_5/2_ decreased by −0.197 eV. The opposing shifts in O 1*s* and Zr 3*d*_5/2_ binding energies are direct evidence of MC-Zr complexation. This is because when the phenolate oxygen donates electron density to Zr, the oxygen becomes less electron-rich hence increasing O 1*s* binding energy. Meanwhile, when the Zr receives the electron density from the phenolate oxygen, Zr electron affinity is reduced. Thus, lowering Zr 3*d*_5/2_ binding energy. The UV → dark transition shows partial recovery of Zr 3*d*_5/2_ where this binding energy increased by 54 % (+0.107 eV). This was due to thermal dissociation of MC-Zr complexes, though O 1*s* binding energy remained relatively unchanged (−0.0186 eV, within standard deviation). The dark → white transition demonstrated further recovery as the Zr 3*d*_5/2_ binding energy increased by 0.176 eV upon vis-irradiation, as 90%–95% of the Zr nodes reverted to their original state. Reversible O 1*s* and Zr 3*d*_5/2_ binding energy shifts across successive light cycles directly confirm photo-reversible modulation of MC-Zr complexation in P-OMe-UiO.

Across three photo-induced phase transitions, O 1*s* and Zr 3*d*_5/2_ binding energies shifted in opposing directions across a complete photocycle (Load → UV → Dark → Vis), a trend observed only for P-OMe-UiO (Fig. 4h). The other SP-UiO MOFs studied, B-UiO, B-OMe-UiO and P-UiO, showed no opposing shifts. This pattern indicates that the effect in P-OMe-UiO is associated with Zr-MC complexation rather than instrument variation or a generic photo-response. Together, these results demonstrate that the N-alkyl carboxylic acid and methoxy substituent create the required geometric and electronic environment for photo-switchable MC-Zr complexation. The reversible formation of Zr–MC complexes in UV-irradiated P-OMe-UiO was further supported by DFT simulations (Fig. S4).

**Fig. 5:**
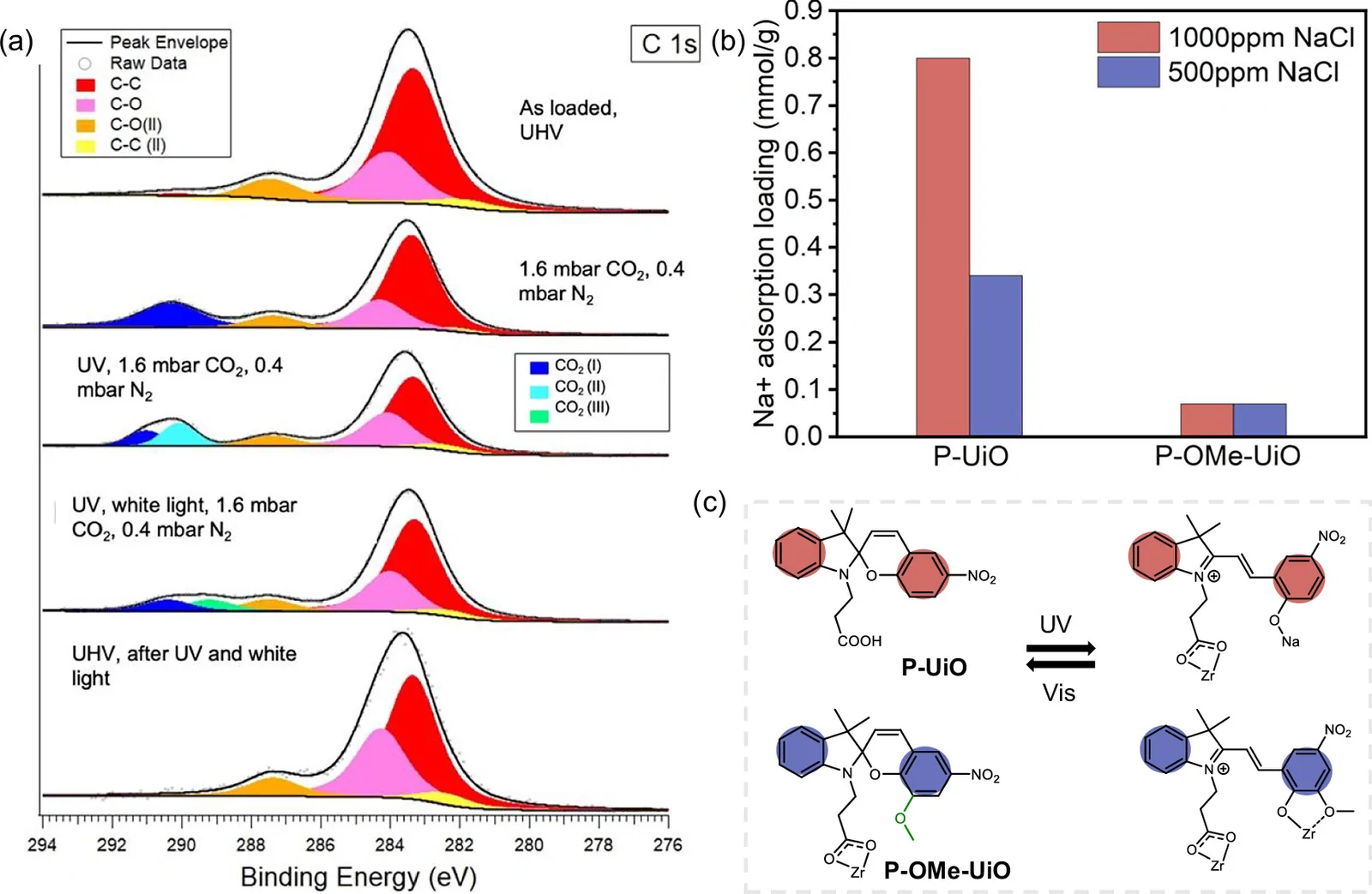
Evidence for defect complexation mechanisms. **a** near-ambient-pressure X-ray photoelectron spectroscopy (NAP-XPS) C 1*s* spectra tracking C 1*s* binding energies of CO_2_ under operando conditions: (i) P-OMe-UiO loaded in ultra-high vacuum (UHV). (ii) Exposing P-OMe-UiO to 1.6 mbar CO_2_/0.4 mbar N_2_ atmosphere. This mimicked CO_2_ adsorption in darkness. (iii) ultraviolet (UV) irradiation of CO_2_-loaded P-OMe-UiO in an atmosphere comprising 1.6 mbar CO_2_/0.4 mbar N_2_ gases. This mimicked using UV irradiation to lower CO_2_ adsorption and create a CO_2_ working capacity. (iv) visible-light (Vis) irradiation of CO_2_-loaded P-OMe-UiO under 1.6 mbar CO_2_/0.4 mbar N_2_. This mimicked regeneration of P-OMe-UiO. (v) After evacuation to UHV. UV irradiation released CO_2_ adsorption, as evidenced by increased peak intensity and complexity. **b** Na^+^ adsorption capacities (mmol g^−1^) of P-UiO and P-OMe-UiO in aqueous NaCl solutions with concentrations of 500 and 1000 ppm. **c** Schematic illustration of the photoresponsive behavior of P-UiO and P-OMe-UiO under UV and visible light.

The photo-responsive MC–Zr complexation mechanism reversibly switches open Zr sites between two electronic states, directly modulating their availability as binding sites. To verify this, we performed NAP XPS to track changes in CO_2_ binding energies as a function of the localized Zr environment (Fig. 5a). In defective MOFs, CO_2_ is typically adsorbed at open metal sites via carbonate formation^45^. Consistent with this, the peak of CO_2_ (I) of P-OMe-UiO centered at 290.3 eV confirms metal-carbonate formation. Upon UV irradiation, MC-Zr complexation renders the Zr sites more electronegative, as MC acts as an electron donor, pulling electron density towards Zr^38^. Carbonates formed at these more electronegative Zr-MC sites consequently exhibit higher binding energies^46^, shifting CO_2_(I) from 290.3 to 291 eV. Meanwhile, the binding energy of some carbon atoms (CO_2_ (II)) was reduced to 290 eV, indicative of weaker CO_2_-Zr interactions resulting from MC-Zr complexation. Visible light restores the CO_2_ binding energy to 290.3 eV via MC-to-SP isomerization. Additionally, ligand-to-metal charge transfer and direct photo-induced electron transfer to CO_2_ under visible light irradiation^47^ further reduce the binding energy to 289.2 eV (CO_2_ (III)). These results demonstrate that different light wavelengths modulate Zr-MC complexation and CO_2_ binding energy.

Besides regulating open Zr sites for CO₂ adsorption, photo-induced metal complexation also affects a second class of active sites in P-OMe-UiO. Upon UV irradiation, SP-based MOF systems undergo ring-opening isomerization to form MC, generating negatively charged phenolate oxygen centers capable of coordinating metal cations in solution^9^. In P-OMe-UiO, however, the photo-induced Zr–MC complexation occupies these sites, effectively suppressing their availability for metal-ion uptake. P-UiO, which lacks this intramolecular complexation pathway, retains full light-controlled metal adsorption and serves as an ideal reference to decouple these two competing mechanisms. Under UV irradiation, P-UiO adsorbed 0.84 and 0.34 mmol g⁻¹ of Na⁺ from 1000 and 500 ppm NaCl solutions, respectively, with complete Na⁺ release upon white-light irradiation (Fig. 5b). In contrast, P-OMe-UiO showed significantly lower Na^+^ uptake of only ~0.07 mmol g⁻¹ under identical conditions, consistent with MC-Zr complexation suppressing phenolate oxygen accessibility. These findings indicate that photo-modulated Zr–MC complexation operates across both gas phase and aqueous environments, establishing it as a general, light-programmable switch for active site availability (Fig. 5c)

### Light-induced swing adsorption of CO_2_

**Fig. 6:**
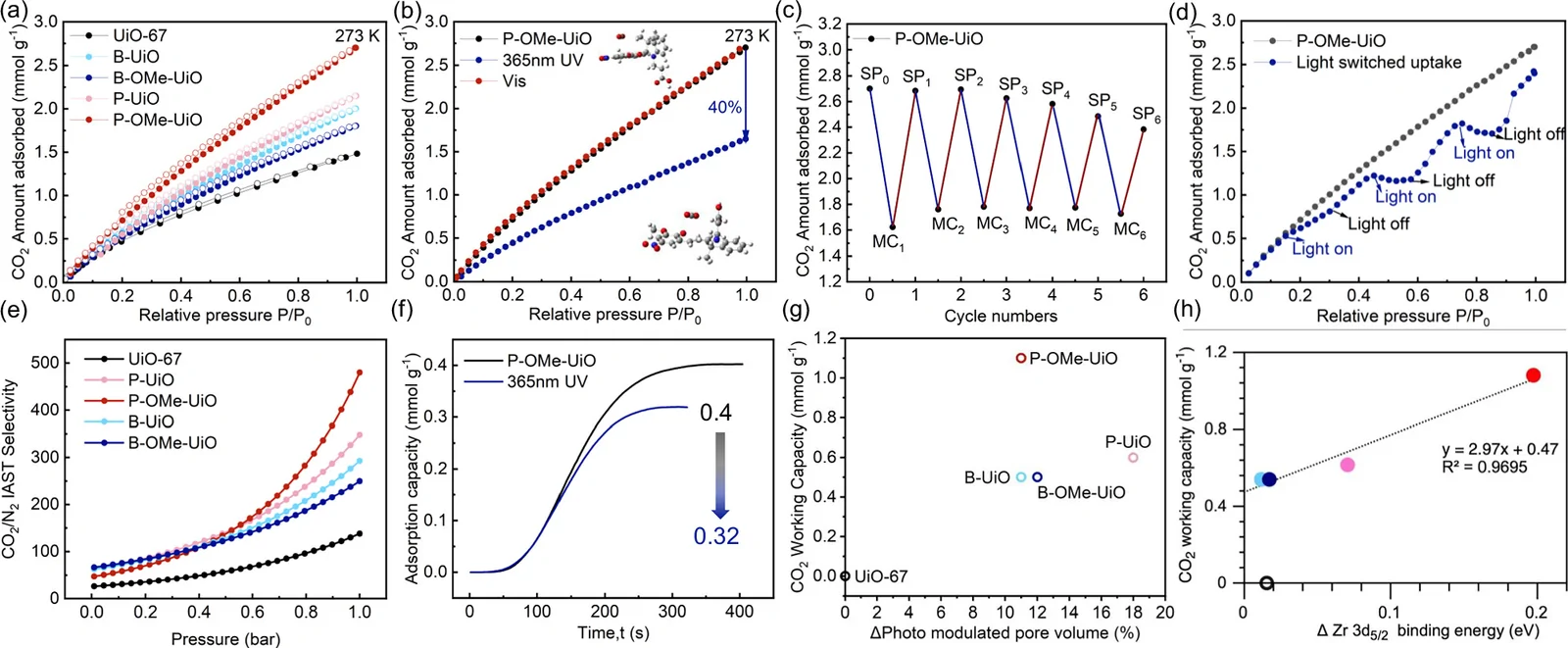
Dynamic CO₂ adsorption behavior of lead material. **a** CO_2_ adsorption–desorption isotherms of parent defective UiO-67 metal-organic framework (MOF) (black), P-UiO (pink), P-OMe-UiO (red), B-UiO (light blue), and B-OMe-UiO (deep blue) at 273 K. **b** CO_2_ adsorption isotherms of P-OMe-UiO at 273 K before irradiation, after ultraviolet (UV, 365 nm) and after visible-light (450 nm) irradiation. **c** CO_2_ cycling adsorption performance of P-OMe-UiO over multiple photoswitching cycles. **d** Real-time photoswitching monitored via CO_2_ uptake at 273 K showing response to alternating light exposure. Multiple light on (blue, 365 nm) and light off (black) cycles demonstrate reproducible switching. **e** Ideal adsorbed solution theory (IAST) CO_2_/N_2_ selectivity of all spiropyran (SP)-UiO samples calculated using 20:80 CO_2_ and N_2_ mixtures at 298 K. **f** CO_2_ adsorption capacities of UV irradiated (blue) and non-irradiated (black) P-OMe-UiO in breakthrough experiments using a binary gas mixture comprising 20:80 CO_2_ and N_2_. **g** CO_2_ working capacity (mmol g^−1^) vs. ΔTotal pore volume (%) of SP-UiOs show that there is no direct relationship between these parameters. **h** Linear relationship between CO₂ working capacities and |ΔZr 3*d*₅/₂| binding energies of SP-UiO MOFs during the Loading → UV transition shows that the modulation of Zr binding energy underpins CO_2_ working capacities.

With photo-reversible defect modulation mechanisms in place, we evaluated the CO_2_ adsorption capacities of these SP-UiO MOFs. Compared to the CO_2_ uptake in parent defective UiO-67 at 273 K, grafting **1**–**4** into UiO-67 enhanced CO_2_ uptakes by 38%–108% (Fig. 6a). These improvements in CO_2_ uptakes were due to three synergistic effects: (1) reducing structural defects in UiO-67 to enhance BET surface areas which provided more sorption sites^48^; (2) retaining open metal sites for specific strong interactions with CO_2_^49^; and (3) introducing tertiary amines, methoxy oxygens and nitro groups that also act as Lewis basic centers^50^. For B-UiO and B-OMe-UiO, these effects outweighed the 51%–57% loss in BET surface areas and total pore volumes in these materials, thus enhancing CO_2_ uptake when compared to the parent defective UiO-67 MOF.

At 273 K, exposure to UV/visible light generated a photo-reversible CO_2_ working capacity of 1.1 mmol g^−1^ in P-OMe-UiO, while the CO_2_ working capacity of P-UiO was only 0.6 mmol g^−1^. The presence of a methoxy group enables an 83% increase in working capacity that is also comparable to other reported similar materials (Table S3). In comparison, the CO_2_ uptake of the UV-irradiated parent defective UiO-67 MOF remained unchanged at 1.3 mmol g^−1^ and did not generate any CO_2_ working capacity as a function of light wavelength (Fig. S26b). We attributed the photo-modulated CO_2_ uptake in SP-UiOs to the presence of SP and not instrumental variation or MOF degradation. The photo-modulable CO_2_ working capacities of B-UiO and B-OMe-UiO studied were 50% lower than those of P-OMe-UiO (Fig. S27 and Table S2). This could be due to the location of **3** and **4** within the MOF framework. SP on the framework in P-UiO and P-OMe-UiO enhanced photo-isomerization efficiency and generated more pronounced pore volume changes upon UV exposure when compared to UiO-67, comprising SP located within the pores (B-UiO, B-OMe-UiO). The CO_2_ working capacity of P-OMe-UiO was higher than that of most of these materials (SI, Table S3).

To evaluate the feasibility of deploying P-OMe-UiO for light-regenerated carbon capture applications, we evaluated the cyclic performance of this material. The photo-modulated CO_2_ working capacity of P-OMe-UiO is sustained across six SP/MC switching cycles, retaining 88% of initial SP-state CO_2_ uptake (0.74 mmol g⁻¹ working capacity after 15 h cumulative UV irradiation). Post-cycling PXRD and FT-IR confirm full preservation of UiO-67 framework topology and chemical integrity, with all the progressive capacity reduction attributed to UV-induced photobleaching of the SP chromophore and MC aggregation rather than framework degradation^51^, with more pronounced decline beyond six cycles defining the current operational boundary. This cycling performance compares favorably with the majority of photo-responsive MOFs reported to date (Table S3) and could be further extended through incorporation of more photostable SP derivatives^52^ or polymer-tethered SP systems^9^ to suppress aggregation-induced degradation.

We also determined the dynamic light-triggered CO_2_ capture and release performance of P-OMe-UiO with in-situ CO_2_ adsorption measurements under alternating UV-light irradiation (Fig. 6d). At the initial stage, the CO_2_ uptake of P-OMe-UiO was identical to that measured in dark conditions. Upon UV irradiation, the CO_2_ uptake was reduced rapidly. This was consistent with the rapid SP-to-MC photo-isomerization within 5 s. In the absence of UV light, CO_2_ uptake of P-OMe-UiO was gradually recovered, indicating photo-reversible regeneration of this MOF. This light-controlled CO_2_ adsorption–desorption behavior could be cycled repeatedly three times, demonstrating responsiveness, reversibility, and operational stability under dynamic light exposure. This cyclic performance, combined with the ultrafast isomerization kinetics and sustainable external stimulus, offers a promising low-energy alternative to conventional CO₂ desorption techniques.

Other than CO_2_ uptake, selectivity towards CO_2_ over other gases is also important for practical CO_2_ separation applications^20,53^. At 298 K, all SP-UiOs MOF samples exhibited markedly lower N_2_ uptake, indicative of a preferential binding affinity for CO_2_ molecules and thus a high intrinsic CO_2_/N_2_ selectivity (Fig. S30). Based on this data, we calculated the ideal adsorbed solution theory (IAST) CO_2_/N_2_ selectivity of these materials for a 20:80 CO_2_ and N_2_ mixture at 298 K (Fig. 6e). The CO_2_/N_2_ selectivity of parent defective UiO-67 was the lowest amongst all MOFs studied here. Incorporation of various SP moieties not only enhanced the CO₂ uptake capacity but also significantly improved CO_2_/N_2_ selectivity. In the SP state, P-OMe-UiO achieves a CO_2_/N_2_ selectivity of 500, 51% higher than P-UiO, with negligible N_2_ adsorption in both SP and MC states. UV irradiation reduces selectivity to ~98, reflecting selective modulation of CO_2_ adsorption by MC-Zr complexation while N_2_ rejection is preserved.

To evaluate performance under realistic conditions, breakthrough experiments were conducted using a 20:80 CO_2_/N_2_ mixture (Figs. 6f and S31), building on the photo-modulated working capacity of 0.40 mmol g^−1^ and IAST selectivity of up to 500 at 298 K (Fig. S26a). In breakthrough tests, the CO_2_ uptake of P-OMe-UiO in dark conditions reached 0.40 mmol g^−1^, while the CO_2_ uptake of the same MOF was reduced to 0.32 mmol g^−1^ after UV irradiation. The photo-modulated CO_2_ adsorption capacities of P-OMe-UiO determined with a gas mixture were similar to those determined with pure CO_2_ gas at 0.2 bar. This showed that competitive sorption effects between CO_2_ and N_2_ that typically reduce the mixed gas CO_2_/N_2_ selectivity of MOFs^54^ were minimized. A cradle-to-gate LCA was conducted to evaluate and compare the environmental impacts associated with the operational energy consumption of LISA, temperature-swing adsorption (TSA), and pressure-swing adsorption (PSA) CO_2_ capture systems, and the functional unit defined as the amount of adsorbent required to capture 1 mol of CO_2_. The system boundary was confined to the operational phase, encompassing energy consumption associated with adsorption-desorption cycling and adsorbent regeneration. Key assumptions include constant adsorbent performance over repeated cycling and energy modeled using the UK electricity grid mix. Compared to traditional carbon capture processes like TSA and PSA, the LCA indicated that the environmental impact of carbon capture was significantly lower when light-induced swing adsorption enabled with P-OMe-UiO was used. This was due to the low energy demand of using P-OMe-UiO to create a CO_2_ working capacity, requiring only 60 J per adsorption-desorption cycle (240 J cumulative over four cycles) (Figs. S32 and 33; Tables S4–6).

Plotting photo-modulated CO_2_ working capacities against relative changes in photo-modulated total pore volumes revealed no correlation between these parameters (Fig. 6g). Despite losing 11% of its total pore volume upon UV irradiation, P-OMe-UiO exhibited the highest photo-modulated CO_2_ working capacity among all SP-UiO MOFs (1.1 mmol g^−1^). By contrast, P-UiO, B-UiO and B-OMe-UiO suffered pore volume losses of 11%–18%, yet their CO_2_ working capacities were 51%–54 % lower than those of P-OMe-UiO. This decoupling of pore volume loss from working capacity directly contradicted the conventional pore-accessibility logic, in which reduced accessible pore volumes are expected to reduce CO_2_ uptake^55^.

These findings suggested that the high CO_2_ working capacity of P-OMe-UiO was not governed by photo-reversible steric-induced pore accessibility^56^, but rather by photo-reversible complexation between MC phenolate oxygen groups and open Zr sites. MC-Zr complexation facilitated electron transfer from the phenolate oxygen to Zr, reducing access to the open metal sites and weakening CO_2_ affinity under UV irradiation. The resulting difference in CO_2_ adsorption capacity between UV-irradiated and non-irradiated states generated a working capacity of 1.1 mmol g^−1^, among the highest reported for photo-responsive MOFs.

To validate this mechanism, we plotted CO_2_ working capacities against UV-modulated changes in Zr 3*d*_5/2_ binding energies across all SP-UiO MOFs (Fig. 6h). A direct relationship between these two parameters was observed. P-OMe-UiO, which exhibited the largest Zr binding energy shift (0.197 eV), achieved the highest working capacity, while B-UiO, P-UiO, and B-OMe-UiO showed minimal Zr binding energy shifts and proportionally low working capacities. This demonstrated that the CO_2_ working capacity in SP-UiO MOFs is primarily controlled by photo-reversible defect complexation rather than steric factors alone, and confirmed that electron transfer from MC to Zr weakens CO_2_ binding during UV irradiation. This is the key mechanism underpinning large differentials between UV-on and Vis-on states and maximized photo-modulable CO_2_ capture performances.

Direct evidence for MC-Zr complexation reducing CO_2_ affinity was obtained from isosteric enthalpy of adsorption measurements, which are partially sensitive to site-specific binding interactions at pressures ≤1 bar. In the non-irradiated state, P-OMe-UiO exhibited an initial CO_2_ adsorption enthalpy of 45 kJ mol^−1^, which gradually decreased to a plateau (Fig. S34a). This is consistent with preferential adsorption at high-energy open Zr sites followed by accumulation in pore channels via weaker multilayer interactions^57^. Upon UV irradiation, the initial adsorption enthalpy reduced significantly to 16 kJ mol^−1^, indicating that the high-binding-energy open metal sites were no longer accessible for CO_2_ binding. This is consistent with SP-to-MC photoisomerization complexing with open metal Zr sites, causing CO_2_ adsorption to proceed exclusively at lower-energy sites and reducing overall uptake.

DFT calculations on optimized SP-UiOs cluster models provided further mechanistic insight (Table S7). In its non-irradiated state, P-OMe-UiO, which exhibited the highest CO_2_ uptake of 2.7 mmol g^−1^, had a CO_2_ binding energy of −30.9 kJ mol⁻¹, 63% lower than that of P-UiO (−18.9 kJ mol⁻¹). Although B-OMe-UiO showed a lower calculated binding energy (−39.6 kJ mol⁻¹), the CO_2_ uptakes of B-UiO and B-OMe-UiO were impeded by SP aggregation-induced losses in BET surface areas (51%–57%) and pore volume (53%–57%). These results confirm that pore accessibility governs uptake when binding energy differences are secondary.

While MC moieties typically exhibit higher CO_2_-binding interactions than SP isomers due to their higher dipole moment (MC + 14D vs. SP + 9D)^33^, this did not translate to higher CO_2_ uptake in the UV-irradiated MOFs studied here. In all SP-UiO MOFs, CO_2_ uptake was higher in the non-irradiated (SP-dominant) state than under UV irradiation (MC-dominant state) despite MC’s intrinsically stronger CO_2_ affinity. This energetic dominance of open-metal-site (OMS) blocking over MC polarity arises from two compounding effects. First, MC-Zr complexation directly engages the phenolate oxygen in a Zr-O coordination bond, simultaneously blocking the OMS and neutralizing the intrinsic polarity advantage of MC for CO_2_ interaction in P-OMe-UiO. Second, periodic DFT calculations across all four SP-UiO materials consistently yield lower CO_2_ binding energies in the MC state than the SP state (Table S8), confirming that the confined pore environment systematically prevents MC’s higher molecular dipole moment from translating into enhanced CO_2_ binding affinity. Together, these effects establish OMS blocking as the energetically dominant mechanism governing CO_2_ working capacity in P-OMe-UiO.

This interpretation is further corroborated by periodic DFT calculations comparing P-OMe-UiO with its parent defective UiO-67 framework (Table S8). With five missing linkers per metal node, the CO_2_ binding energy of the parent framework was –34.58 kJ mol^−1^. Incorporation of **2**, P-OMe-UiO increased this to –37.5 kJ mol^−1^. This 8 % increment in calculated binding energy is consistent with the 100% increase in experimental CO_2_ uptake relative to the parent. Upon photo-isomerization to the MC state, the binding energy of P-OMe-UiO decreased to −35.7 kJ mol⁻¹, reflecting the reduction in open Zr sites caused by MC-Zr complexation. This 40 % reduction in CO_2_ uptake under UV irradiation, observed only in P-OMe-UiO, is the mechanistic origin of its high working capacity that is nearly double those of all other MOFs in this series.

This work demonstrated photo-reversible defect engineering as a mechanism governing CO_2_ adsorption in SP-functionalised defective MOFs, distinct from conventional light-induced pore volume changes. Upon UV irradiation, MC phenolate oxygen groups complex with open Zr sites and weaken CO_2_ affinity. Visible light irradiation reverses complexation and restores uptake. This mechanism was validated with ex situ, in situ, and NAP XPS characterization, which revealed a direct relationship between UV-modulated Zr binding energy shifts and CO_2_ working capacities. This direct relationship between energy shifts and working capacities was validated by DFT simulations showing MC-Zr complexation reducing CO_2_ binding energies by blocking open Zr sites. The resulting CO_2_ working capacity of 1.1 mmol g^−1^ in P-OMe-UiO is among the highest reported for photo-responsive MOFs. These findings reframe defect engineering as a designable functional handle for stimuli-responsive porous materials and low-energy carbon capture applications.

## Methods

Materials: 3-methyl-2-butanone, 4-hydrazineyl benzoic acid, iodomethane, 2-hydroxy5-nitrobenzaldehyde, pyridine, 3-Methoxy-5-nitrosalicylaldehyde, trimethylindolenine, 3-iodopropanoic acid, triethylamine, piperdine, zirconium (IV) chloride, and biphenyl-4,4 -dicarboxylic acid were ordered from Merck. All purchased chemicals were used without further purification.

### Syntheses methods

2,3,3-trimethyl-3H-indole-5-carboxylic acid: A 50 mL round-bottom flask equipped with a reflux condenser were added 3-methyl-2-butanone (1.27 g, 14.79 mmol), 4-hydrazinylbenzoic acid (1.50 g, 9.85 mmol), and glacial acetic acid (20 mL). The reaction mixture was heated to reflux and stirred for 24 h. Upon completion, the reaction was cooled to room temperature, then quenched with deionized water (25 mL). The resulting mixture was extracted with dichloromethane (3 × 25 mL). The combined organic layers were dried over anhydrous sodium sulfate, filtered, and concentrated under reduced pressure to afford a red solid. ^1^H NMR (500 MHz, DMSO-*d*_6_) δ: 7.99 (s, 1H), 7.93–7.89 (d, 1H), 7.52–7.49 (d, 1H), 2.25 (s, 3H), 1.27 (s, 6H) ppm.

5-carboxy-1,2,3,3-tetramethyl-3H-indol-1-ium: To a stirred solution of *2,3,3-trimethyl-3H-indole-5-carboxylic acid* (6.50 g, 32.0 mmol) in acetonitrile (100 mL), iodomethane (11.3 g, 5.00 mL, 80 mmol) was added dropwise at room temperature. The reaction mixture was then heated to 45 °C and stirred for 12 h. After completion, the reaction was cooled to room temperature and diethyl ether (100 mL) was added. The resulting precipitate was collected by filtration, washed with diethyl ether (3 × 30 mL), and dried under reduced pressure to afford the product as a yellow solid (8.03 g, 72% yield). ^1^H NMR (500 MHz, DMSO-*d*_6_, δ/ppm): 8.38 (s, 1H), 8.18 (d, J = 8.3 Hz, 1H), 8.02 (d, J = 8.5 Hz, 1H), 3.99 (s, 3H), 3.10 (s, 1H), 2.77 (s, 2H), 1.57 (s, 3H), 1.27 (s, 3H).

**3**: To a 100 mL round-bottom flask equipped with a reflux condenser were added 5-carboxy-1,2,3,3-tetramethyl-3H-indol-1-ium (1.13 g, 3.27 mmol), 2-hydroxy-5-nitrobenzaldehyde (1.00 g, 5.98 mmol), pyridine (1.20 mL), and ethanol (15 mL). The reaction mixture was stirred under nitrogen and heated to reflux for 24 h. After cooling to room temperature, the mixture was allowed to stand overnight. The resulting precipitate was collected by filtration, washed with diethyl ether, and dried under vacuum to afford the desired product as a solid (980 mg, 82% yield). ^1^H NMR (500 MHz, DMSO-*d*_6_, δ/ppm): 12.35 (s, 1H), 8.25 (s, 1H), 8.03–8.00 (d, 1H), 7.80 (d, 1H), 7.68 (s, 1H), 7.25 (d, 1H), 6.90 (d, 1H), 6.70 (d, 1H), 6.03 (d, 1H), 2.75 (s, 3H), 1.24 (s, 3H), 1.13 (s, 3H).

**4**: To a 25 mL round-bottom flask equipped with a reflux condenser were added 5-carboxy-1,2,3,3-tetramethyl-3H-indol-1-ium (345 mg, 1.00 mmol), 3-methoxy-5-nitrosalicylaldehyde (360 mg, 1.83 mmol), pyridine (0.40 mL), and ethanol (10 mL). The reaction mixture was stirred under a nitrogen atmosphere and heated to reflux for 24 h. After cooling to room temperature, the mixture was allowed to stand overnight. The resulting precipitate was collected by filtration, washed with diethyl ether, and dried under vacuum to afford **4** as a yellow solid (150 mg, 40% yield). ^1^H NMR (500 MHz, DMSO-*d*_6_, δ/ppm): 12.35 (s, 1H), 7.90 (s, 1H), 7.80 (d, 1H), 7.70 (d, 1H), 7.23 (d, 1H), 6.73 (d, 1H), 6.00 (d, 1H), 3.77 (s, 3H), 2.75 (s, 3H), 1.25 (s, 3H), 1.13(s, 3H).

1-(2-carboxyethyl)-2,3,3-trimetyl-3H-indolium bromide Trimethylindolenine (10.1 mL, 60 mmol) and 3-bromopropanoic acid (9.2 g, 60 mmol) were added to a round-bottom flask and heated at 100 °C under a nitrogen atmosphere for 12 h. After cooling to room temperature, the resulting solid was dissolved in water. The aqueous layer was washed with chloroform (3 × 30 mL) to remove organic impurities. The water phase was then concentrated under reduced pressure to give the crude product as an oily liquid, which was used without further purification (75% yield). ^1^H NMR (400 MHz, DMSO-*d*_6_, δ/ppm): 1.53 (s, 6H, (CH_3_)_2_), 2.87 (s, 3H, N–CH_3_), 2.99 (t, 2H, –CH_2_–CH_2_–COOH), 4.66 (t, 2H, N–CH_2_–), 7.60–7.65 (m, 2H, Ar–H), 7.83–7.86 (m, 1H, Ar–H), 7.99–8.02 (m, 1H, Ar–H).

**1**: A solution of 1-(2-carboxyethyl)-2,3,3-trimethyl-3H-indolium bromide (7.35 g, 23.55 mmol), 5-nitrosalicylaldehyde (3.58 g, 21.40 mmol), and triethylamine (4.5 mL, 32.0 mmol) in dry ethanol (70 mL) was stirred under an argon atmosphere and heated to reflux for 34 h. Upon completion, the resulting precipitate was collected by filtration, washed with cold ethanol (2 × 10 mL), and dried under vacuum to afford 1 as a yellow powder (6.00 g, 73.8% yield). ^1^H NMR (400 MHz, DMSO-*d*_6_, δ / ppm): 12.22 (s, 1H), 8.22 (s, 1H), 8.00 (d, *J* = 9.0 Hz, 1H), 7.21 (d, *J* = 10.4 Hz, 1H), 7.12 (d, *J* = 7.0 Hz, 2H), 6.87 (d, *J* = 9.0 Hz, 1H), 6.80 (t, *J* = 7.3 Hz, 1H), 6.67 (d, *J* = 7.7 Hz, 1H), 6.00 (d, *J* = 10.4 Hz, 1H), 3.49 (m, 2H), 2.61–2.53 (m, 1H), 2.48–2.41 (m, 1H), 1.19 (s, 3H), 1.08 (s, 3H).

**2**: A solution of 1-(2-carboxyethyl)-2,3,3-trimethyl-3H-indolium bromide (312 mg, 1.00 mmol), 3-methoxy-5-nitrosalicylaldehyde (285 mg, 1.45 mmol), and piperidine (0.15 mL) in dry ethanol (15 mL) was stirred under an argon atmosphere and heated to reflux for 24 h. After cooling to room temperature, the solvent was removed under reduced pressure. The crude residue was purified by flash column chromatography (hexane/ethyl acetate = 3:1) to afford 2 as a purple solid (200 mg, 50% yield). ^1^H NMR (400 MHz, DMSO-*d*_6_, δ/ppm): 12.22 (s, 1H), 8.27 (s, 1H), 7.85 (s, 1H), 7.70 (s, 1H), 7.20 (m, 2H), 6.78 (m, 1H), 6.67 (d, 1H), 5.95 (d, 1H), 3.74 (m, 3H), 2.90 (m, 2H), 2.60 (m, 2H), 1.19 (s, 3H), 1.08 (s, 3H).

Synthesis of defective UiO-67: In an 8-dram vial, zirconium(IV) chloride (134 mg) was dissolved in N,N-dimethylformamide (DMF, 10 mL) and concentrated hydrochloric acid (1.00 mL) by sonicating the mixture for 20 min. Biphenyl-4,4′-dicarboxylic acid (180 mg) was then added along with an additional 20 mL of DMF, and the mixture was sonicated for another 20 min. The resulting solution was heated at 90 °C overnight. After cooling to room temperature, the solid product was collected by filtration through a fine frit and sequentially washed with DMF (2 × 30 mL) and ethanol (2 × 30 mL). The obtained UiO-67 was dried under vacuum at 90 °C.

Post-synthetic functionalization of UiO-67: For post-synthetic functionalization, UiO-67 (25 mg) was transferred to a 20 mL vial, and 1.00 mL of a 30.0 mM DMF solution of the desired SP compound was added. The mixture was placed in a preheated oven at 90 °C and incubated for 24 h. After cooling to room temperature, the resulting powder was collected by centrifugation, washed thoroughly with DMF (3 × 10 mL) and ethanol (3 × 10 mL), and dried in a vacuum oven.

### Characterization

^1^H NMR spectra were recorded on a 400 MHz Bruker spectrometer. FTIR spectra were recorded on a Shimadzu IRTracer-100 spectrometer over 500–4000 cm^−1^. UV-vis diffuse reflectance spectroscopy (DRS) was performed at room temperature using a JASCO V-670 spectrophotometer with BaSO_4_ as reference over 200–800 nm. Ex situ XPS spectra were acquired using a ThermoFisher K-Alpha X-ray photoelectron spectrometer. Nitrogen adsorption-desorption isotherms at 77 K and CO_2_ adsorption–desorption isotherms at 273 and 298 K were measured using a Quadrasorb Evo (Quantachrome); samples were degassed under vacuum at 90 °C for 12 h before analysis. Pore size distributions were calculated using the non-local density functional theory (NLDFT) model. TGA was performed on a TA Instruments Trios V5 system, PXRD patterns were recorded on a Bruker D8 Advance diffractometer equipped with a Sol-X energy-dispersive detector, and SEM images were obtained using a Cryo focused ion beam–scanning electron microscopy (FIB-SEM) system. The photoswitching behavior of SP-UiO samples was evaluated by UV-vis DRS. Finely ground samples were pressed onto BaSO_4_, irradiated with UV light for several seconds, and spectra were recorded before and after UV exposure. The irradiated samples were subsequently exposed to visible light for several minutes and remeasured to assess reversibility. To evaluate porosity changes, N_2_ isotherms were measured at 77 K after overnight degassing at 90 °C. The same sealed sample cell was irradiated uniformly with UV light for 1 h and reanalyzed immediately without additional heating. Reversibility was assessed after re-degassing the UV-treated sample at 100 °C overnight.

SP contents were determined by combining TGA-derived BPDC/Zr ratios with ^1^H NMR analysis of digested samples. SP-UiO samples (2 mg) were digested with 50.0 μL D_2_SO_4_, diluted with 500 μL DMSO-*d*_6_, sonicated for 10 min and transferred to NMR tubes. Integrated BPDC and SP signals were used together with the BPDC/Zr ratio to calculate SP content.

In-situ light-responsive CO_2_ adsorption experiments: A custom-built BET light cell containing the sample tube and an optical-fiber light guide was used for in-situ irradiation (Fig. S1)^24^. A Julabo refrigerated circulator maintained the temperature at 273 or 298 K. Samples were degassed under vacuum at 90 °C overnight, and baseline CO_2_ adsorption–desorption isotherms were measured using a Quadrasorb Evo. Samples were then irradiated with 365 nm UV light and analyzed under continuous UV irradiation. After measurement, the cell was sealed and exposed to visible light (white light-emitting diode) before the CO_2_ isotherm was remeasured under identical conditions. UV/visible-light switching was repeated three times.

IAST CO_2_/N_2_ selectivity: Ideal adsorbed solution theory (IAST) selectivities for a 20:80 CO_2_/N_2_ mixture at 298 K were calculated from the single-component CO_2_ and N_2_ isotherms, each fitted with a single-site Langmuir–Freundlich model over the full pressure range (0–1.0 bar). The adsorbed- and bulk-phase mole fractions used to evaluate the selectivity were obtained using the IAST of Myers and Prausnitz^58^.

Isosteric enthalpy of adsorption: The isosteric enthalpy of adsorption (*Q*_st_) for CO_2_ was determined by Clausius–Clapeyron analysis of CO_2_ adsorption isotherms collected at 273 and 298 K for all SP-UiO samples, both before and after UV irradiation. Isotherms were fitted with a dual-site Langmuir model prior to the analysis so that the isosteric heat could be interpolated at consistent loading points across both temperatures.

CO_2_ breakthrough tests: P-OMe-UiO (400 mg) was packed into a quartz tube and challenged with a simulated flue-gas mixture containing 20 vol% CO_2_ in N_2_. Outlet CO_2_ concentration was monitored continuously. A schematic of the breakthrough configuration is provided in Fig. S2^59^.

X-ray photoelectron spectroscopy: In-situ XPS measurements were performed using a SPECS Phoibos 100 analyzer with a 2D detector and a PreVac twin-anode Al Kα source at a base pressure of 5 × 10^−10 ^mbar. Core-level spectra were collected with a 20 eV pass energy. Real-time measurements used 500 detector channels in snapshot mode, a 60 eV pass energy, and a 10 eV window with 0.02 eV step size; three snapshots were acquired every 6 s and averaged for each cycle. UV and visible light were delivered with a Bentham TLS120Xe tunable light source through focusing optics. NAP-XPS was conducted using a SPECS Phoibos 150 1DLD NAP analyzer and a monochromated Al Kα source. Premixed N_2_/CO_2_ gases (>99.9999% purity) were introduced through a needle valve after backfilling the analysis chamber to the required pressure. Core-level spectrum was measured at 20 eV pass energy. Sample temperature was monitored during irradiation, and no thermal change was observed within ±0.1 °C; conductive mounting and the X-ray source were used to suppress charging effects.

Ion adsorption and desorption: NaCl solutions (1000 and 500 ppm) were used for ion-adsorption measurements. P-UiO or P-OMe-UiO (10 mg) was dispersed in 10.00 mL solution and stirred under UV light for 2 h. The suspension was centrifuged and the supernatant filtered for inductively coupled plasma optical emission spectroscopy (ICP-OES) analysis. Desorption was subsequently carried out in deionized water under white-light irradiation with stirring for 1 h and the released metal ions were quantified by ICP-OES.

### Computation

Computational and molecular simulations: The initial UiO-67 crystallographic structure^60^ was used to construct a defective model containing approximately seven linkers per metal-oxide cluster in Materials Studio Visualizer^61^. The structure was optimized with the FORCITE module using Universal Force Field (UFF)^62^ parameters and charge-equilibration (QEq) charges. SP-functionalized models were generated by replacing selected linkers in accordance with the experimentally determined SP contents. Representative model structures are provided in Figs. S3 and S4.

Periodic geometry optimizations and static CO_2_ binding energies were calculated using the Vienna Ab initio Simulation Package (VASP) 5.4.4 with the Perdew–Burke–Ernzerhof (PBE) generalized-gradient functional^63^, DFT-D3 dispersion correction^64^, and projector-augmented-wave (PAW) potentials^65^. A plane-wave cutoff of 520 eV and a 1 × 1 × 1 Gamma-point mesh were used, and structures were relaxed until the Hellmann-Feynman forces were below 0.02 eV Å^−1^. Initial CO_2_ configurations were obtained by classical simulated annealing in the Materials Studio Sorption module^61^ using Lennard–Jones and Coulombic interactions with UFF parameters, followed by DFT binding-energy calculations. UV-visible absorption spectra were calculated from cluster models optimized with the M06-2X functional, 6-311 G + +(d,p) basis set, and a solvation model based on density (SMD) implicit solvent model (DMSO) using Gaussian 09^66^, followed by TD-DFT calculations at the same level of theory. Pore-size distributions were calculated using PoreBlazer^67^ with a probe radius corresponding to the kinetic diameter of N_2_.

Lifecycle assessment methodology: The lifecycle assessment compared operational energy consumption for LISA, TSA, and PSA. The functional unit was defined as the amount of adsorbent required to capture 1 mol of CO_2_, using P-OMe-UiO for LISA and UiO-67 for TSA and PSA^68^. The system boundary was limited to adsorption-desorption cycling and regeneration; adsorbent synthesis, equipment manufacture, infrastructure, and end-of-life treatment were excluded. Energy demand was modeled using the United Kingdom electricity grid mix in SimaPro v9.0. Thermal regeneration for TSA was represented by electrical heating for consistency. Constant adsorbent performance, ideal energy transfer unless otherwise reported, and operational energy as the dominant contribution were assumed. Working capacities and per-cycle energy demands^68^ were used to calculate the number of cycles and total energy required per functional unit. Environmental impacts were evaluated using the ReCiPe 2016 Midpoint (H) method with the default background datasets and characterization factors.

## Supplementary information

Supplementary Information of Photoreversible Defect Complexation Dynamically Modulates Guest Adsorption in Metal-Organic Frameworks

Transparent Peer Review file

## Data Availability

The data that support the findings of this study are available within the paper and its Supplementary Information. Source Data are provided with this paper. No custom code was generated in this study.
