# Supplementary material for: Photoreversible defect complexation dynamically modulates guest adsorption in metal-organic frameworks
Source: Nat Commun. 2026 Oct 15;17:11629. doi: 10.1038/s41467-026-78352-9 (PMC13754871; doi:10.1038/s41467-026-78352-9)
Supplement: Supplementary file 1 [file 41467_2026_78352_MOESM1_ESM.pdf]

Supplementary Information

Photoreversible Defect Complexation Dynamically Modulates Guest Adsorption in Metal-Organic Frameworks

Qi Huang* [a], Simon Astley [b], Nikita Rog [a], Boon Kee Voon [g], Tushar Singh Verma [c, e, f], Wenyu Shen [a], Ruixue Sun [a], Wai Fen Yong [g, h], Ravichandar Babarao [c, d], Yi Huang [a], Xianfeng Fan [a], Cher Hon Lau *[a]

[a] Q. Huang*, N. Rog, W. Shen, R. Sun, Y. Huang, X. Fan, C. H. Lau*

School of Engineering, The University of Edinburgh, Robert Stevenson Road, Edinburgh, EH9 3BF, UK.

E-mail: qhuang3@ed.ac.uk

E-mail: cherhon.lau@ed.ac.uk

[b] S. Astley

Department of Physics, Aberystwyth University, Aberystwyth, SY23 3BZ, UK.

[c] T. Verma, R. Babarao

Centre for Advanced Materials & Industrial Chemistry (CAMIC) and ARC Centre of Excellence for Electrochemical Transformation of Carbon Dioxide, School of Science, RMIT University, Melbourne, VIC 3001, Australia

[d] R. Babarao

Commonwealth Scientific and Industrial Research Organisation (CSIRO), Normanby Road, Clayton, VIC 3168, Australia.

[e] T. Verma

Academy of Scientific and Innovative Research (AcSIR), Ghaziabad, Uttar Pradesh, 201 002 India

[f] T. Verma

Physical Chemistry Division, CSIR-National Chemical Laboratory, Pune, 411008 India

[g] B. Voon, W. Yong

School of Energy and Chemical Engineering, Centre of Excellence for Green and Advanced Technologies in Efficient Separations (GATES), Xiamen University Malaysia, Jalan Sunsuria, Bandar Sunsuria, 43900 Sepang, Selangor, Malaysia.

[h] W. Yong

State Key Laboratory of Physical Chemistry of Solid Surfaces, College of Chemistry and Chemical Engineering, Xiamen University, Xiamen 361005, China

Table of Contents

1. Experimental details 2

1.1 Materials 2

1.2 Syntheses methods 2

1.3 Characterization techniques 6

1.4 Computational and molecular simulations 11

1.5 Lifecycle Assessment (LCA) methodology 14

2. Supplementary Figures and Tables 16

2.1 Physicochemical characterization of SP-UiO MOFs 16

2.2 Photo-responsive properties of SP-UiO MOFs 24

2.3 CO_2 adsorption isotherms of SP-UiO MOFs 31

2.4 Properties of SP-UiO MOFs after cyclic testing 34

2.5 Detailed discussion of LCA outcomes 38

2.6 Photo-reversible electronic modulation of P-OMe-UiO Lewis acidity 27

.... 29

3. Supplementary Tables 41

4. References 46

1. Experimental details

1.1 Materials

3-methyl-2-butanone, 4-hydrazineyl benzoic acid, iodomethane, 2-hydroxy5-nitrobenzaldehyde, pyridine, 3-Methoxy-5-nitrosalicylaldehyde, trimethylindolenine, 3-iodopropanoic acid, triethylamine, piperdine, zirconium(IV) chloride and biphenyl-4,4 -dicarboxylic acid were ordered from Merck. All purchased chemicals were used without further purification.

1.2 Syntheses methods

1.2.1 Synthesis of various spiropyran (SP)-based ligands

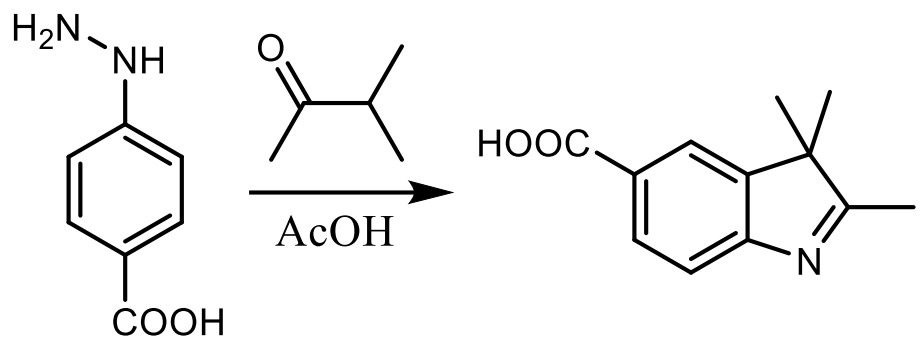

2,3,3-trimethyl-3H-indole-5-carboxylic acid To a 50 mL round-bottom flask equipped with a reflux condenser were added 3-methyl-2-butanone (1.27 g, 14.79 mmol), 4-hydrazinylbenzoic acid (1.50 g, 9.85 mmol), and glacial acetic acid (20 mL). The reaction mixture was heated to reflux and stirred for 24 h. Upon completion, the reaction was cooled to room temperature, then quenched with deionized water (25 mL). The resulting mixture was extracted with dichloromethane (3 × 25 mL). The combined organic layers were dried over anhydrous sodium sulfate, filtered, and concentrated under reduced pressure to afford a red solid.
^{1}H NMR (500 MHz, DMSO-d_6) δ: 7.99 (s, 1H), 7.93–7.89 (d, 1H), 7.52–7.49 (d, 1H), 2.25 (s, 3H), 1.27 (s, 6H) ppm.

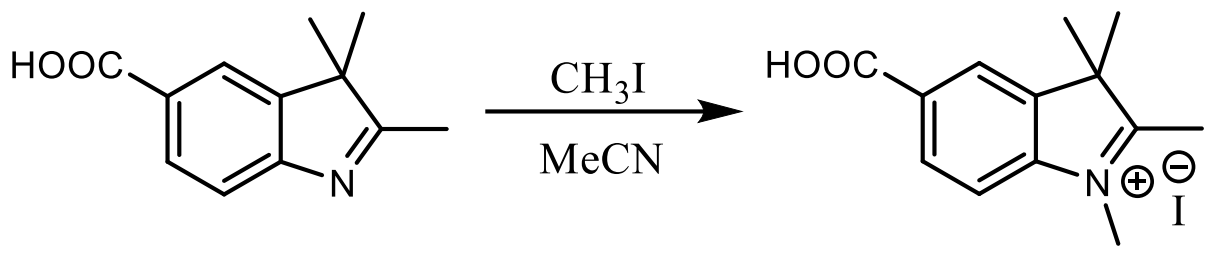

5-carboxy-1,2,3,3-tetramethyl-3H-indol-1-ium: To a stirred solution of *2,3,3-trimethyl-3H-indole-5-carboxylic acid* (6.50 g, 32.0 mmol) in acetonitrile (100 mL), iodomethane (11.3 g, 5.00 mL, 80 mmol) was added dropwise at room temperature. The reaction mixture was then heated to 45 °C and stirred for 12 h. After completion, the reaction was cooled to room temperature and diethyl ether (100 mL) was added. The resulting precipitate was collected by filtration, washed with diethyl ether (3 × 30 mL), and dried under reduced pressure to afford the product as a yellow solid (8.03 g, 72% yield).
^{1}H NMR (500 MHz, DMSO-d_6, δ / ppm): 8.38 (s, 1H), 8.18 (d, J = 8.3 Hz, 1H), 8.02 (d, J = 8.5 Hz, 1H), 3.99 (s, 3H), 3.10 (s, 1H), 2.77 (s, 2H), 1.57 (s, 3H), 1.27 (s, 3H).

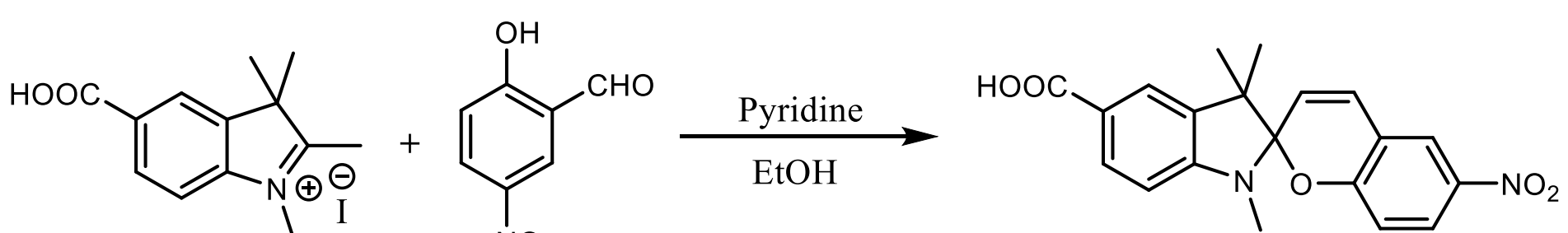

3

3: To a 100 mL round-bottom flask equipped with a reflux condenser were added 5-carboxy-1,2,3,3-tetramethyl-3H-indol-1-ium (1.20 g, 3.27 mmol), 2-hydroxy-5-nitrobenzaldehyde (1.00 g, 5.98 mmol), pyridine (1.20 mL), and ethanol (15 mL). The reaction mixture was stirred under nitrogen and heated to reflux for 24 h. After cooling to room temperature, the mixture was allowed to stand overnight. The resulting precipitate was collected by filtration, washed with diethyl ether, and dried under vacuum to afford the desired product as a solid (980 mg, 82% yield).
^{1}H NMR (500 MHz, DMSO-d_6, δ / ppm): 12.35 (s, 1H), 8.25 (s, 1H), 8.03–8.00 (d, 1H), 7.80 (d, 1H), 7.68 (s, 1H), 7.25 (d, 1H), 6.90 (d, 1H), 6.70 (d, 1H), 6.03 (d, 1H), 2.75 (s, 3H), 1.24

(s, 3H), 1.13 (s, 3H).

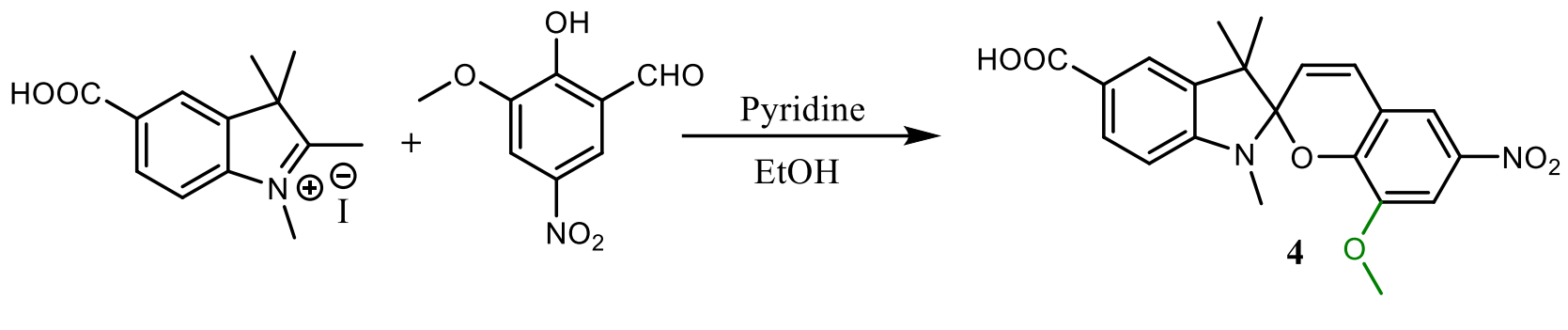

4: To a 25 mL round-bottom flask equipped with a reflux condenser were added 5-carboxy-1,2,3,3-tetramethyl-3H-indol-1-ium (218 mg, 1.00 mmol), 3-methoxy-5-nitrosalicylaldehyde (360 mg, 1.83 mmol), pyridine (0.40 mL), and ethanol (10 mL). The reaction mixture was stirred under a nitrogen atmosphere and heated to reflux for 24 h. After cooling to room temperature, the mixture was allowed to stand overnight. The resulting precipitate was collected by filtration, washed with diethyl ether, and dried under vacuum to afford **4** as a yellow solid (150 mg, 40% yield).

^{1}H NMR (500 MHz, DMSO-d_6, δ / ppm): 12.35 (s, 1H), 7.90 (s, 1H), 7.80 (d, 1H), 7.70 (d, 1H), 7.23 (d, 1H), 6.73 (d, 1H), 6.00 (d, 1H), 3.77 (s, 3H), 2.75 (s, 3H), 1.25 (s, 3H).

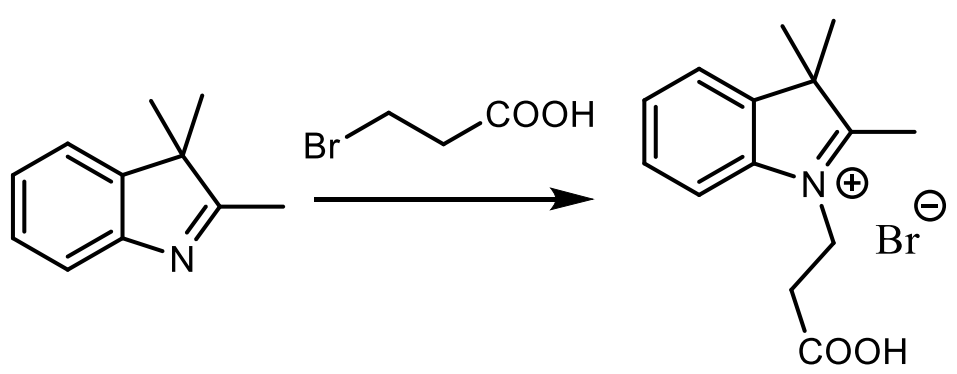

1-(2-carboxyethyl)-2,3,3-trimetyl-3H-indolium bromide Trimethylindolenine (10.1 mL, 60 mmol) and 3-iodopropanoic acid (12.6 g, 60 mmol) were added to a round-bottom flask and heated at 100 °C under a nitrogen atmosphere for 12 h. After cooling to room temperature, the resulting solid was dissolved in water. The aqueous layer was washed with chloroform (3 × 30 mL) to remove organic impurities. The water phase was then concentrated under reduced pressure to give the crude product as an oily liquid, which was used without further purification (75% yield).

^{1}H NMR (400 MHz, DMSO-d_6, δ / ppm): 1.53 (s, 6H, $(CH_3)_2$), 2.87 (s, 3H, N–CH_3), 2.99 (t, 2H, –CH_2–CH_2–COOH), 4.66 (t, 2H, N–CH_2–), 7.60–7.65 (m, 2H, Ar–H), 7.83–7.86 (m, 1H, Ar–H), 7.99–8.02 (m, 1H, Ar–H).

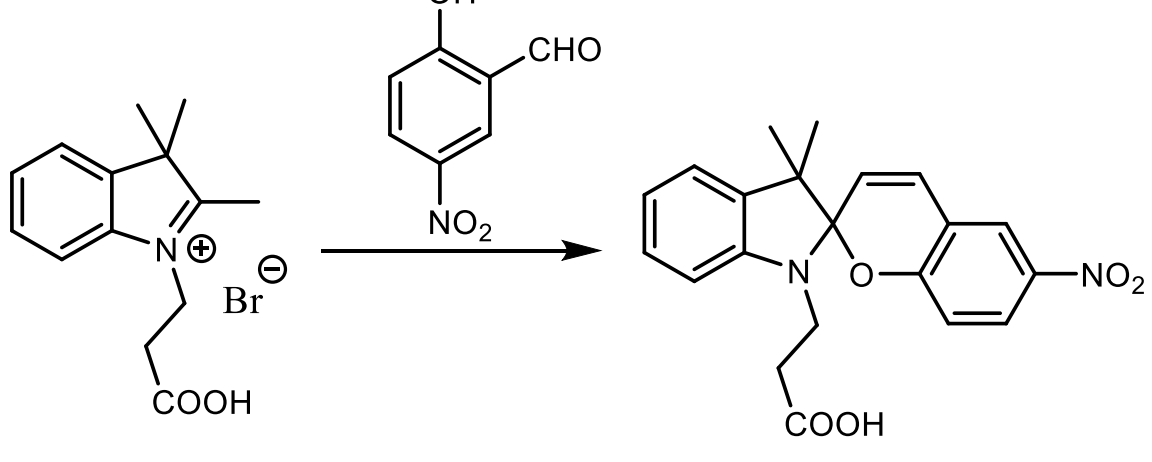

1

1 A solution of 1-(2-carboxyethyl)-2,3,3-trimethyl-3H-indolium bromide (7.35 g, 23.55 mmol), 5-nitrosalicylaldehyde (3.58 g, 21.40 mmol), and triethylamine (4.5 mL, 32.0 mmol) in dry ethanol (70 mL) was stirred under an argon atmosphere and heated to reflux for 34 h. Upon completion, the resulting precipitate was collected by filtration, washed with cold ethanol (2 ×

10 mL), and dried under vacuum to afford 1 as a yellow powder (6.00 g, 73.8% yield).

^{1}H NMR (400 MHz, DMSO-d_6, δ / ppm): 12.22 (s, 1H), 8.22 (s, 1H), 8.00 (d, J = 9.0 Hz, 1H), 7.21 (d, J = 10.4 Hz, 1H), 7.12 (d, J = 7.0 Hz, 2H), 6.87 (d, J = 9.0 Hz, 1H), 6.80 (t, J = 7.3 Hz, 1H), 6.67 (d, J = 7.7 Hz, 1H), 6.00 (d, J = 10.4 Hz, 1H), 3.49 (m, 3H), 2.61–2.53 (m, 1H), 2.48–2.41 (m, 1H), 1.19 (s, 3H), 1.08 (s, 3H).

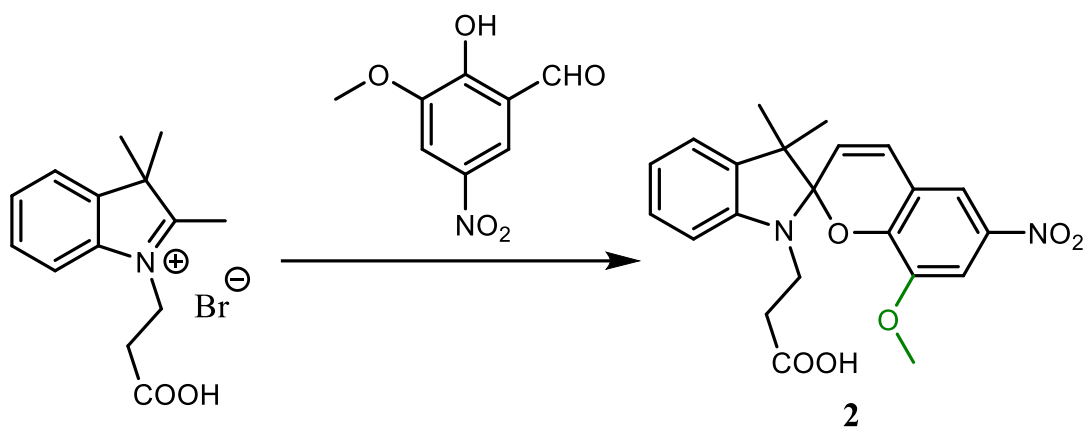

2

2 A solution of 1-(2-carboxyethyl)-2,3,3-trimethyl-3H-indolium bromide (312 mg, 1.00 mmol), 3-methoxy-5-nitrosalicylaldehyde (285 mg, 1.45 mmol), and piperidine (0.15 mL) in dry ethanol (15 mL) was stirred under an argon atmosphere and heated to reflux for 24 h. After cooling to room temperature, the solvent was removed under reduced pressure. The crude residue was purified by flash column chromatography (hexane/ethyl acetate = 3:1) to afford 2 as a purple solid (200 mg, 50% yield).

^{1}H NMR (400 MHz, DMSO-d_6, δ / ppm): 12.22 (s, 1H), 8.27 (s, 1H), 7.85 (s, 1H), 7.70 (s, 1H), 7.20 (m, 2H), 6.78 (m, 1H), 6.67 (d, 1H), 5.95 (d, 1H), 3.74 (m, 3H), 2.90 (m, 2H), 2.60 (m, 2H), 1.19 (s, 3H), 1.08 (s, 3H).

1.2.2 Synthesis of defective UiO-67

In an 8-dram vial, zirconium(IV) chloride (134 mg) was dissolved in N,N-dimethylformamide (DMF, 10 mL) and concentrated hydrochloric acid (1.00 mL) by sonicating the mixture for 20 min. Biphenyl-4,4′-dicarboxylic acid (180 mg) was then added along with an additional 20 mL of DMF, and the mixture was sonicated for another 20 min. The resulting solution was heated at 90 °C overnight. After cooling to room temperature, the solid product was collected by filtration through a fine frit and sequentially washed with DMF (2 × 30 mL) and ethanol (2 × 30 mL). Except for supercritically dried samples, the obtained UiO-67 was dried under vacuum at 90 °C.

1.2.3 Post-synthetic functionalization of UiO-67

For post-synthetic functionalization, UiO-67 (25 mg) was transferred to a 20 mL vial, and 1.00 mL of a 30.0 mM DMF solution of the desired SP compound was added. The mixture was placed in a preheated oven at 90 °C and incubated for 24 h. After cooling to room temperature, the resulting powder was collected by centrifugation, washed thoroughly with DMF (3 × 10 mL) and ethanol (3 × 10 mL), and dried in a vacuum oven.

1.3 Characterization techniques

1.3.1 Characterization of SP-based ligands

^{1}H NMR spectra were recorded on a 400 MHz Bruker spectrometer equipped with an automated tune-and-match multi-nuclei probe. Fourier-transform infrared (FTIR) spectra were recorded on a Shimadzu IRTracer-100 spectrometer over the range of 500–4000 cm^{-1}. The photoisomerization of 1 – 4 in solution (organic media) was characterized using UV–vis diffuse reflectance spectroscopy (DRS). DRS was performed at room temperature using a JASCO V-670 spectrophotometer with $BaSO_4$ as the reference standard; spectra were collected over the range of 200–800 nm.

1.3.2 Characterization of SP-UiOs

FTIR spectra were recorded on a Shimadzu IRTracer-100 spectrometer over the range of 500–4000 cm^{-1}. Ex-situ X-ray photoelectron spectroscopy valence-band (XPS-VB) spectra were obtained using a ThermoFisher K-Alpha X-ray photoelectron spectrometer to analyze the electronic structure and chemical states of the elements in SP-UiO samples. Nitrogen adsorption–desorption isotherms at 77 K were measured using a Quadrasorb Evo instrument (Quantachrome). Pore size distributions and pore volumes of these metal-organic frameworks (MOFs) were calculated using the non-local density functional theory (NLDFT) model. CO_2 adsorption–desorption isotherms were collected at 273 K and 298 K on the same instrument, with temperature maintained using a thermostatic bath equipped with refrigeration. Prior to measurements, samples were degassed under vacuum (10^{-5} bar) at 90 °C for 12 h. Thermogravimetric analysis (TGA) was performed on a TA Instruments Trios V5 system under a nitrogen atmosphere at a heating rate of 10 °C min^{-1} from room temperature to 800 °C. Powder X-ray diffraction (PXRD) patterns were recorded on a Bruker D8 Advance diffractometer equipped with a Sol-X energy-dispersive detector. High-resolution scanning electron microscopy (SEM) images were obtained using a Cryo focused ion beam-scanning electron microscopy (FIB-SEM) system to analyze the morphology of SP-UiO samples.

1.3.3 SP content characterization

The molar ratio of Zr and H_2BPDC ratio was calculated by the TGA from air. The molar ratio of H_2BPDC and SP were determined by ^{1}HNMR after digestion. The digested 2 mg SP-UiO samples were transferred to a 20-mL vial and treated with 50.0 µL of D_2SO_4. The resulting solution was then diluted with 500 µL of DMSO-d_6. The samples were then sonicated for 10 minutes to ensure complete dissolution before being transferred to NMR tubes for analysis. Based on the TGA results in air, the molar ratio of BPDC and Zr can be obtained. From the ^{1}H NMR spectrum, the ratio of the integrated proton signals corresponding to BPDC also can be calculated. combining this ratio and ratio of BPDC and Zr, the SP content in SP-UiOs were calculated.

1.3.4 Ideal adsorbed solution theory (IAST) CO_2/N_2 selectivity calculation

The IAST CO_2/N_2 selectivity of MOFs evaluated in this work were determined using the pure-component isotherms of CO_2 and N_2 that were fitted using single-site Langmuir-Freundlich model for full range of pressure (0–1.0 bar).

$$(1)\quad q = q_{\text{sat}} \frac{b_1 p^{v1}}{1 + b_1 \mathrm{p}^{v1}}$$

here, p is the pressure of the bulk gas at equilibrium with the adsorbed phase (bar), q is the adsorbed amount per mass of adsorbent (mmol g^{-1}), qsat is the saturation capacities (mmol g^{-1}), b is the affinity coefficient (bar^{-1}), and v represent the deviation from an ideal homogeneous surface.

$$(2)\ S = \frac{x_1/y_1}{x_2/y_2}$$

The selectivity of the preferential adsorption of component 1 over component 2 in a mixture containing 1 and 2 can be formally defined In the above equation, x_1 and y_1 (x_2 and y_2) are the molar fractions of component 1 (component 2) in the adsorbed and bulk phases, respectively. We calculated the values of x_1 and x_2 using IAST of Myers and Prausnitz.[1]

1.3.5 Photoisomerization of SP-UiOs

The photoswitching behavior of SP-UiO samples was evaluated by UV–vis DRS. Samples were finely ground and gently pressed onto the surface of a $BaSO_4$ substrate to ensure uniform coverage. The samples were then irradiated with UV light (typically for several seconds), during which a noticeable color change was observed, indicating the trans-to-cis isomerization of the SP moieties. DRS spectra were recorded before and after UV exposure to monitor the spectral changes associated with the isomerization process. Subsequently, the irradiated samples were exposed to visible light from a torch (for several minutes) to induce the reverse cis-to-trans isomerization, and DRS spectra were recorded again to evaluate the reversibility of the photoisomerization.

To investigate the effect of photoisomerization on porosity, nitrogen adsorption–desorption isotherms were measured before and after UV irradiation. SP-UiO samples were first degassed under vacuum at 90 °C overnight, followed by measurement of the N_2 isotherms at 77 K using a Quadrasorb Evo system (Quantachrome). After the initial analysis, the same sample cell was sealed with parafilm and uniformly irradiated with UV light inside a light box for 1 hours to ensure sufficient exposure while avoiding contamination or desorption. The irradiated samples were then analyzed immediately under the same isothermal conditions without additional heating to capture the isomerization-induced changes in surface area and pore size distribution. To assess the reversibility of the porosity change, the UV-treated samples were subsequently re-degassed under vacuum at 100 °C overnight and reanalyzed under the same conditions. This cycle confirmed the reversible nature of the isomerization process and its impact on the porous structure of the SP-UiO materials

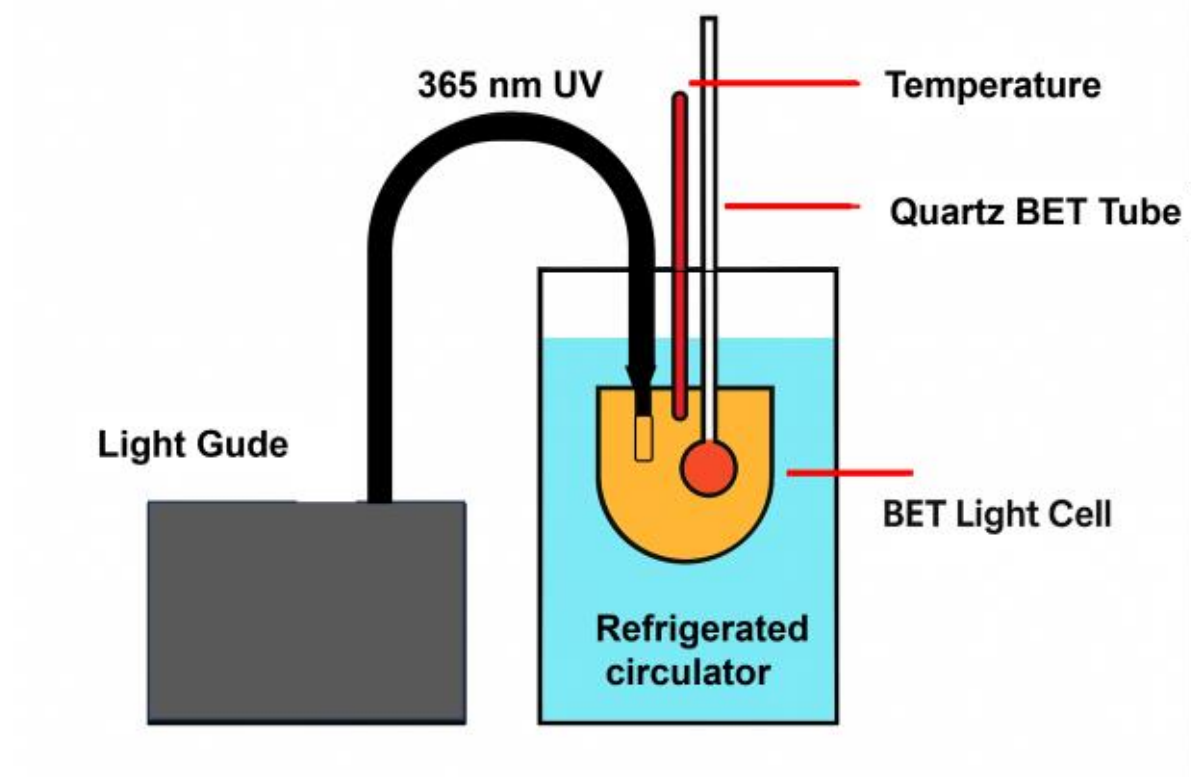

Figure S1:Schematic representation of the experimental setup.

1.3.6 In-situ Light-Responsive CO_2 Adsorption Experiments

A custom-built BET light cell was employed to house the sample tube along with an optical fiber light guide, ensuring uniform and maximum light exposure to the sample during irradiation.[2] A Julabo refrigerated circulator was used to maintain the temperature at either 273 K or 298 K throughout the experiment. Prior to measurement, SP-UiO samples were degassed under vacuum at 90 °C overnight. CO_2 adsorption–desorption isotherms were then recorded using a Quadrasorb Evo (Quantachrome) instrument at 273 K and 298 K to establish the baseline adsorption properties in the dark. Following this, the samples were irradiated with UV light (365 nm) for several minutes and subjected to in-situ CO_2 adsorption analysis under continuous 365 nm UV irradiation to evaluate the light-induced switching behavior. During the measurement, the bath temperature was always monitered by the insterted thermometer. After the measurement, the sample cell was sealed with parafilm and exposed to visible light (white light-emitting diode) in a light box for several hours to ensure uniform irradiation and promote the reverse isomerization. The CO_2 isotherms were remeasured under identical conditions to assess the reversibility of the photoresponsive adsorption behavior. This UV/vis light-switching cycle was repeated three times to evaluate the cycling stability and reproducibility of the photoisomerization-induced CO_2 uptake modulation.

1.3.7 CO_2 breakthrough tests as a function of photo-irradiation

400 mg P-OMe-UiO was put into the quaze container as column. A simulated flue gas mixture containing 20 vol% CO_2 in N_2, was passed through a quartz tube packed with P-OMe-UiO, and the outlet CO_2 concentration was monitored over time through the whole process. Figure S2 shows a schematic of this setup.

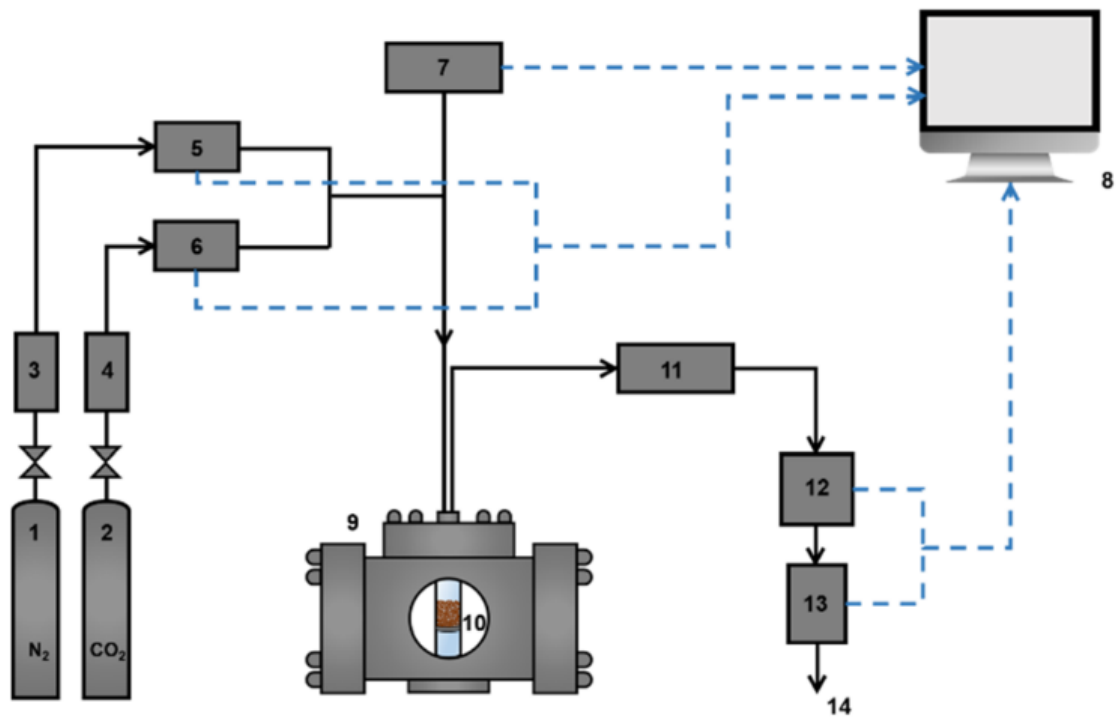

Figure S2: Experimental configuration for photo-modulated desorption: (1) N_2 gas cylinder and regular, (2) CO_2 gas cylinder and regular, (3) zeolite/silica drying column of N_2 gas, (4) zeolite/silica drying column of CO_2 gas, (5) mass flow controller of N_2, (6) mass flow controller of CO_2, (7) fibre-optic temperature sensor and signal conditioner, (8) data analysis workstation, (9) metal retainer, (10) quartz container, (11) water condenser, (12) non-dispersive infrared CO_2 sensor, (13) mass flow meter and (14) gas exit.[3]

1.3.8 X-ray Photo-electron spectroscopy (XPS)

1.3.8.1 In-situ XPS

Photoelectrons were collected and energy analysed using a SPECS Phoibos 100 analyser with a 2D detector totalling over 1000 channels. A PreVac twin anode X-ray flood source providing Al Kα un-monochromated x-Rays at a 54° angle was used, at a base pressure of $5x10^{-10}$ mbar. The data was collected using a 3x7 mm slit, 25 channels, and a 5 mm IRIS to ensure the information collected was purely from the sample area upon which the UIO samples were spread. Core level spectra were taken using a pass energy of 20 eV. For Real-time measurements, 500 channels on the detector were used to obtain the Zr core level, with the detector in snapshot mode, and a pass energy of 60 eV allowing for measurements across a 10 eV window with a 0.02 eV step size. This allowed for the entirety of the core level to be measured in real-time. 3 snapshots were taken every 6 seconds and averaged for each cycle accumulating to 20 seconds total, where 50 cycles of snapshots were obtained for each set of core level spectra recorded. The sample was then irradiated with UV and visible light at different stages of the cycle to observe shifts in kinetic energy.

A Bentham TLS120Xe tunable light source was used for administering the UV and white light onto the sample, going in via windows on both XPS systems with thor-labs focusing optics used on a CF70 port.

1.3.8.2 Near-ambient-pressure XPS (NAP-XPS)

The NAP-XPS was conducted using a 150 1DLD NAP-XPS Specs Phoibos analyser, using a SPECS NAP-XPS Monochromated Al Kα X-ray source at an angle of 54°. The nitrogen and carbon dioxide gases were >99.9999% purity and were pre-mixed into there correct ratios before being administered into the analysis chamber via a needle valve.

NAP-XPS and light measurements were conducted using the tuneable light source as mentioned

above. The chamber was backfilled to the correct pressure by removing all acting pumping from the analysis chamber and then flowing in the gas mixture. Core level spectra were measured at 20eV pass energy.

The real-time XPS is conducted with a custom, real-time detector in a snapshot mode across a 10 eV window, with a step size resolution of 0.02 eV. This allows us to accurately obtain information across the core levels quickly, which has a much greater level of accuracy than is typical of XPS, and other real-time XPS methods for measuring the exact shifts. The shifts observed in the XPS before and after UV exposure in the high-resolution core level spectra match the data provided by the in-situ real-time exposure. For the P-OMe-UiO sample, the shift after exposure to UV is approximately 0.07 eV, which matches the value seen in the core level spectra. The average error in the real-time spectra due to noise is ± 0.02 eV. Samples measured in ultra-high vacuum (UHV) before and after UV exposure, and during real-time measurements, remain stationary and in position to ensure high accuracy. Sample measurements are repeated and show the same effect.

As shown in S. Astley et al.4, binding energy shifts of small values can be detected as low as the values we observe and can arise due to a variety of factors. Importantly, even when thermally annealing the sample up to 600°C with gold alloys, shifts of only 0.06-0.07 eV are observed, demonstrating the instrument's sensitivity at this scale. The power of the tunable light source at the UV wavelength used was measured at 1.65 µW at the sample. At such low powers, shifting due to thermal effects is undetectable. Sample temperatures were monitored continuously during experiments via direct contact measurement of the mounting plates, and no thermal change was observed (within ±0.1°C accuracy) before, during, or after UV exposure. The white light source is considerably more powerful at 0.1 mW, yet the Zr 3*d* spectra return to the exact same energy as pre-UV conditions during white light illumination, and no temperature change was observed. This indicates that any shifts are entirely due to the molecular transition rather than thermal or charging effects.

Samples are mounted onto a grounded conductive plate to compensate for any intrinsic charging. A twin-anode flood source X-ray gun was used during measurements in the UHV system; these typically generate a large number of stray electrons which suppress potential charging effects in XPS. In cases where charging has been observed, peaks have been shifted with respect to charge referencing of the C 1*s* spectrum. If charging were the cause of the observed shifts, the O 1*s* would shift in the same direction as the Zr peak, and the peaks would not alternate between higher and lower kinetic energies. Charging would also be expected to produce effects across the full range of samples rather than specifically in P-OMe-UiO.

Astley S, et al. Identifying chemical and physical changes in wide-gap semiconductors using real-time and near ambient-pressure XPS. Faraday Discuss 236, 191-204 (2022).

1.3.9 Ion adsorption and desorption experiment

1000 ppm and 500 ppm NaCl salt solution were prepared for the measurement of ion adsorption loading. 10 mg of P-UiO or P-OMe-UiO and 10.00 ml of salt solution were put in a 10 ml vial. The adsorption occurred under UV light and 2 hours stirring. After the adsorption of the salt, the adsorbent suspension was centrifuged, and the supernatant was filtered for inductively coupled plasma optical emission spectroscopy (ICP-OES) measurement. After centrifuge, the desorption was carried out under 1 hours white light stirring in deolized water solution. The released metal ion concentration was then analyzes by ICP-OES.

1.3.10 the isosteric enthalpy of adsorption (Q_{st})

The isosteric enthalpy of adsorption (Q_{st}) for CO_2 was determined using the Clausius-Clapeyron analysis. CO_2 adsorption isotherms were collected at 273 K and 298 K for all SP-UiO samples, both prior to and following UV irradiation. At each loading point, the isosteric heat was calculated according to the Clausius-Clapeyron equation:

$$(3)\ Q_{st}=-R\frac{\partial \ln P}{\partial (1/T)}\Big|_{n}$$

where R is the universal gas constant (8.314 J mol^{-1} K^{-1}), P is the equilibrium pressure at a given adsorbed amount n, and T is the absolute temperature. Adsorption isotherms were fitted using a dual-site Langmuir model prior to Q_{st} calculation to enable interpolation at consistent loading points across both temperatures.

1.4 Computational and molecular simulations

1.4.1 Models

The initial crystallographic information for the UiO-67 structure was obtained from the available literature.[4] Based on experimental observations reported in this work, the UiO-67 structure is found to contain seven linkers per metal oxide cluster. A defective model with approximately seven linkers per metal oxide cluster was constructed within the unit cell of UiO-67 using the Materials Studio Visualizer software.[5] The model was then optimized using the FORCITE module, employing forcefield parameters derived from the Universal Force Field (UFF[6]) and charge-equilibration (Q_{eq}) charges assigned to the framework atoms. Similarly, models for other spiropyran-substituted UiO-67 structures were generated by replacing selected BDC linkers with functionalized spiropyran (the number of linkers replaced corresponds to experimentally observed data).

1.4.2 Density Functional Theory Calculations

Periodic geometric optimization and static binding energies for each CO_2 molecule in the UiO-67 and functionalized structures were calculated using density functional theory (DFT) as implemented in the Vienna Ab initio Simulation Package (VASP) 5.4.4. It is well-known that standard DFT methods, based on the generalized gradient approximation (GGA), do not fully capture the long-range dispersion interactions between the framework and the adsorbed molecules. To accurately estimate the static binding energies of each CO_2 molecule with the framework, dispersion corrections were included using the DFT-D3 method.[7] Electron exchange and correlation were described using the Perdew-Burke-Ernzerhof (PBE) GGA functional[8], and projector-augmented wave (PAW) potentials[9] were employed to treat core and valence electrons. A plane-wave kinetic energy cutoff of 520 eV was used, which was chosen based on the system size, along with a Gamma-point mesh (1 × 1 × 1) for Brillouin zone sampling. The ionic coordinates were fully relaxed until the Hellmann-Feynman forces on the ions were less than 0.02 eV/Å.

1.4.3 Binding Energy Calculations

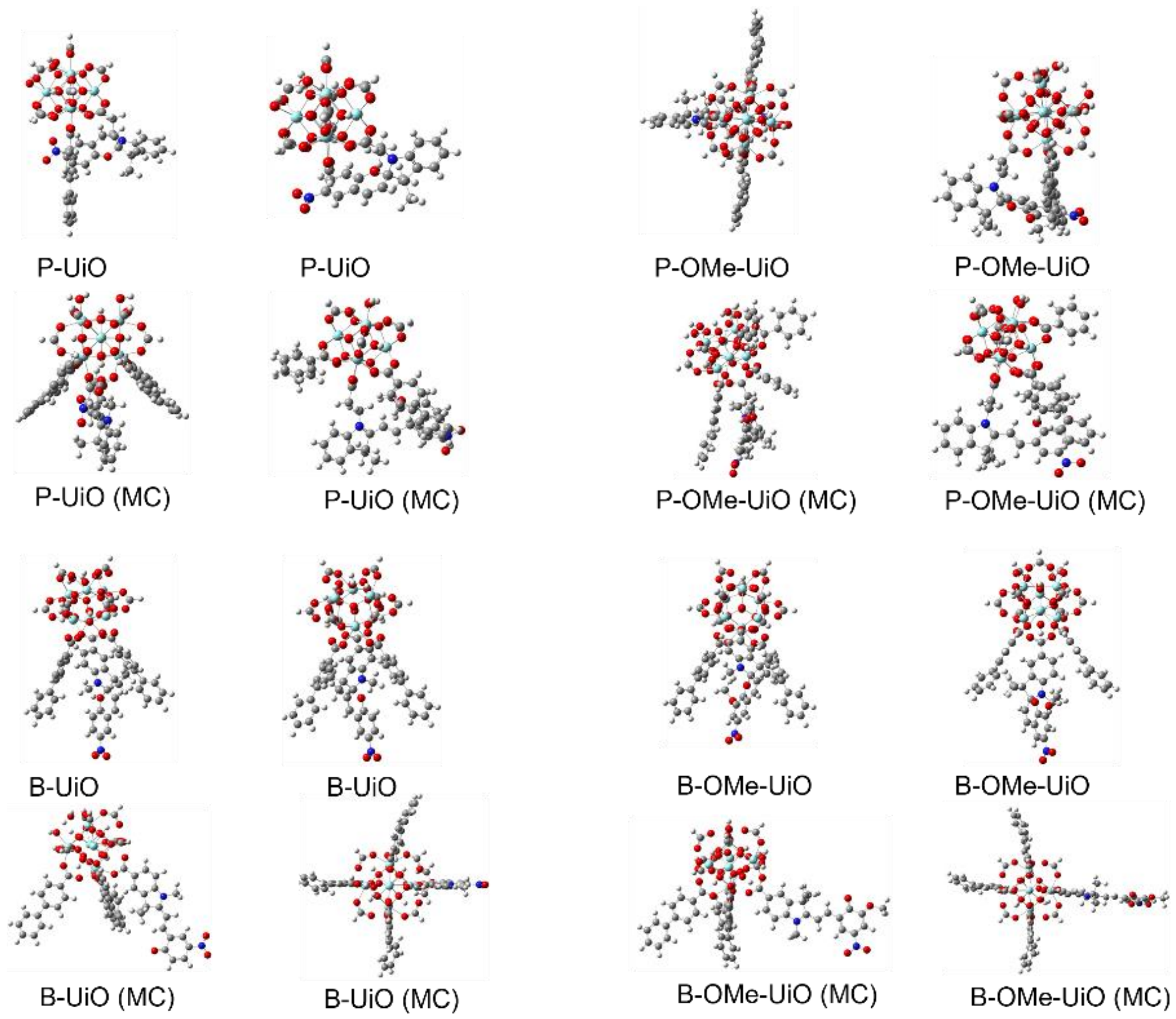

Figure S3. Modelling secondary building unit (SBU) structures of different SP-UiO and their merocyanine (MC) isomers for CO_2 binding energy calculation.

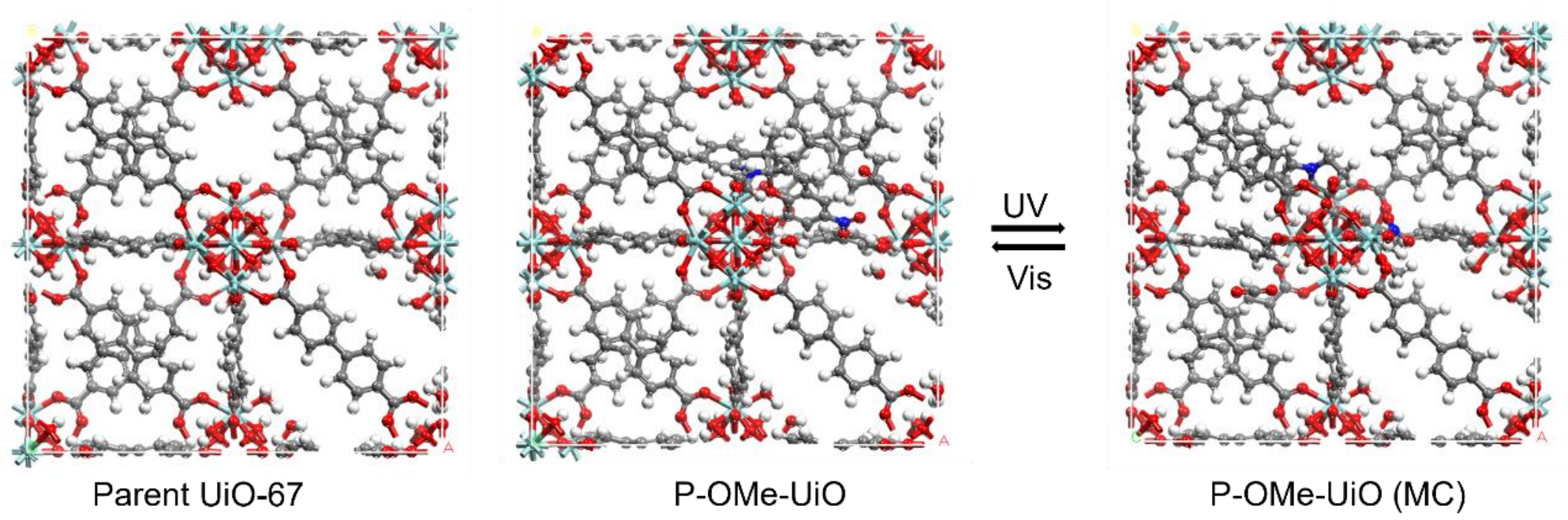

Figure S4. periodic modelling structures of parent UiO-67, P-OMe-UiO and MCOMeCO-UiO for CO_2 binding energy calculation. A defect-free UiO-67 is theoretically composed of 12-linker-coordinated metal nodes. However, the defect-engineered UiO-67 synthesized in this study exhibits metal nodes coordinated by seven linkers, as illustrated in the simulated parent UiO-67 model. After open ring isomerization of P-OMe-UiO, the the phenolic oxygen complexed with the near defective metal nodes and then reduce the amount of open Lewis acid centers available for interactions with CO_2

The initial positioning of CO_2 molecules in the UiO-67 and functionalized structures was obtained using a classical simulated annealing technique based on a classical force field, as implemented in the Sorption module of Materials Studio[5]. During the simulation, the framework atoms were kept fixed, and a single gas molecule was placed inside the simulation box, which consisted of multiple unit cells. The dimensions of the box were chosen such that each direction was at least twice the cutoff radius. The interactions between the gas molecules and the MOF framework were modelled using a combination of pairwise site-site Lennard-Jones (LJ) and Coulombic potentials. The LJ parameters for both the framework atoms and the gas molecules were derived from the Universal Force Field (UFF).

In the simulated annealing process, the temperature was gradually decreased, allowing the gas molecule to reach an optimal configuration. This was achieved through configurationally biased constant-number, constant-volume, constant-temperature (NVT) Monte Carlo moves, including rotation, translation, and repositioning, with preset probabilities for each move. The system underwent multiple heating and cooling cycles to find the local energy minimum. A total of 40 heating cycles were performed, with a maximum temperature of 105 K and a final temperature of 100 K. The lowest-energy configuration obtained from the simulated annealing process was chosen as the initial configuration for subsequent DFT binding energy calculations.

Static interaction energies (ΔE) at 0 K in vacuum were calculated using the following expression:

$$(4)\ \ \Delta E = E_{MOF+gas} - E_{MOF} - E_{gas}$$

where E_X refers, respectively, to the total energies of the MOF + gas complex, the MOF alone, and the gas.

1.4.4 UV-Vis absorption spectra calculations

The ground-state geometry of the MOF cluster cleaved from the unit cell was optimized using the M06-2X functional with the 6-311G++(d,p) basis set and the solvation model based on density (SMD) implicit solvent model (DMSO as solvent) using the Gaussian 09 suite of program[10]. The UV–visible absorption properties were then evaluated using time-dependent DFT (TD-DFT) at the same level of theory.

Then, CO_2 was adsorbed on MOF cluster and binding energy with MOF cluster was calculated using the formula:

$$(5)\ BE = E_{MOF+CO2} - E_{MOF} - E_{CO2}$$

Where BE is Biding Energy of CO_2, E_X refers, respectively, to the total energies of the MOF + CO_2 complex, the MOF alone, and the CO_2.

1.4.5. Pore size distribution

The pore size distribution in spiropyran-functionalized UiO-67 and its MC isomers was calculated using a probe radius equivalent to the kinetic diameter of nitrogen gas, using the PoreBlazer tool[11].

1.5 Life-cycle assessment (LCA) methodology

1.5.1 Goal and Scope

The objective of this life-cycle assessment is to evaluate and compare the environmental impacts associated with the operational energy consumption of three adsorption-based CO_2 capture technologies including light-induced swing adsorption (LISA), temperature-swing adsorption (TSA), and pressure-swing adsorption (PSA), for the removal of CO_2 using 1 g of adsorbent. SPOMeCO-UiO is employed as the adsorbent in the LISA system, while UiO-67 is utilized for both TSA and PSA configurations[12]. To ensure comparability across technologies, the functional unit (FU) is defined as 1 g of adsorbent required to capture 1 mol of CO_2. This study focuses exclusively on the operation and regeneration energy necessary to restore the adsorbent to working condition after each cycle. Upstream processes, including adsorbent synthesis, equipment manufacturing, infrastructure construction, and end-of-life treatment, are excluded from the base-case analysis unless otherwise specified.

A cradle-to-gate framework is adopted for this LCA study. However, the system boundary is intentionally narrowed to encompass only the operational phase of the adsorption systems. Accordingly, only the energy flows directly associated with cyclic adsorption-desorption and adsorbent regeneration are included in the environmental inventory.

The system boundary was limited to the operational phase, specifically the energy consumption associated with adsorption-desorption cycling and adsorbent regeneration, while upstream and downstream processes such as adsorbent synthesis, equipment fabrication, infrastructure, and end-of-life treatment were excluded. Key assumptions include:

1. Adsorbent materials (P-OMe-UiO and UiO-67) were assumed to maintain structural integrity and adsorption capacity during repeated adsorption-desorption cycles, with no loss of activity, material degradation, or replacement requirement considered.
2. All energy demands were modelled using the United Kingdom electricity grid mix available in the SimaPro database, assuming a constant grid composition throughout the assessment period.
3. Thermal regeneration in the TSA process was assumed to be supplied entirely through electric heating to maintain methodological consistency and comparability among the LISA, TSA, and PSA systems.
4. Ideal energy transfer was assumed during regeneration processes, where no additional energy losses from auxiliary equipment, heat dissipation, or system inefficiencies were considered unless explicitly reported in the source data.
5. Operational energy demand was assumed to be the dominant contributor within the defined system boundary.

Nevertheless, several limitations should be acknowledged. The present assessment focused solely on operational energy consumption and therefore did not capture potential environmental burdens associated with adsorbent synthesis, solvent usage, material replacement, or infrastructure requirements. Furthermore, the assumption of constant adsorbent performance neglected possible capacity decay and regeneration inefficiencies during long-term operation.

1.5.2 LCA Inventory

Working capacities and per-cycle energy demands for each adsorption process were obtained from the experimental dataset and published research[12], with a summary provided in Table S4. These parameters were used to determine the number of adsorption-desorption cycles required

to achieve the defined FU and to quantify the corresponding total energy demand. Consistent with the scope definition, upstream burdens such as adsorbent production, equipment fabrication, and plant construction were excluded from the inventory.
Conversion of cycles and total energy per FU:

$$(6)\ \text{cycles} = \frac{1000\ \text{mmol}\ (1\ \text{mol}\ CO_2)}{\text{working capacity}\ (\text{mmol}\ g^{-1})}$$

$$(7)\ E_{total,FU} = E_{cycle} \times \text{cycles}$$

Life cycle inventory modelling was conducted using SimaPro v9.0, employing the United Kingdom electricity grid mix as the energy supply for all processes. For TSA, thermal regeneration was assumed to be supplied through electrical heating to maintain methodological consistency among the three adsorption routes. The total regeneration energy per functional unit was calculated by multiplying the per-cycle energy requirement by the number of cycles needed to capture 1 mol of CO_2. Environmental impacts were quantified using the ReCiPe 2016 Midpoint (H) method, with characterization factors and background datasets applied as provided in the default LCA database without modification.

2. Supplementary Figures and Tables

2.1 Physicochemical characterization of SP-UiO MOFs

2.1.1 FTIR spectra of SP-UiO MOFs

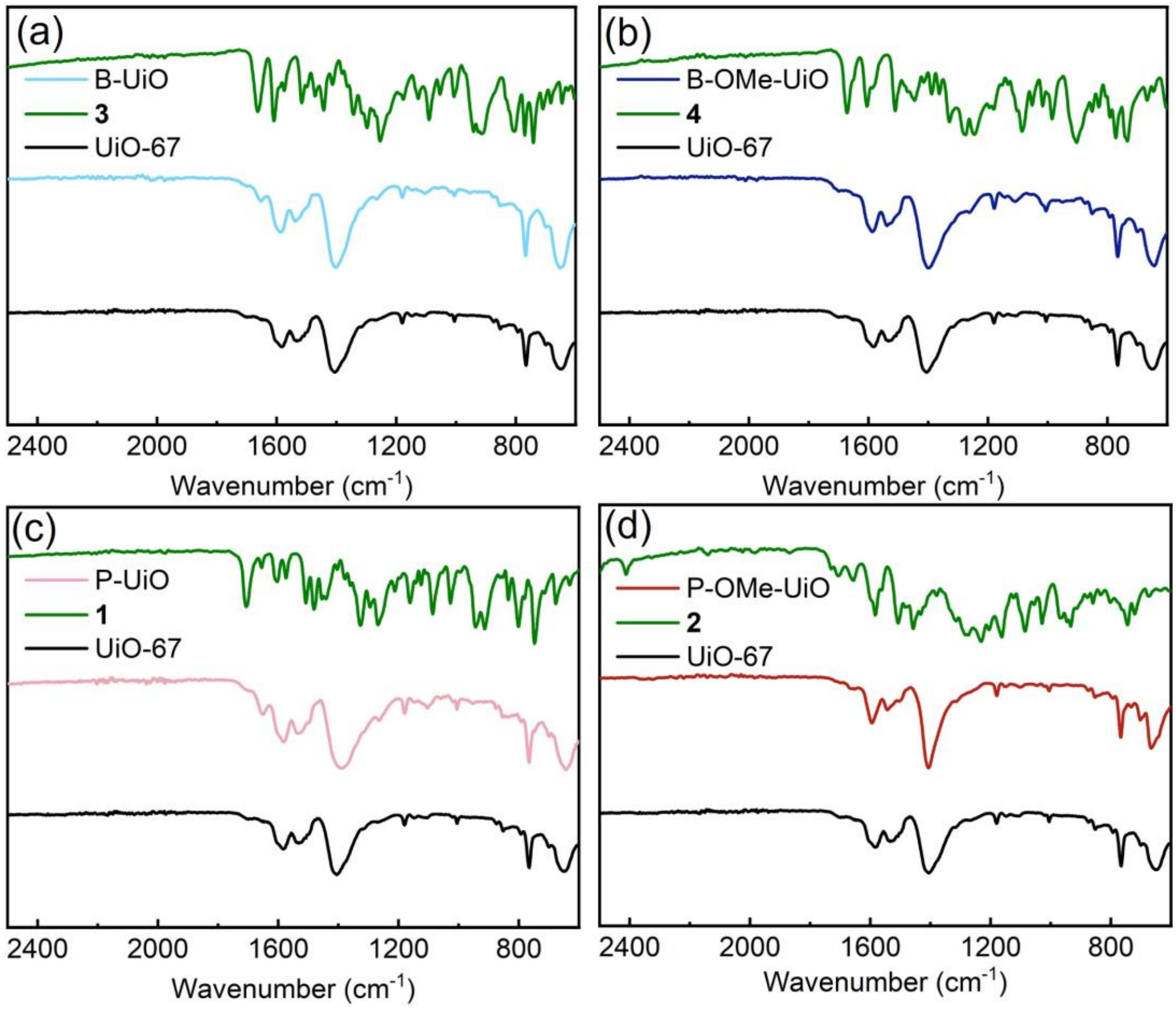

Figure S5. FTIR spectra of defective UiO-67, (a) **3** and related B-UiO; (b) 4 and related B-OMe-UiO; (c) **1** and related P-UiO; and (d) **2** and related P-OMe-UiO. The characteristic peaks at 655 and 775 cm^{-1} in UiO-67 correspond to Zr–O bonding vibration. The peak centred at 1398 cm^{-1} is due to C=C bonding vibrations of the benzene ring in a ligand moiety. The peaks at 1545 and 1604 cm^{-1} are due to the coordinated carboxylate groups (COO^-) in the UiO-67. All SP-UiO suggested these characteristic peak from parent UiO-67, Apart from that, we also observed peaks characteristic of non-aromatic C=C stretching vibration and C-O/C-N stretching of SP at approximately 1680 cm^{-1} and 1250 cm^{-1} from related spiropyrans (Fig. 1b).

2.1.2 TGA curves of SP-UiO MOFs

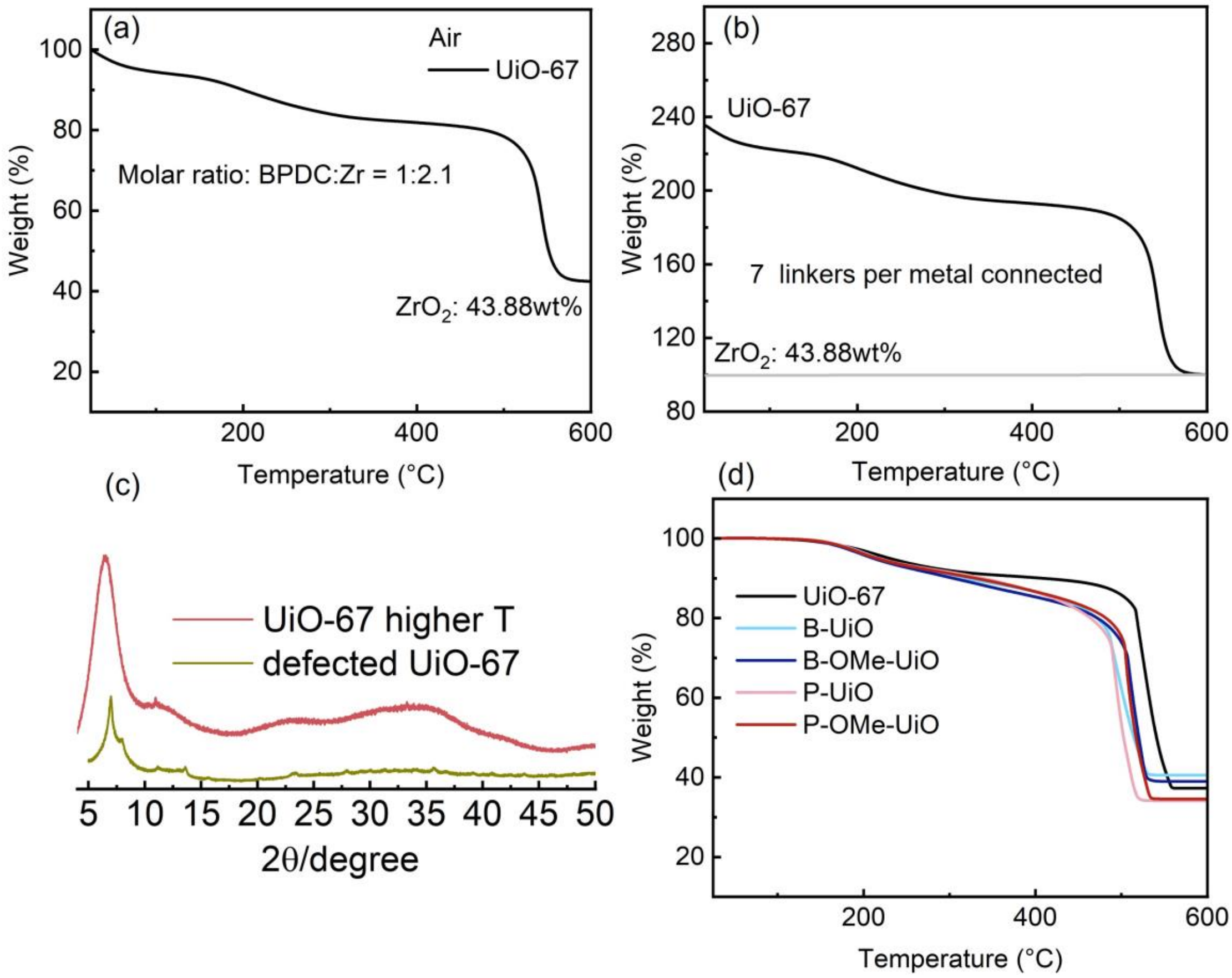

Figure S6. (a) TGA curve of defective UiO-67 under air flow under 10 mL/minute air flow, and temperature ranges up to 600 °C with a heating rate of 5 °C/minute. (b) The TGA data of defective UiO-67 was normalized to 100% at 600 °C, corresponding to ZrO_2. (c) PXRD of defective UiO-67 and UiO-67 fabricated by autoclave. (d) TGA curves of all SP-UiO MOF under N_2 flow under 10 mL/minute N_2 flow, and temperature ranges up to 600 °C with a heating rate of 5 °C/minute.

Thermogravimetric analyses in air indicated that the linker-to-metal node ratio in the defective UiO-67 was approximately 1 linker per 2.1 Zr nodes. Based on MOF weight loss between 300 – 400 °C, we quantified that 7 H_2BPDC linkers were coordinated to each Zr node in these defective UiO-67. Ideally each Zr node in UiO-67 should be coordinated to 12 H_2BPDC linkers.[13] TGA of SP-UiO under N_2 suggested a thermal stability up to 180 °C. A decrease in the thermal stability of SP-UiOs compared to pristine UiO-67 was also observed, which can be attributed to the lower decomposition temperature of the SP moieties relative to the native organic linkers

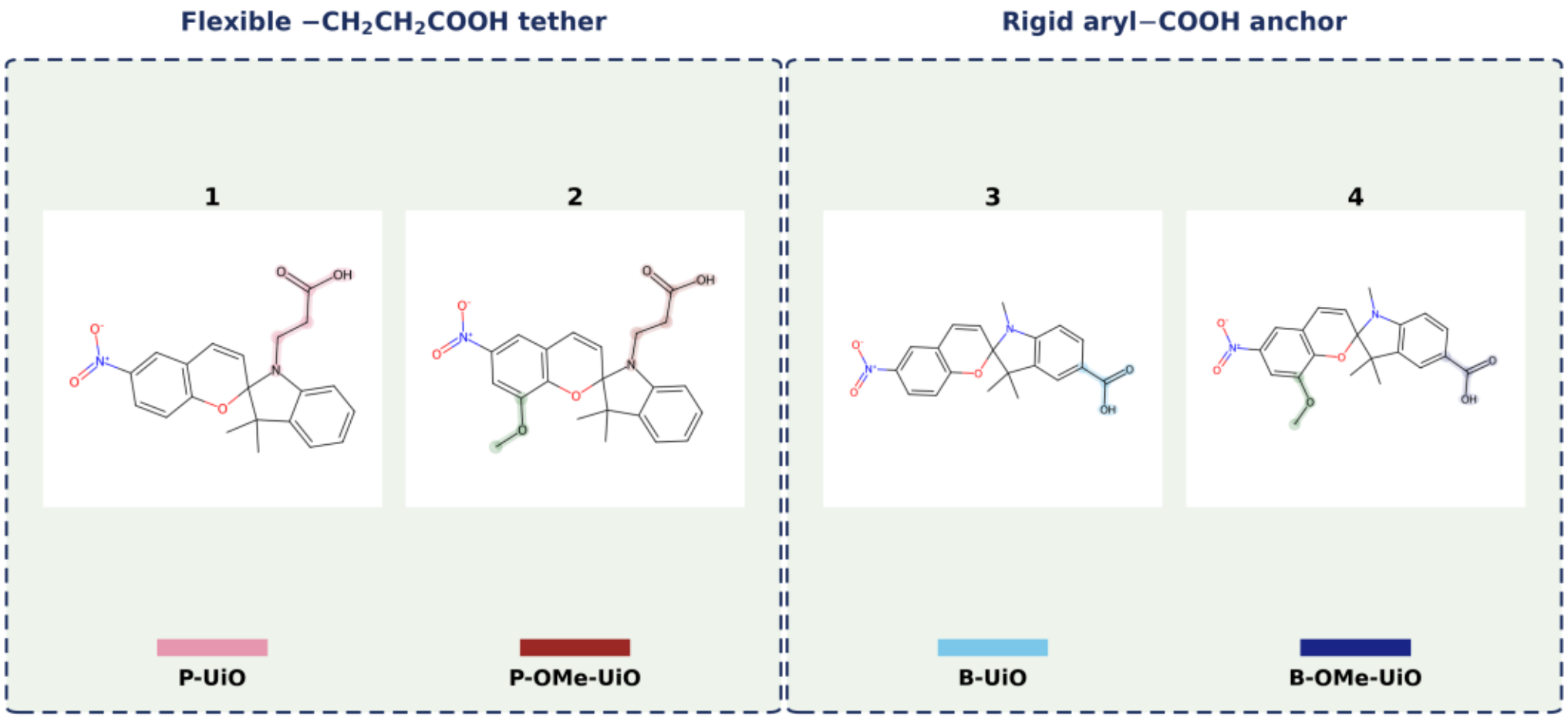

Figure S7. The chemical structures of 1 – 4 and the difference in the carboxyl tether. The flexible alkyl chain in ligands 1 and 2 relieves steric constraints at the grafting site and facilitates ordered coordination onto the Zr nodes. In contrast, the rigid benzene ring in ligands 3 and 4 causes steric congestion at the defect site, restricting ordered assembly and blocking the pore. This is fully consistent with the BET trends: P-series materials show enhanced surface areas (~1620–1632 m^2/g), while B-series materials show a >50% reduction (~765 m^2/g), attributed to SP units occupying pore cavities rather than selectively passivating defect sites.

2.1.3 ^{1}H NMR spectra of digested SP-UiO MOFs

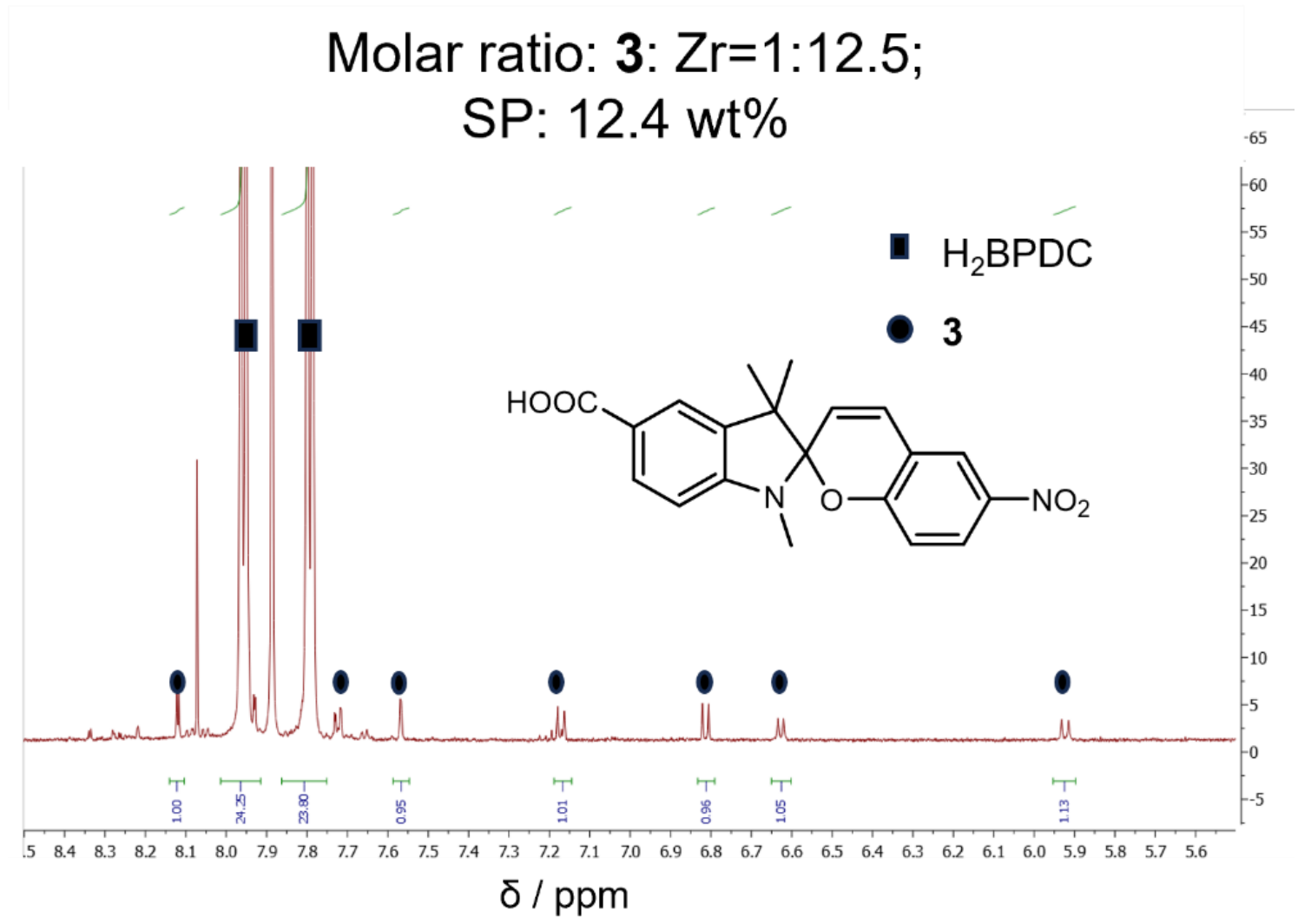

Figure S8. ^{1}H NMR spectra of digested B-UiO. The digested 2 mg B-UiO samples were transferred to a 20-mL vial and treated with 50.0 μL of D_2SO_4. The resulting solution was then

diluted with 500 µL of DMSO-d_6. The samples were then sonicated for 10 minutes to ensure complete dissolution before being transferred to NMR tubes for analysis. Based on the TGA results in air, the molar ratio of BPDC and Zr was 1:2.1. As shown in the ^{1}H NMR spectrum, the ratio of the integrated proton signals corresponding to BPDC and **3** was determined to be 1:12.5. Based on this ratio and ratio of BPDC and Zr, the 3 content in B-UiO was calculated to be 12.4 wt%.

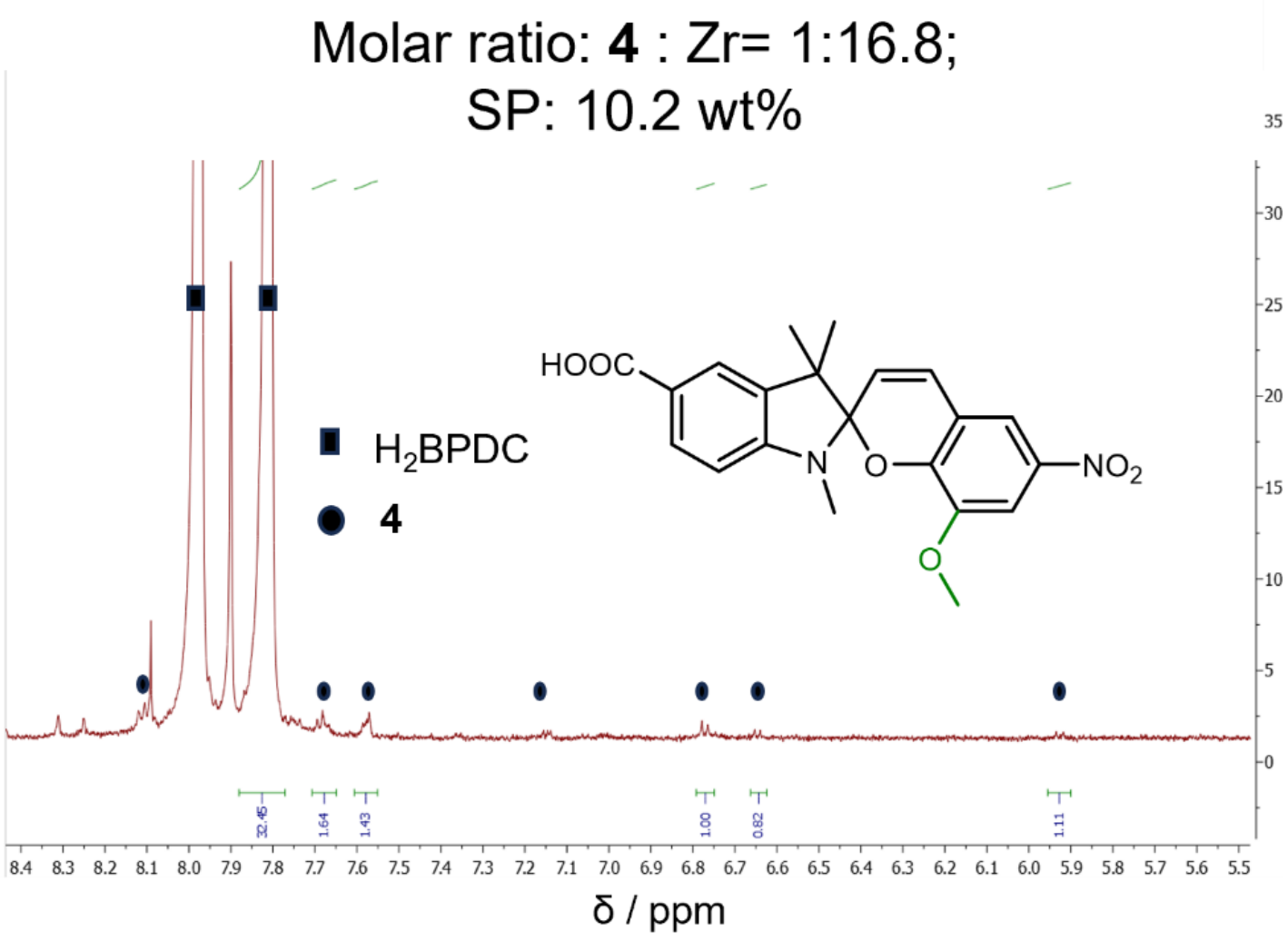

Figure S9. ^{1}H NMR spectra of digested B-OMe-UiO. The digested 2 mg B-OMe-UiO samples were transferred to a 20-mL vial and treated with 50.0 µL of D_2SO_4. The resulting solution was then diluted with 500 µL of DMSO-d_6. The samples were then sonicated for 10 minutes to ensure complete dissolution before being transferred to NMR tubes for analysis. Based on the TGA results in air, the molar ratio of BPDC and Zr was 1:2.1. As shown in the ^{1}H NMR spectrum, the ratio of the integrated proton signals corresponding to BPDC and **4** was determined to be 1:16.8. Based on this ratio and ratio of BPDC and Zr, the 3 content in B-OMe-UiO was calculated to be 10.2 wt%.

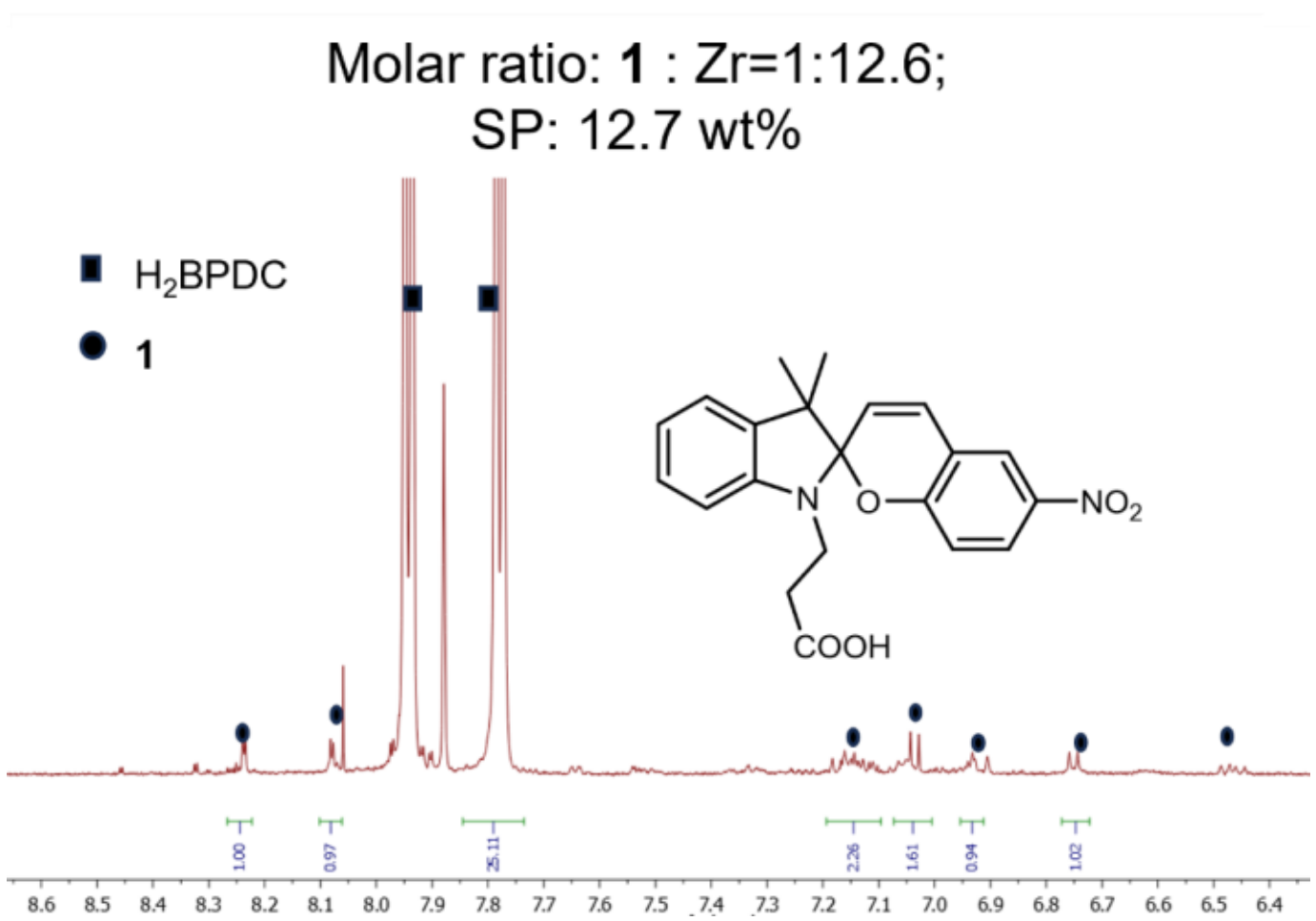

Figure S10. ^{1}H NMR spectra of digested P-UiO. 2 mg P-UiO samples were transferred to a 20-mL vial and treated with 50.0 µL of D_2SO_4. The resulting solution was then diluted with 500 µL of DMSO-d_6. The samples were then sonicated for 10 minutes to ensure complete

dissolution before being transferred to NMR tubes for analysis. As shown in the ^{1}H NMR spectrum, the ratio of the integrated proton signals corresponding to BPDC and **1** was determined to be 1:12.6. Based on this ratio and ratio of BPDC and Zr, the 1 content in P -UiO was calculated to be 12.7 wt%.

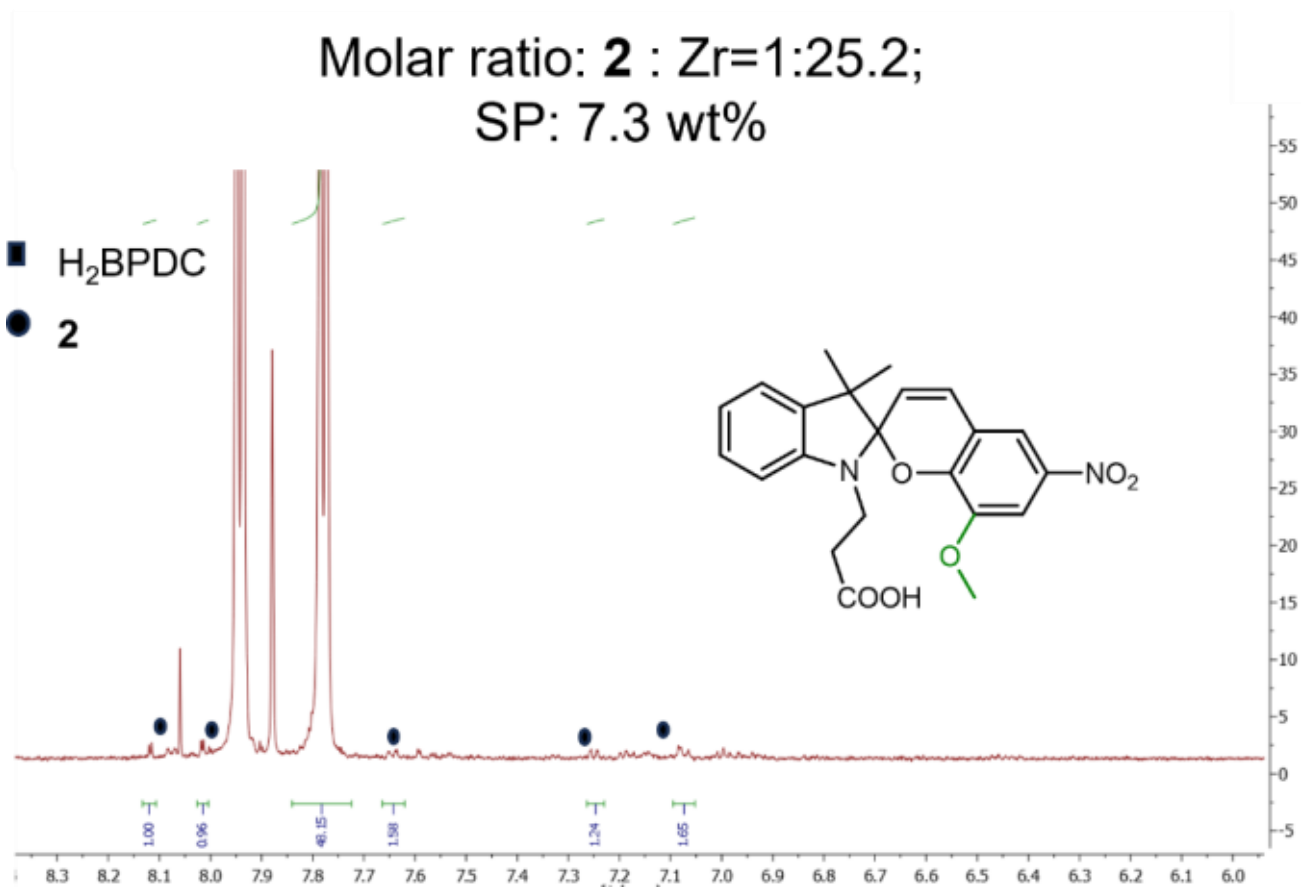

Figure S11. ^{1}H NMR spectra of digested P-OMe-UiO. 2 mg P-OMe-UiO samples were transferred to a 20-mL vial and treated with 50.0 μL of D_2SO_4. The resulting solution was then diluted with 500 μL of DMSO-d_6. The samples were then sonicated for 10 minutes to ensure complete dissolution before being transferred to NMR tubes for analysis. As shown in the ^{1}H NMR spectrum, the ratio of the integrated proton signals corresponding to BPDC and **2** was determined to be 1:25.2. Based on this ratio and ratio of BPDC and Zr, the 2 content in P-OMe-UiO was calculated to be 7.3 wt%.

2.1.4 N_2 adsorption isotherms of SP-UiO MOFs

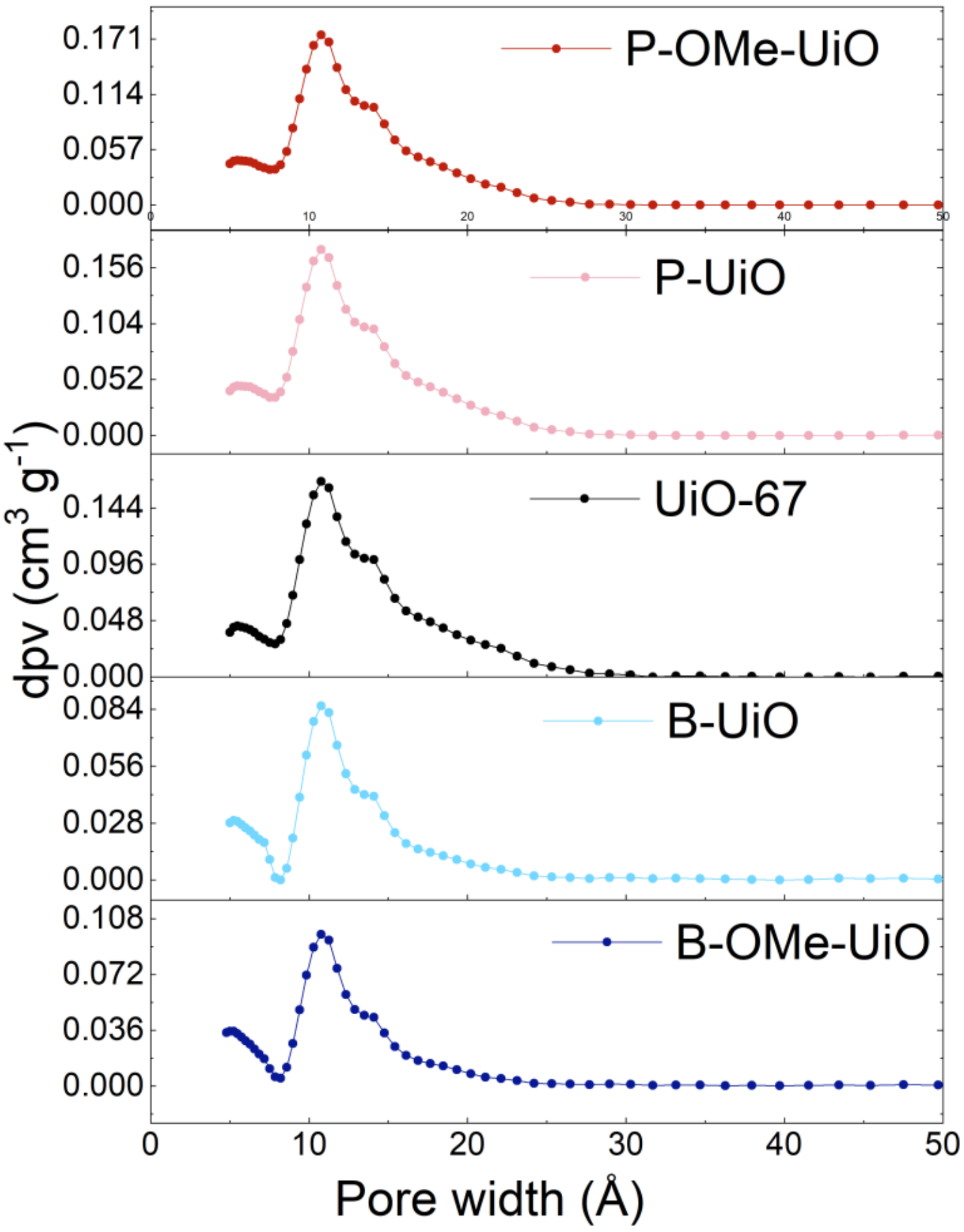

Figure S12. The pore size distribution of SP-UiO MOFs calculated with the NLDFT method using data from Figure 1*d*. The results reveal a predominantly microporous structure with pore widths centered around ~1.2 nm, attributed to the tetrahedral cages formed by the Zr_6 nodes interconnected by BPDC linkers. This is consistent with previous reports showing UiO-67 exhibits type I isotherms and narrow pore size distributions characteristic of microporous frameworks. All SP-functionalized UiOs retain similar pore sizes compared to the parent UiO-67, with variations primarily in pore volume rather than diameter.

2.1.5 SEM-EDX analyses of SP-UiO MOFs

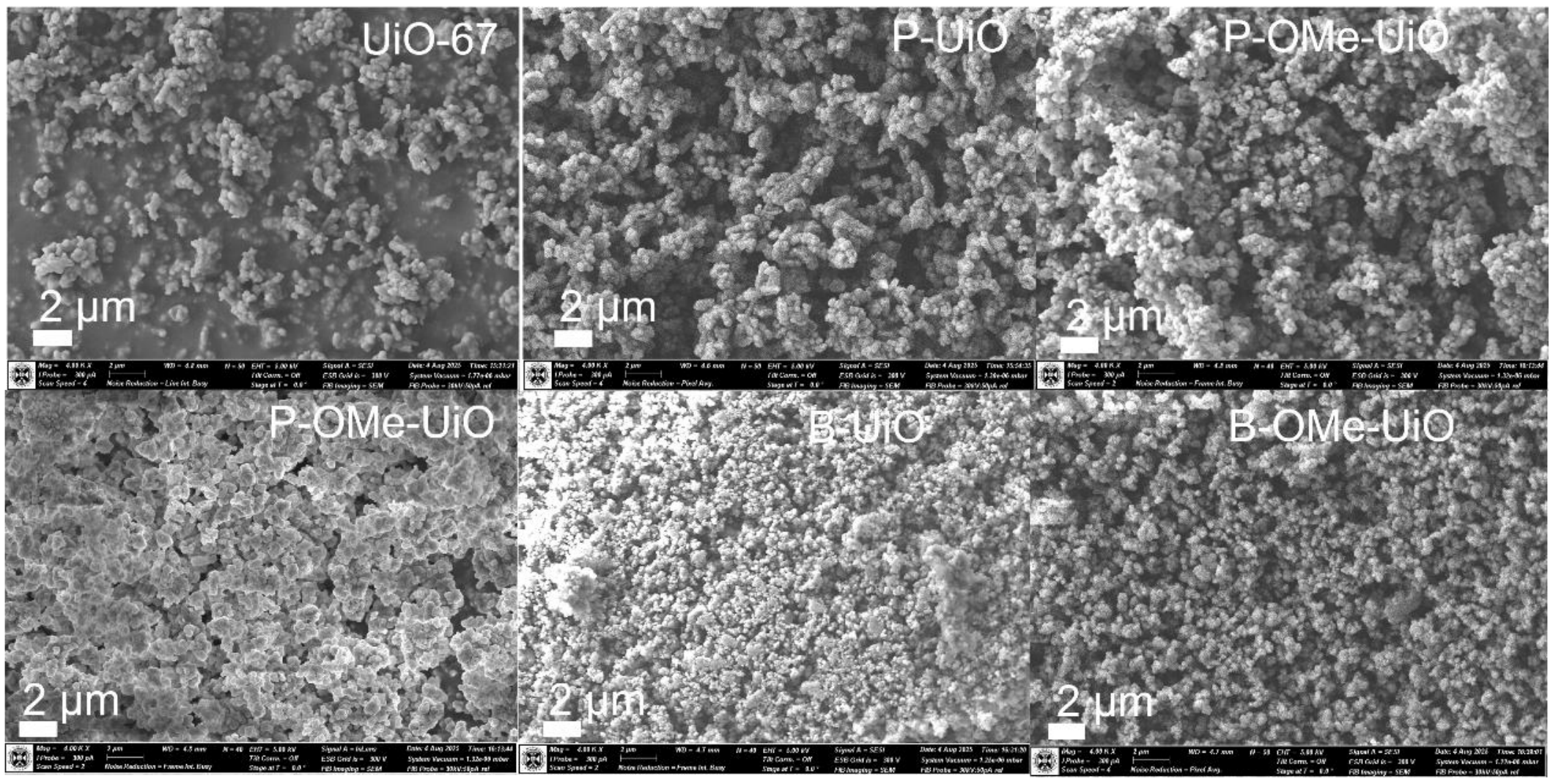

Figure S13. SEM images reveal that SP-UiOs still maintain the same partical mophology as parent UiO-67, exhibiting a uniform morphology of average size smaller than 500 nm.

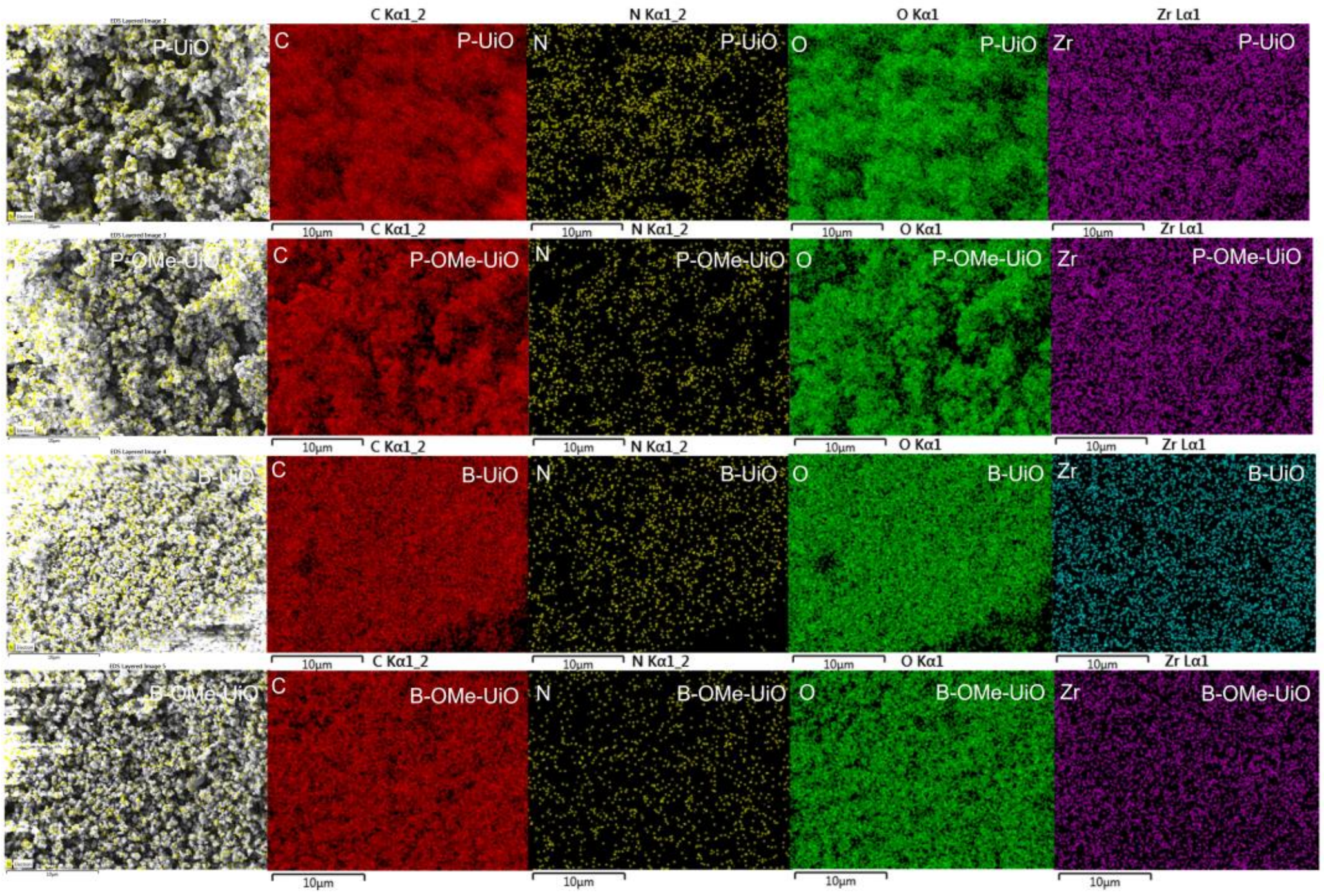

Figure S14. EDX analyses showing C, N, O, Zr mapping of SP-UiOs. The result revealed a relatively uniform distribution of nitrogen across the samples, indicating the uniformly grafting SPs in the parent UiO-67. The results also show the dense distribution of Zr, O and C owning to the high content in parent UiO-67.

2.1.6 DRS analyses of SP-UiO MOFs

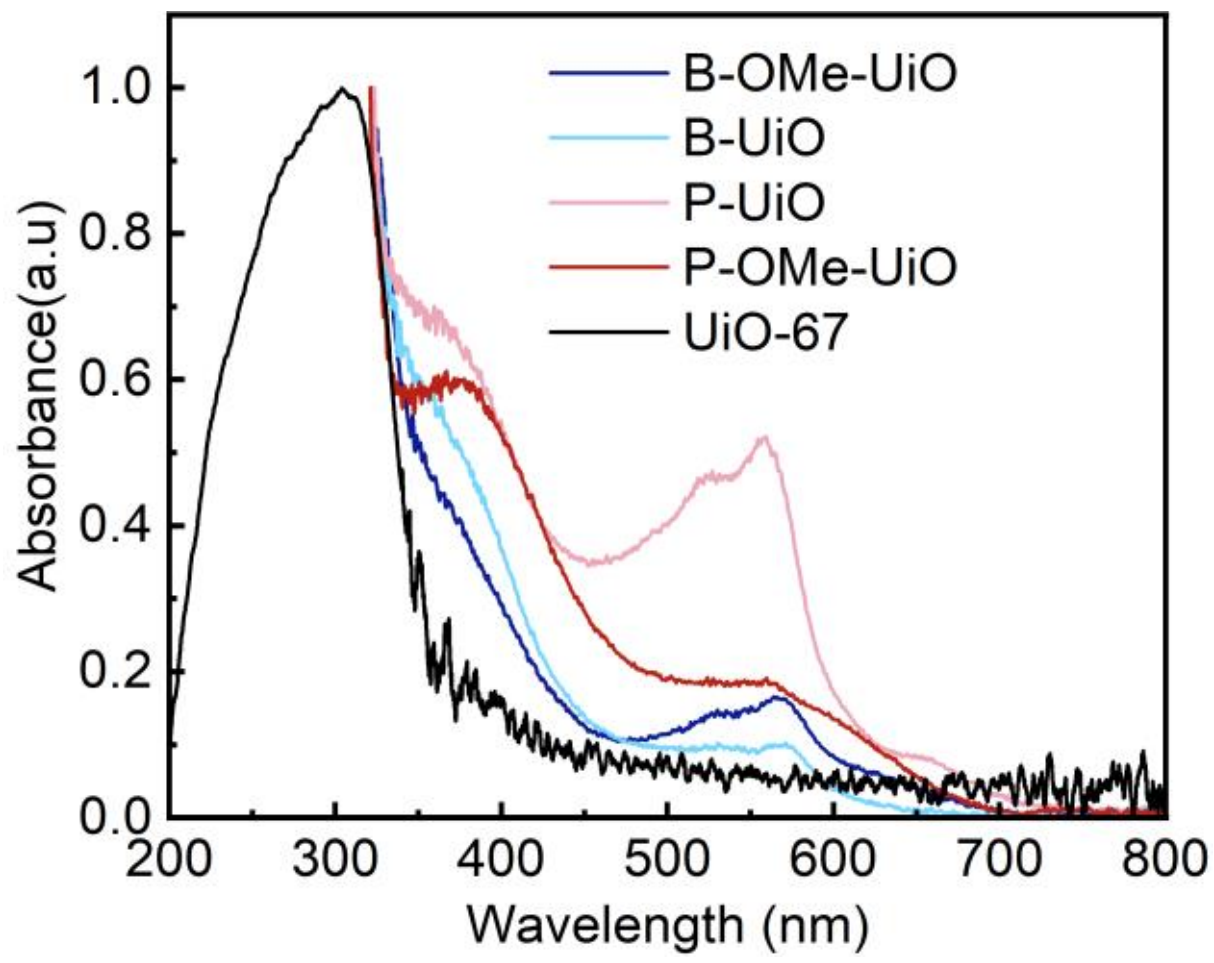

Figure S15. DRS analyses of parent UiO-67 and SP-UiO MOFs studied in this work.

As shown in this figure, the introduction of SP units leads to a pronounced enhancement in visible-light absorption, which can be attributed to the extended π-conjugated structure of SP. This observation is further corroborated by the apparent color change of the samples. Compared with pristine white UiO-67, the emergence of diverse colors in the SP-modified MOFs directly confirms the successful incorporation of SP units into the MOF framework

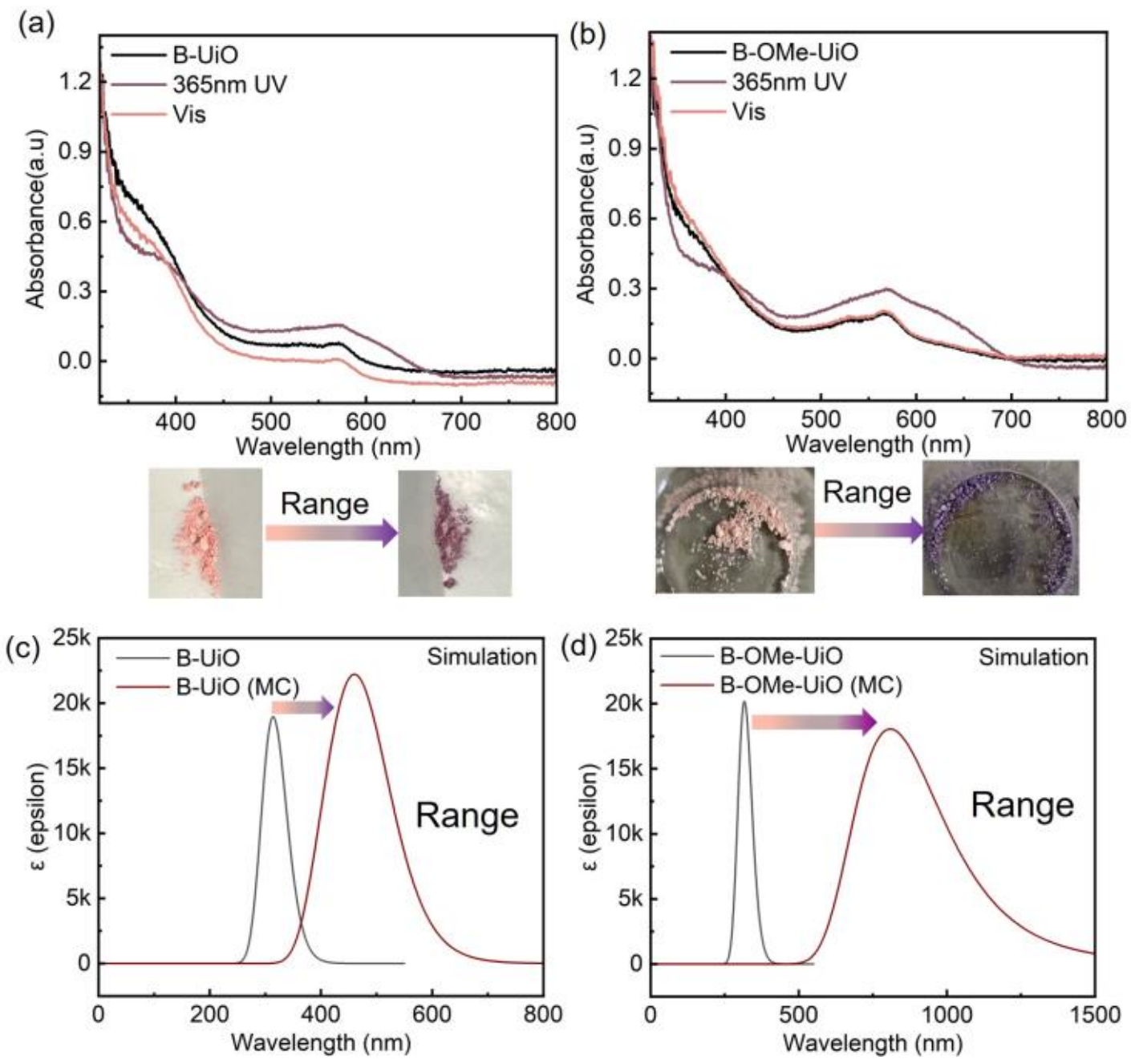

Figure S16. UV–vis DRS of (a) B-UiO (b) B-OMe-UiO before and after UV light irradiation, and lateral visible light irradiation. Simulate the UV–vis absorption spectra of (c) B-UiO, (d) B-OMe-UiO in both SP and MC states.

The SP-UiO MOFs studied here exhibited fully reversible, distinct color changes in the solid state upon exposure to UV (λ = 365 nm) and visible (λ = 400-780 nm) light from off-the-shelf torches. The color changes were triggered immediately upon UV-irradiation of the MOFs. This indicated that SP readily photo-isomerized into its open, zwitterionic MC form in the MOFs. After SP-to-MC photo-isomerization, the colors of P-UiO (light red) darkened. This correlated

to the slight shifts in the absorption edges of P-UiO and their significantly enhanced absorption intensities. Meanwhile, we observed pronounced red-shifts and broadening of the visible absorption range in the absorption spectra of B-UiO and B-OMe-UiO. Hence the pink B-UiO and B-OMe-UiO changed colors to light and dark purple, respectively. All SP-UiO can naturally recover back to SP state under darkness or 5 min visible light irradiation. However, MC-to-SP isomerization in P-UiO and B-UiO was slower than that in P-OMe-UiO and B-OMe-UiO. This difference in SP-to-MC photo-isomerization kinetics was most likely due to the presence of the methoxy group on the nitrobenzopyran moiety. The electron-donating nature of the methoxy group in 2 and 4 could destabilize MC form and facilitated faster thermal back-isomerization to the SP state than 1 and 3. In contrast, the electron-donating carboxylic acid group on the indoline pyrrole could promote SP-to-MC conversion. Hence, the alkylated carboxylic acid (–$(CH_2)_2$) in 1 and 2 resulted in a more stabilized MC state than direct attachment of the carboxylic acid group on the indoline benzene in 3 and 4. From UV–vis diffuse reflectance analyses, we also observed that UV irradiation increased absorbance within the visible (450 – 550 nm) region, corresponding to SP-to-MC photo-isomerization. The absorbance returned to the original state or to a slightly lower level than the non-photo-irradiated MOF sample. This behavior suggested that a small fraction of the MC isomer was already present in the pristine state. To further explain the observed colorimetric changes of SP-UiOs, we simulated their UV–vis absorption spectra based on optimized cluster models using TD-DFT calculations (Figure 2e – h). These computational insights supported the experimentally observed photochromic behaviors of SP-UiOs studied in this work.

2.2 Photo-responsive properties of SP-UiO MOFs

2.2.1 Photographs of color changes of 1 – 4 in DMF solution

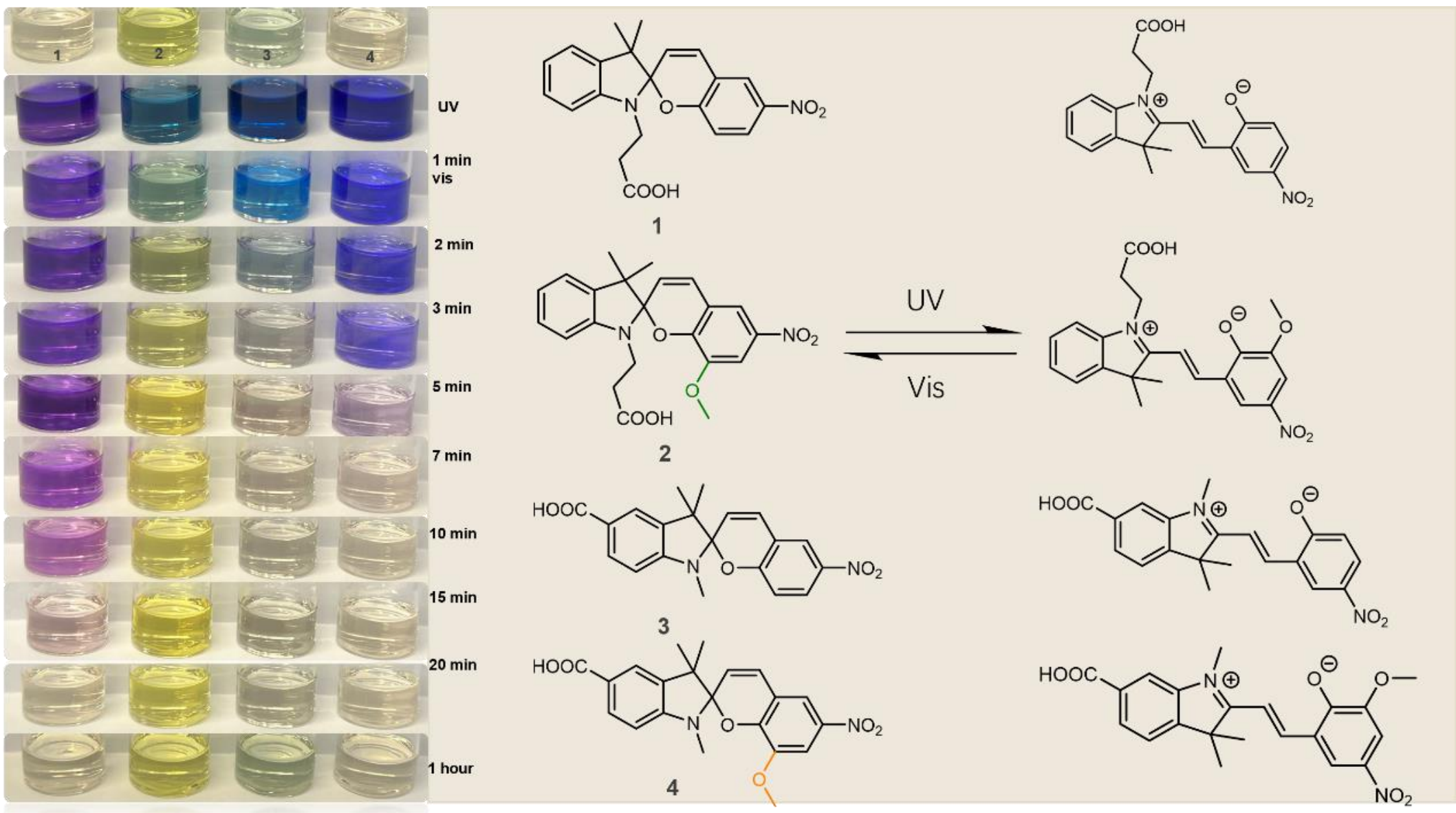

Figure S17. The photoswitching behaviors of four different SP linkers in DMF solution. The photoswitching behaviors of four different SP linkers were first characterized in DMF solution. Upon exposure to UV light, all SP linkers rapidly isomerized to the open-ring MC form in deeper color, and gradually reverted to their original SP state upon standing in the dark.

However, the recovery times varied significantly depending on their molecular structures. This difference is likely due to the presence of an electron-donating methoxy group on the chromene moiety of **2**, which destabilizes the MC form and thus facilitates faster thermal back-isomerization to the SP state. In contrast, an electron-donating group on the indoline portion of the spiropyran structure tends to promote the SP-to-MC conversion. Therefore, compared with a directly connected electron-withdrawing carboxyl group in benzene ring (**3** and **4**), the longer alkyl spacer in SPCO (–$(CH_2)_2COOH$) results in a more stabilized MC state.

2.2.2 Simulated photo-modulated pore size distributions of SP-UiO MOFs

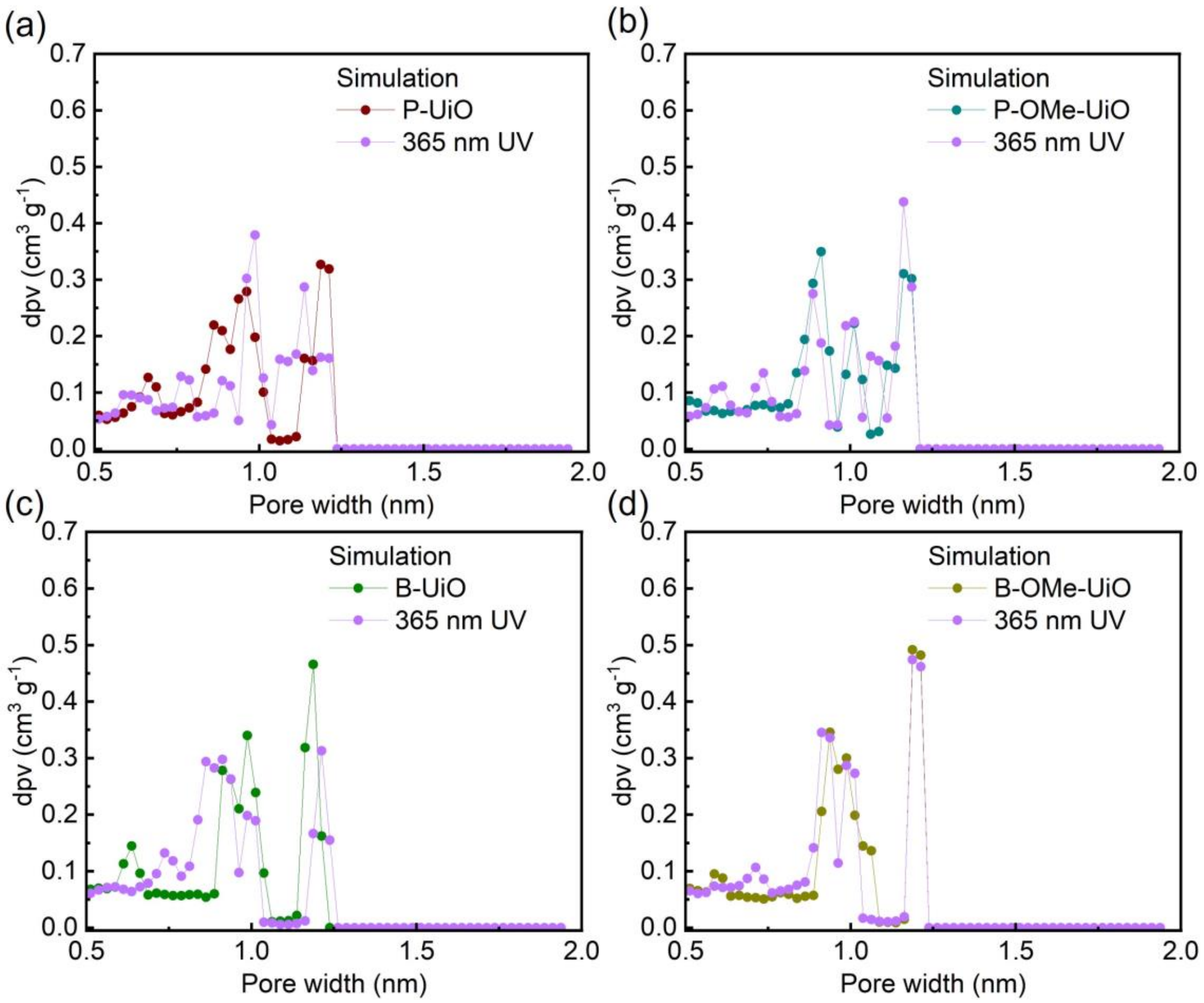

Figure S18. Simulated pore size distributions of (a) P-UiO, (b) P-OMe-UiO, (c) B-OMe-UiO and (d) B-UiO MOFs at both SP and MC states.

As shown in the N_2 adsorption–desorption isotherms (Figure 2), UV exposure leads to a noticeable decrease in N_2 uptake in the low-pressure region, indicating a reduced micropore volume. Correspondingly, both the calculated BET surface area and total pore volume showed a notable decrease after UV irradiation. For instance, the BET surface areas of SPCO-UiO and SPOMeCO-UiO drop from 1622 and 1632 m^2 g^{-1} to 1310 and 1432 m^2 g^{-1}, respectively. This decrease is attributed to the photoisomerization of SP to the more extended MC form, which likely occupies more pore space or restricts gas diffusion within the framework. However, the extent of this reduction varied depending on the type of SP grafted. Samples with SP moieties coordinated at defect sites exhibited more pronounced decreases in surface area and pore volume. For instance, SPCO-UiO and SPOMeCO-UiO exhibit up to 19% and 12% decrease in surface area and 18% and 11% in pore volume. This can be attributed to the higher photoisomerization efficiency when SP was grafted by defect, which benefit from reduced steric hindrance and greater conformational flexibility. Moreover, all UV induced porosity changes are fully reversible upon mild thermal degassing, confirming the structural resilience and photo responsive reversibility of these materials. The pore size distribution of SP-UiO samples before and after UV light irradiation were also measured at 77 K (Figure 3e-h). Results suggested that the mainly pore sizes still centered at approximately 0.5 and 1.2 nm after SP to

MC transition. However, a slight decrease in relative pore volume was observed. This indicates that the transition to the more extended MC form partially obstructs the existing pore channels, and this increased steric hindrance and polarity reduce the accessible pore volume.
The simulated pore size distributions, calculated from DFT-optimized crystal structures of the lowest-energy SP and MC configurations also show a decrease in relative pore volume upon isomerization.

2.3 Photo-reversible electronic modulation of P-OMe-UiO Lewis acidity

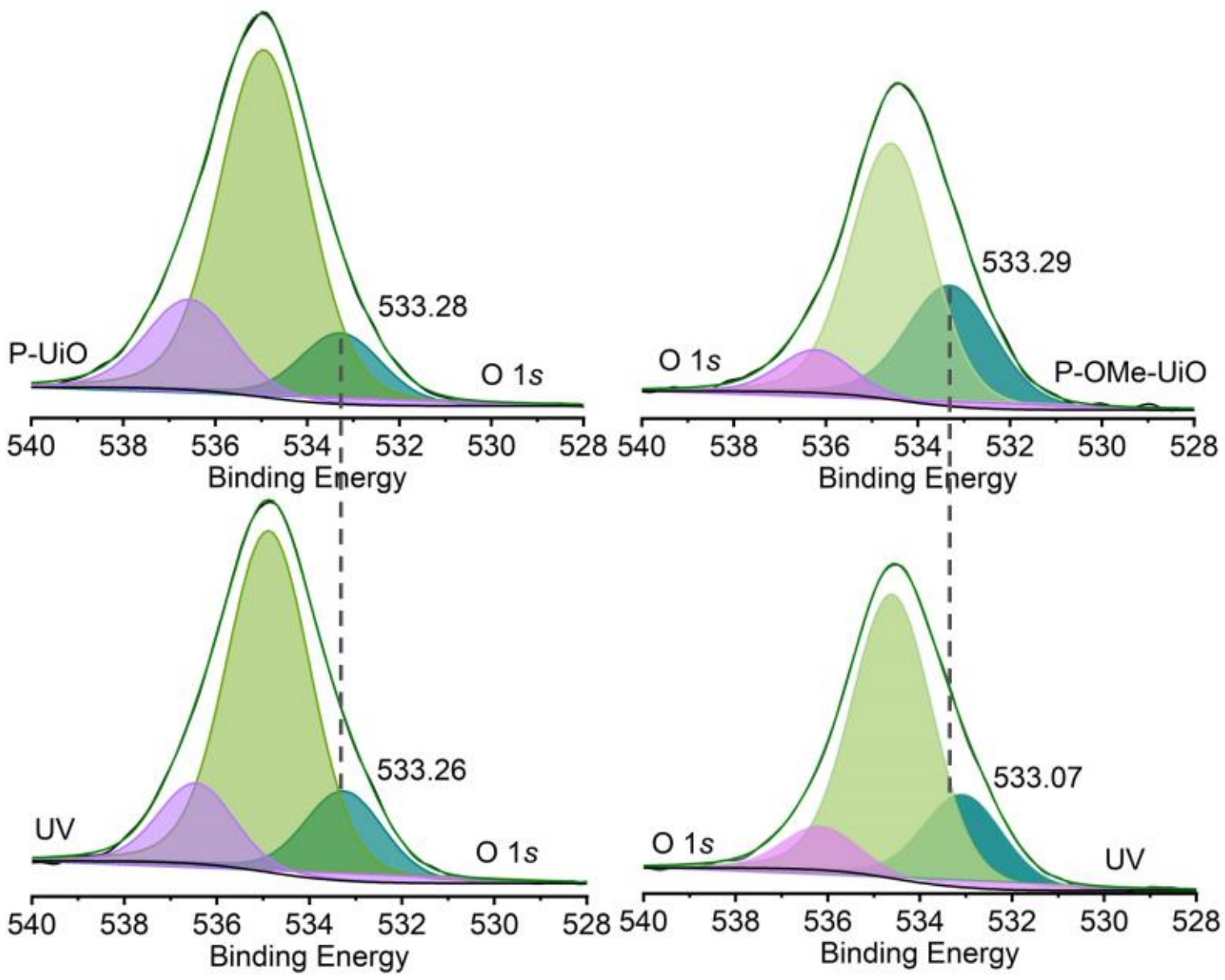

Figure S19. *Ex situ* O 1*s* of P-UiO and P-OMe-UiO before and after UV light irradiation.
UV irradiation caused the O 1*s* binding energies of P-UiO and P-OME-UiO to shift to lower values, consistent with the photoinduced ring-opening of SP to generate phenoxide ions. The formation of these anionic species enhances the electron density around oxygen, leading to a reduction in binding energy.

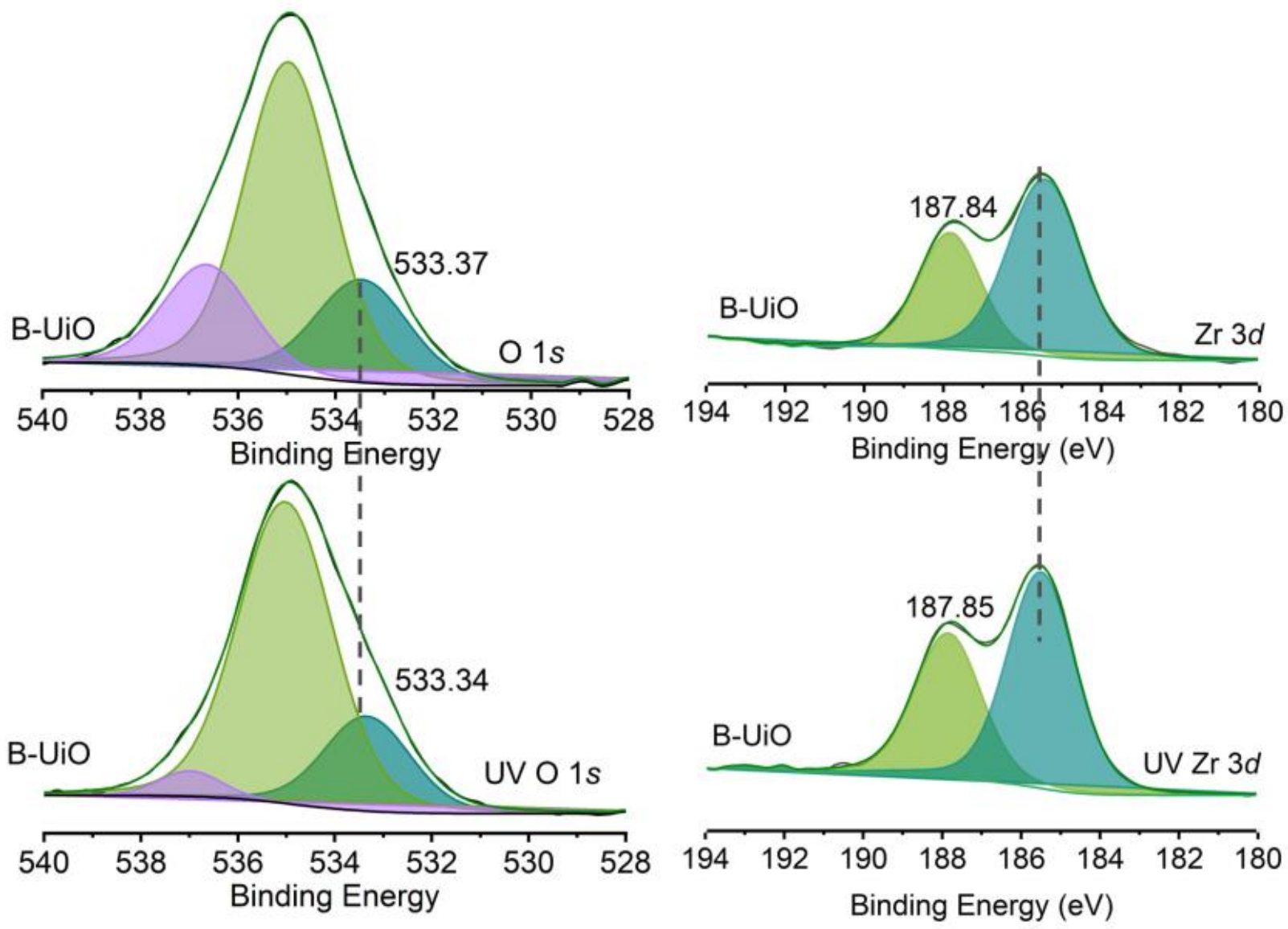

Figure S20. *Ex situ* O 1*s* and Zr 3$d_{5/2}$ XPS spectra of B-UiO before and after UV light irradiation. After UV irradiation, the O 1*s* binding energies of B-UiO also shift to lower values, consistent with the photoinduced ring-opening of SP to generate phenoxide ions. However, In contrast to P-OMe-UiO, the Zr 3$d_{5/2}$ binding energy of B-UiO remains constant upon UV irradiation, suggesting that the ring-opening process does not result in the generation of Zr–MC species in this sample.

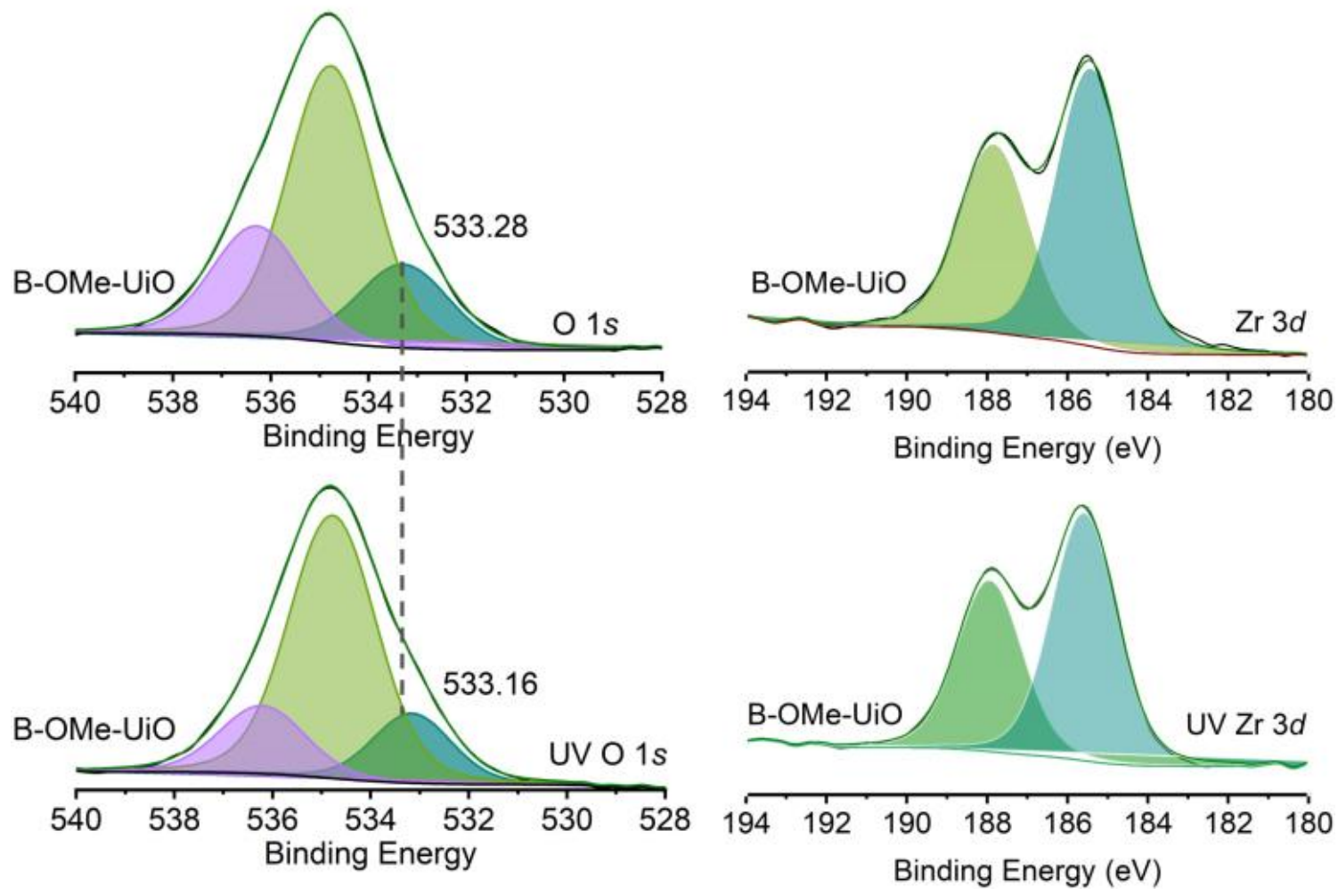

Figure S21. *Ex situ* O 1*s* and Zr 3$d_{5/2}$ XPS spectra of B-OMe-UiO before and after UV light irradiation. After UV irradiation, the O 1*s* binding energies of B-OMe-UiO also shift to lower values, consistent with the photoinduced ring-opening of SP to generate phenoxide ions. However, In contrast to P-OMe-UiO, the Zr 3$d_{5/2}$ binding energy of B-OMe-UiO move to higher position, suggesting that the ring-opening process does not result in the generation of Zr–MC species in this sample.

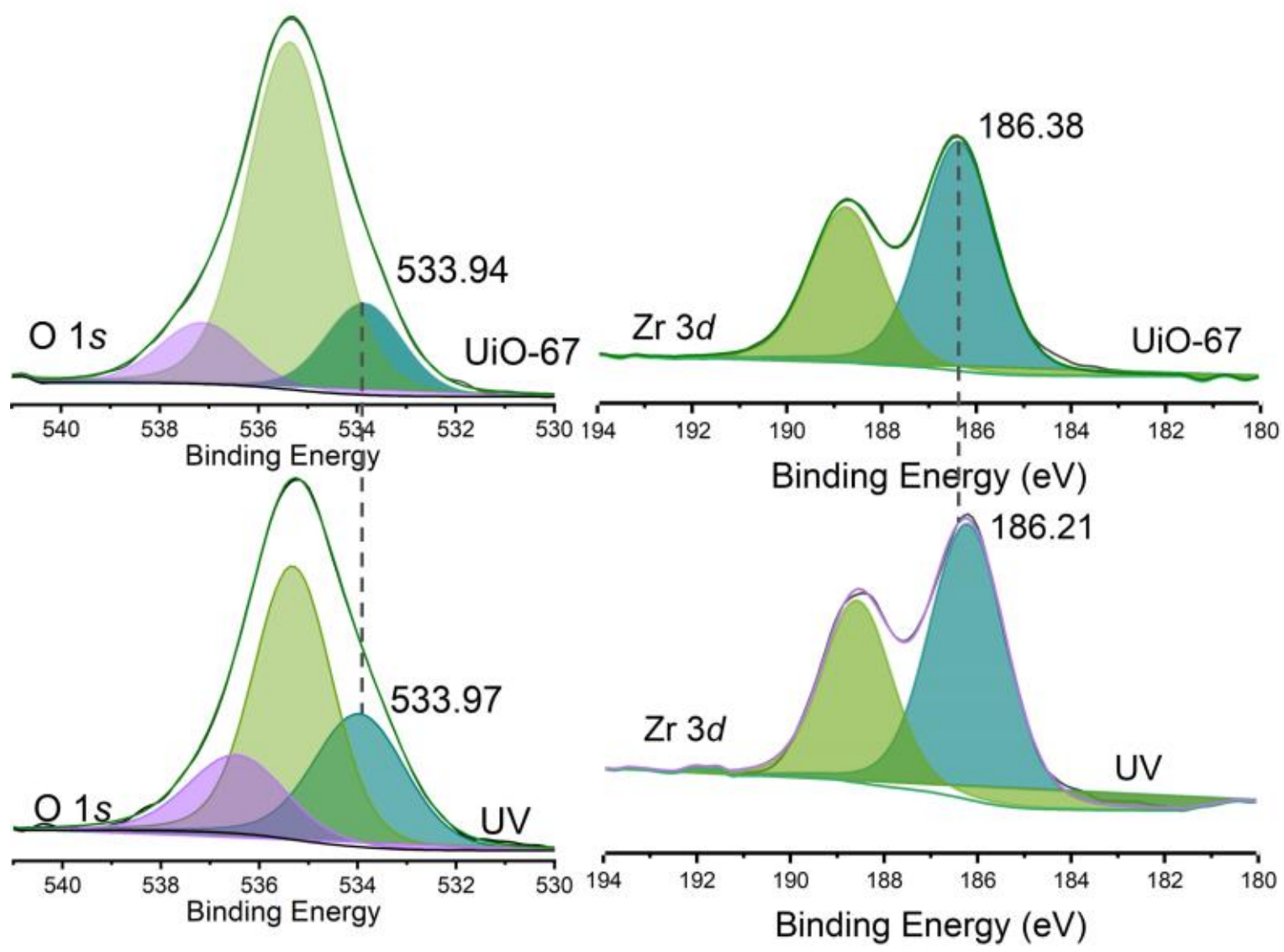

Figure S22. *Ex situ* O 1*s* and Zr 3$d_{5/2}$ XPS spectra of UiO-67 before and after UV light

irradiation. The O 1*s* binding energies of UiO-67 show negligible change upon UV irradiation. This further validates that the UV-induced shifts in O 1*s* binding energies in SP-functionalized UiO samples originate from the ring-opening of SP to generate MC species.The Zr 3$d_{5/2}$ peak of parent UiO-67 shifted to lower binding energy after UV irradiation, which was attributed to the partical degradation of this defective UiO-67 and the aggregation of Zn-Zn after long UV light irradiation for *Ex situ* analysis.

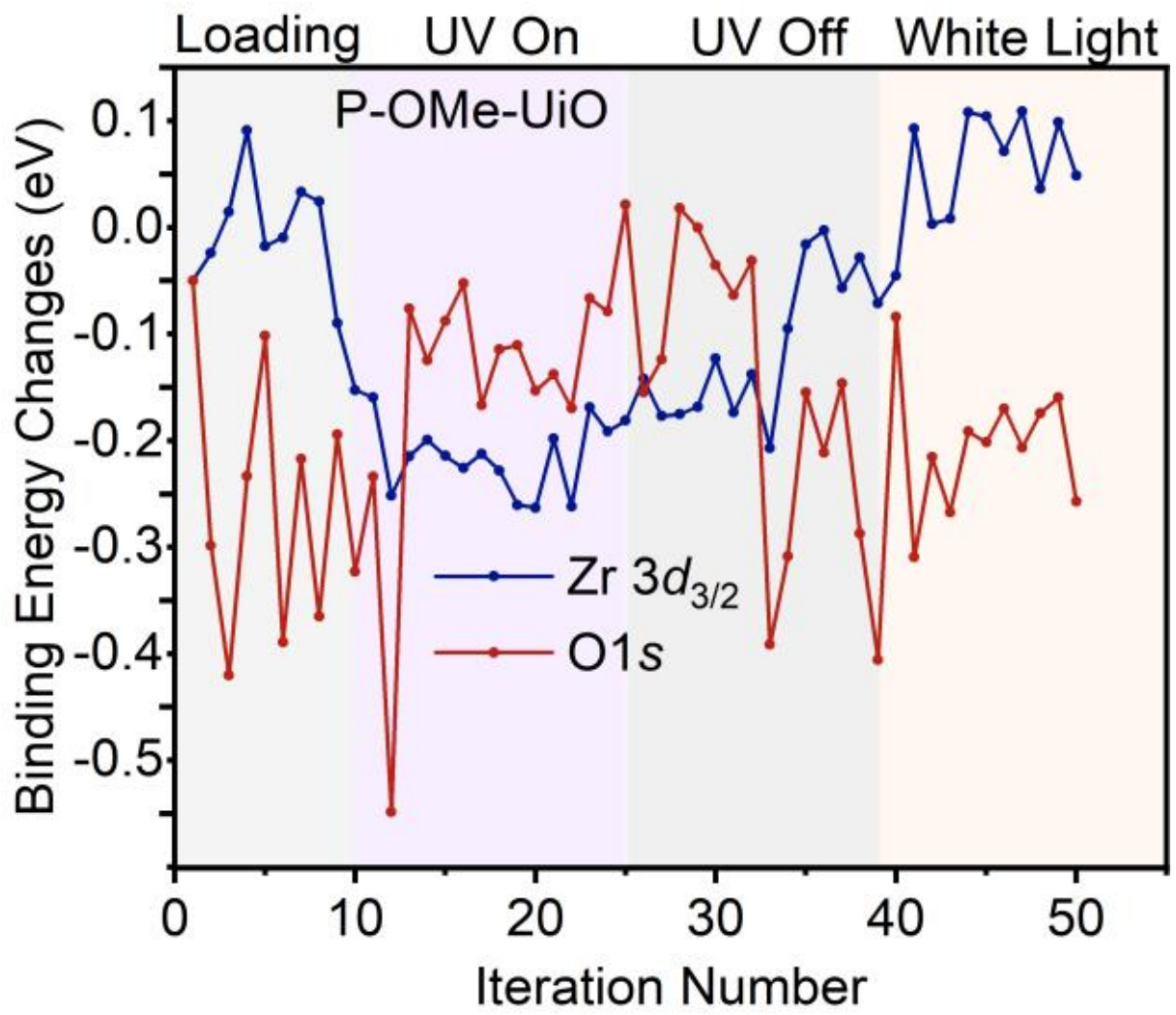

Figure S23. In-situ O 1*s* and Zr 3$d_{5/2}$ XPS spectra of parent P-OMe-UiO with UV switched on/off and lateral white light irradiation.
Iteration 1 – 9 = Loading phase
Iteration 10 – 24 = UV light on
Iteration 25 – 39 = UV light off, sample in darkness
Iteration 40 – 49 = White light on

Here we tracked the changes in O 1*s* and Zr 3$d_{5/2}$ binding energies of P-OMe-UiO over different phases. During the loading phase (iteration 1 – 9), P-OMe-UiO comprised predominantly of SP, the thermodynamically stable form. Upon UV irradiation at iteration 10, the O 1*s* binding energy decreased by ~ 0.5 eV. This indicated formation of electron-rich phenolate oxygen during SP-to-MC ring-opening isomerization. Over the subsequent iterations (11 – 23), the O 1*s* binding energy progressively increased by +0.12 eV while the Zr 3$d_{5/2}$ binding energy decreased by -0.20 eV. This anti-correlated behavior provides direct evidence of MC-Zr coordination: as the phenolate oxygen donates electron density to Zr (reducing Zr electron affinity and lowering its binding energy), the oxygen becomes less electron-rich (increasing O 1*s* binding energy). When UV light was switched off (iterations 25 – 39), both binding energies slowly reverted toward their original values. This was indicative of thermal MC-to-SP back-isomerization and dissociation of the MC-Zr complex. This recovery was further enhanced under white light irradiation (iterations 40 – 49), with binding energies returning to 90 – 95% of their initial loading values. This demonstrated excellent photo-reversibility.

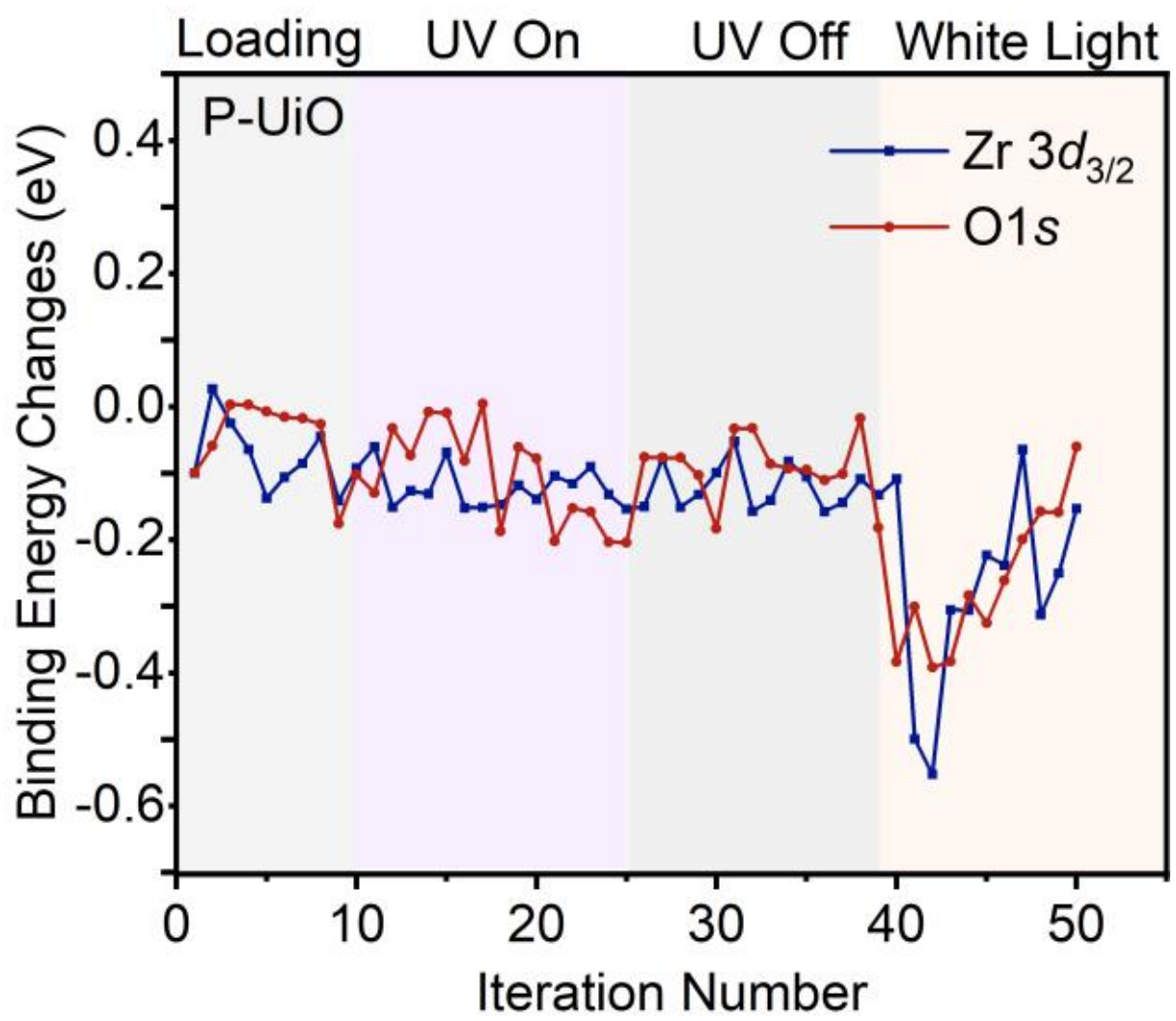

Figure S24. In-situ O 1*s* and Zr 3$d_{5/2}$ XPS spectra of parent P-UiO with UV switched on/off and lateral white light irradiation.
Iteration 1 – 9 = Loading phase
Iteration 10 – 24 = UV light on
Iteration 25 – 39 = UV light off, sample in darkness
Iteration 40 – 49 = White light on

The O 1*s* and Zr 3$d_{5/2}$ binding energies of P-OMe-UiO over different phases were also investigated. Upon UV light irradiation, there is an average 0.6 eV decrease of O 1*s* binding energy compared to the loading phase. This indicated formation of electron-rich phenolate oxygen during SP-to-MC ring-opening isomerization. Over the subsequent darkness iterations (11 – 23), both the O 1*s* binding energy and the Zr 3$d_{5/2}$ binding energy keeps stable, the average changes of binding energy is smaller than 0.01eV. These results indicate that no direct experimental evidence of anti-correlated behavior arising from MC–Zr coordination is observed in the P-UiO system, thereby highlighting the unique light swing MC-Zr coordination behavior of P-OMe-UiO. After white light exposure, both the average binding energy of O 1*s* and Zr 3$d_{5/2}$ decreased by 0.1eV compared with the dark condition. This behavior may originate from the generation of photoinduced electrons, which increases the local electron density and consequently leads to a shift of the binding energy toward lower values.

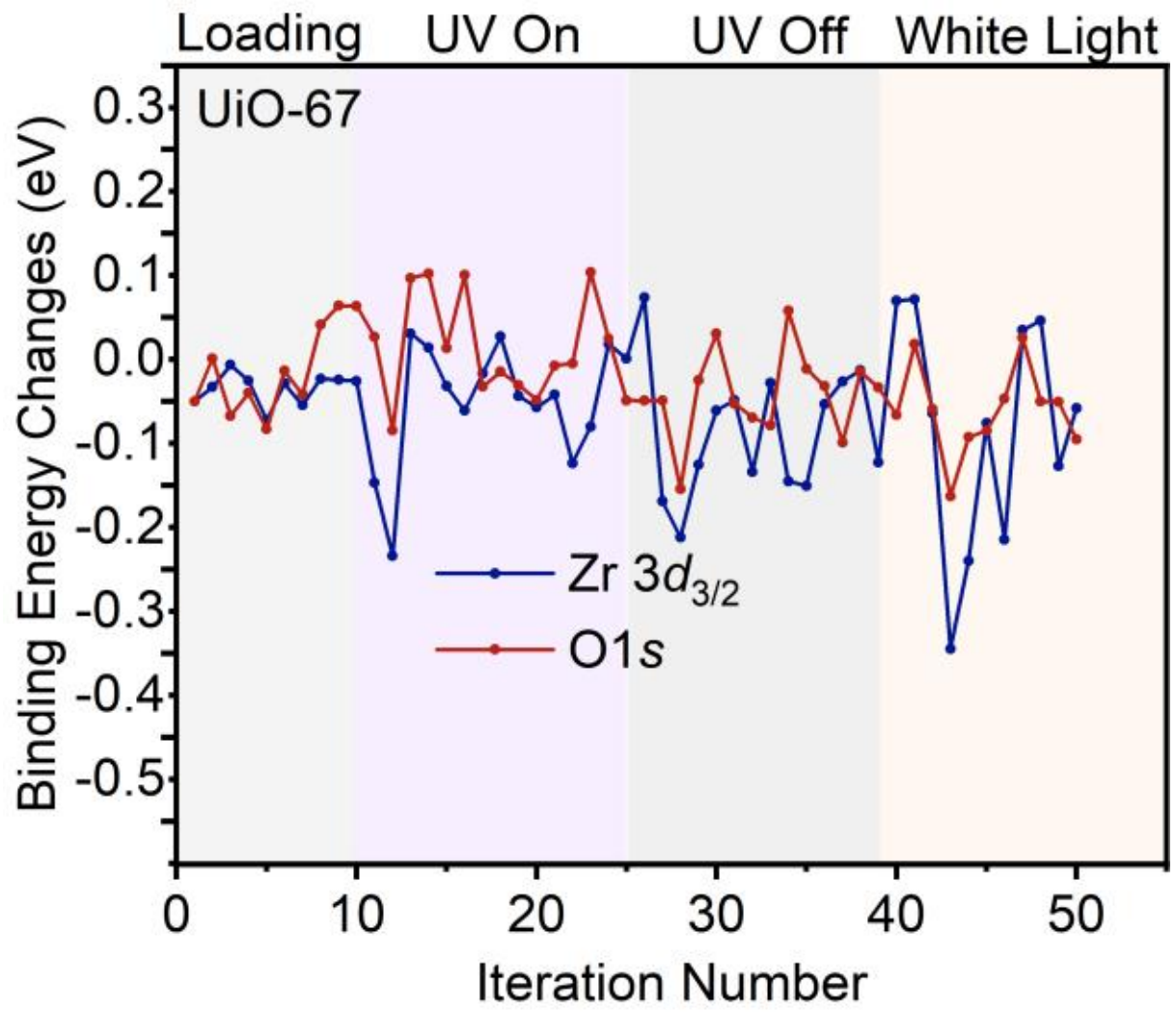

Figure S25. In-situ O 1s and Zr 3$d_{5/2}$ XPS spectra of parent UiO-67 with UV switched on/off and lateral white light irradiation.
Iteration 1 – 9 = Loading phase
Iteration 10 – 24 = UV light on
Iteration 25 – 39 = UV light off, sample in darkness
Iteration 40 – 49 = White light on

Both the O 1s binding energy and the Zr 3$d_{5/2}$ binding energy remains stable upon UV light irradiation and subsequent darkness iterations, the average changes of binding energy is smaller than 0.02eV. As a control experiment, this observation further confirms the reversible light swing MC-Zr coordination behavior in P-OMe-UiO. Similar to P-UiO, white-light irradiation also induces the generation of photoinduced electrons, resulting in a shift of the binding energy toward lower values of both O 1s and Zr 3$d_{5/2}$.

2.4 CO_2 adsorption isotherms of SP-UiO MOFs

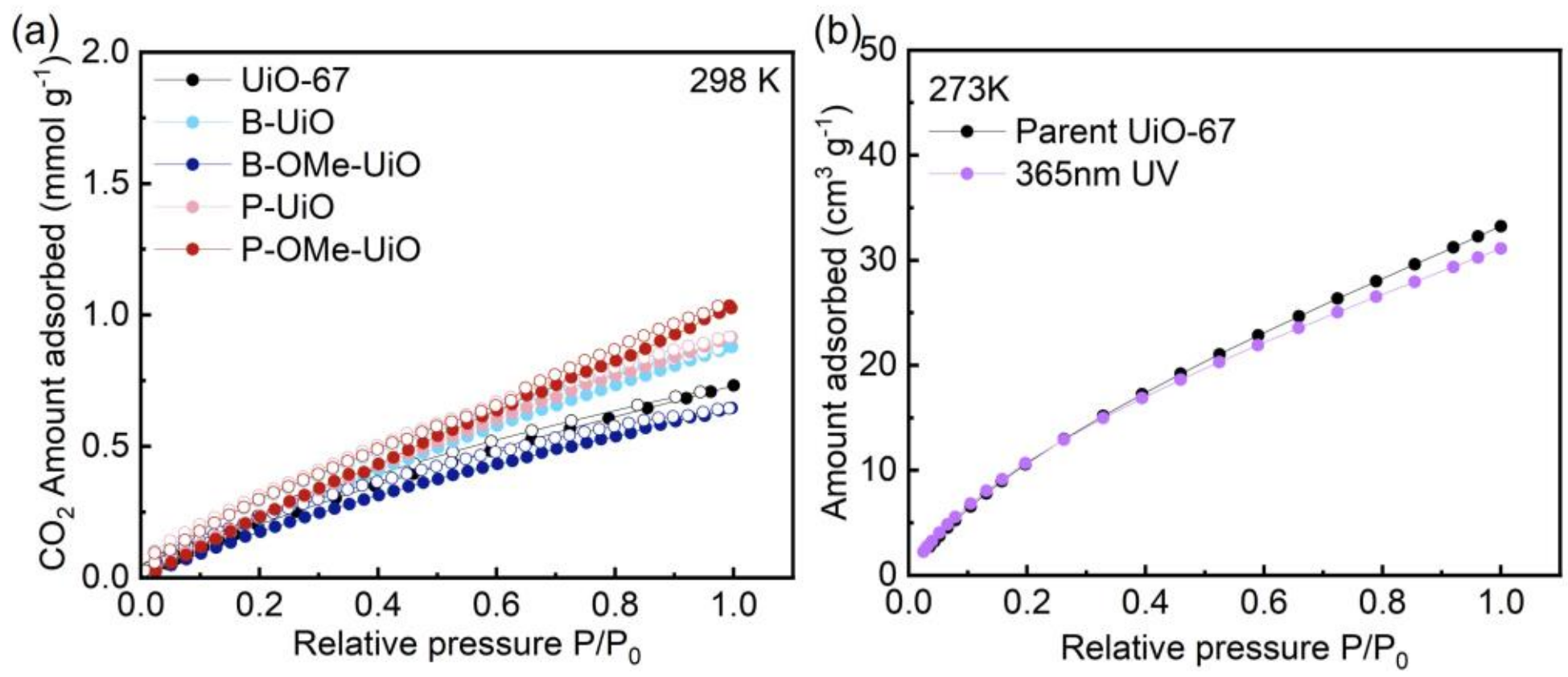

Figure S26. (a) CO_2 adsorption–desorption isotherms of parent defective UiO-67 MOF (black), P-UiO (red), P-OMe-UiO (blue), B-UiO (green) and B-OMe-UiO (purple) at 298 K. (b) CO_2 adsorption isotherms of parent UiO-67 in the darkness (black) and right after UV light irradiation and in-situ UV conditions at 273 K (purple)

The results show that the incorporation of SP molecules significantly enhances CO_2 uptake in SPOMeCO-UiO and SPCO-UiO, reaching capacities of up to 2.4 and 2.15 mmol g^{-1}, respectively at 273 K. This improvement is attributed to multiple synergistic effects: the coordination of SP molecules not only repairs structural defects but also exposes additional open metal sites[14] and introduces new Lewis base interaction sites, such as tertiary amines, methoxy oxygens, and nitro groups and then contribute to higher CO_2 binding affinity[15]. Additionally, the maintained high surface area and porosity of SPOMeCO-UiO and SPCO-UiO further facilitate CO_2 adsorption performance. Notably, even at 298 K, SPOMeCO-UiO still maintains a CO_2 capacity of 1.0 mmol g^{-1}. In contrast, COSP-UiO and COSPOMe-UiO exhibit only slightly increases in CO_2 uptake (2.0 and 1.8 mmol g^{-1}, respectively), primarily due to the significant reduction in surface area and pore volume. The parent UiO-67 without any SP incorporation was also estimated with the same condition of SP-UiOs. As expected, the parent framework showed negligible change in CO_2 uptake, confirming that the observed switching in SP-UiO samples arises exclusively from the incorporated SP moieties rather than instrumental variation or degradation in the framework itself. The slightly decrease upon 0.5-1 relative pressure regin likely due to aggregation after long time UV light exposure because the defective structure.

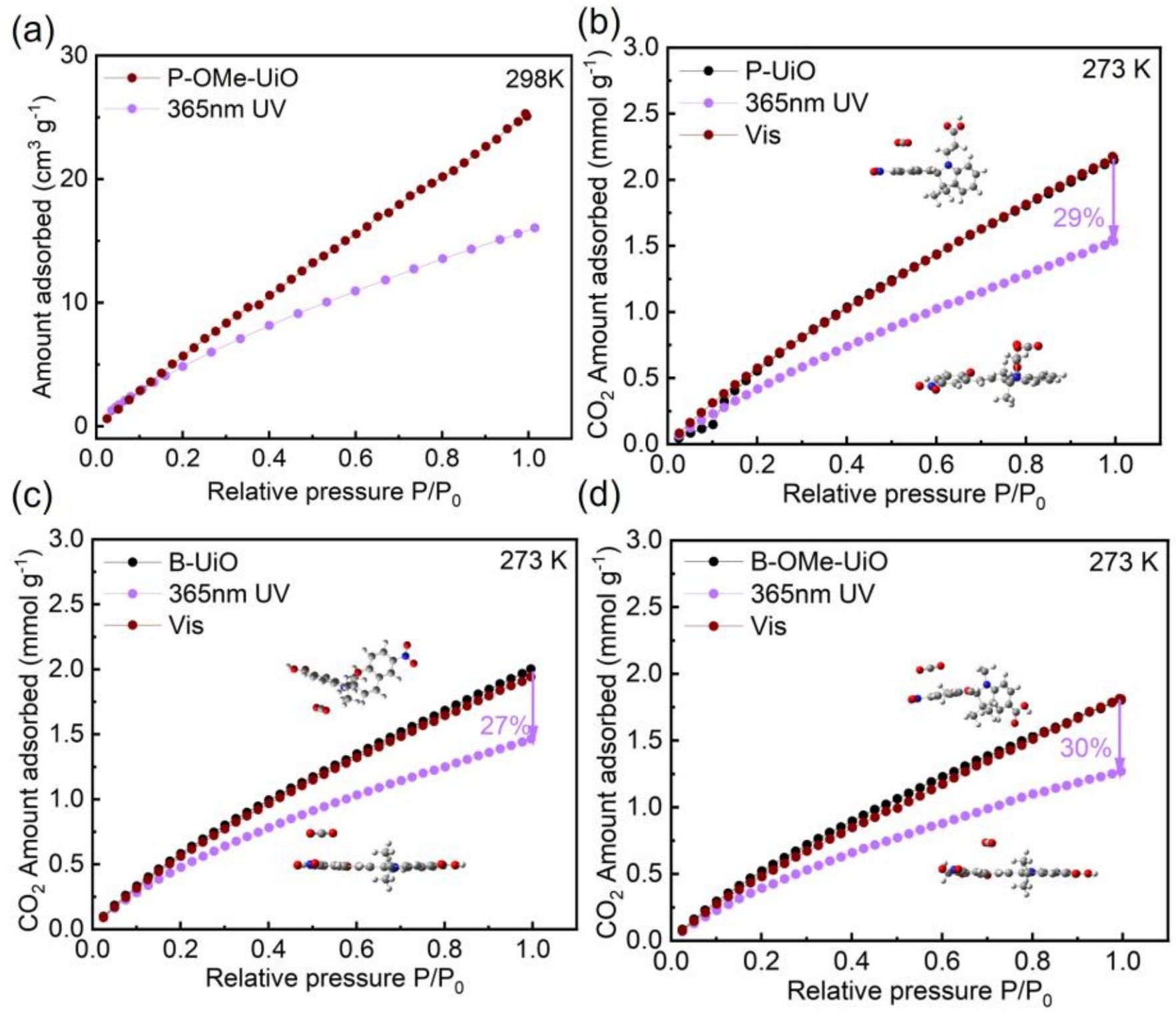

Figure S27. CO_2 adsorption isotherms comparing materials before (365 nm UV) and after (450 nm visible light) photo-irradiation of (a) P-OMe-UiO at 298 K, (b) P-UiO, (c) B-UiO and (d) B-OMe-UiO at 273 K. All materials show Type I behavior with reduced uptake in MC state.

As shown in results, all SP-functionalized UiO-67 samples exhibited a remarkable decrease in CO_2 uptake under UV exposure, indicating effective photoinduced switching behavior. However, the CO_2 switching efficiency varies depending on the different type of SP grafted and different functional group in SP. Notably, P-UiO and P-OMe-UiO exhibited more pronounced decreases in CO_2 uptake upon UV light irradiation compared to B-UiO and B-OMe-UiO.

Specifically, the CO_2 capacity of P-UiO reversibly swings from 2.15 to 1.76 mmol g^{-1} and P-OMe-UiO from 2.70 to 1.62 mmol g^{-1} at 273 K, corresponding to light-regenerated working capacity up to 0.6 and 1.1 mmol g^{-1}, respectively. These results suggested a more energy efficient way to regenerate the CO_2 adsorbents by a sustainable light resource compared to conventional regeneration methods. As previously mentioned, this more pronounced CO_2 switching efficiencies observed in P-UiO and P-OMe-UiO could be attributed to the higher photoisomerization efficiency and pore volume changes upon UV exposure, which benefit from reduced steric hindrance and greater conformational flexibility. Such light-induced pore modulation contributing to light-swinged CO_2 adsorption behavior has also been reported in the previous work. Although the isomerization process of B-UiO and B-OMe-UiO were partially restricted due to aggregation within confined pores, their CO_2 switching efficiencies still can reach up to 27% and 30%, suggesting a CO_2 working capacity of 0.5 mmol g^{-1}. Additionally, comparing with P-UiO, the presence of methoxy groups in P-OMe-UiO further contributed to the highest CO_2 switching efficiency up to 40%. Therefore, the functionalization of SP in porous materials is also promising, common, and effective way to enhance the switching efficiency of current materials.

2.5 Properties of SP-UiO MOFs after cyclic testing

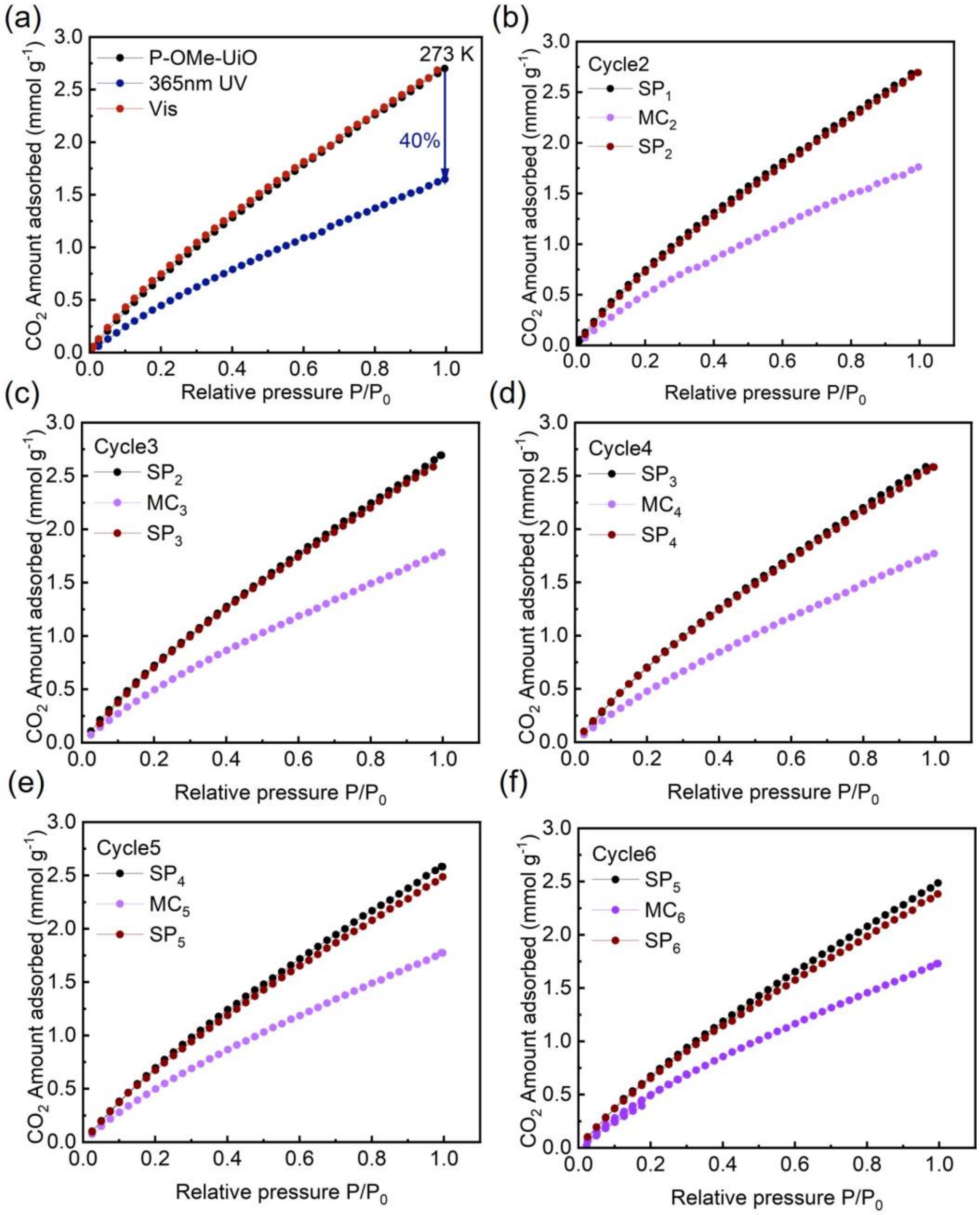

Figure S28. The CO_2 adsorption capacity of P-OMe-UiO after the cyclic UV(●)-visible (●) light irradiation. The UV-vis irradiation process is used here to enable a CO_2 uptake-release process. After the 6th cycle of UV-vis irradiation, the CO_2 adsorption capacities retaines 88% of initial SP-state CO_2 uptake.

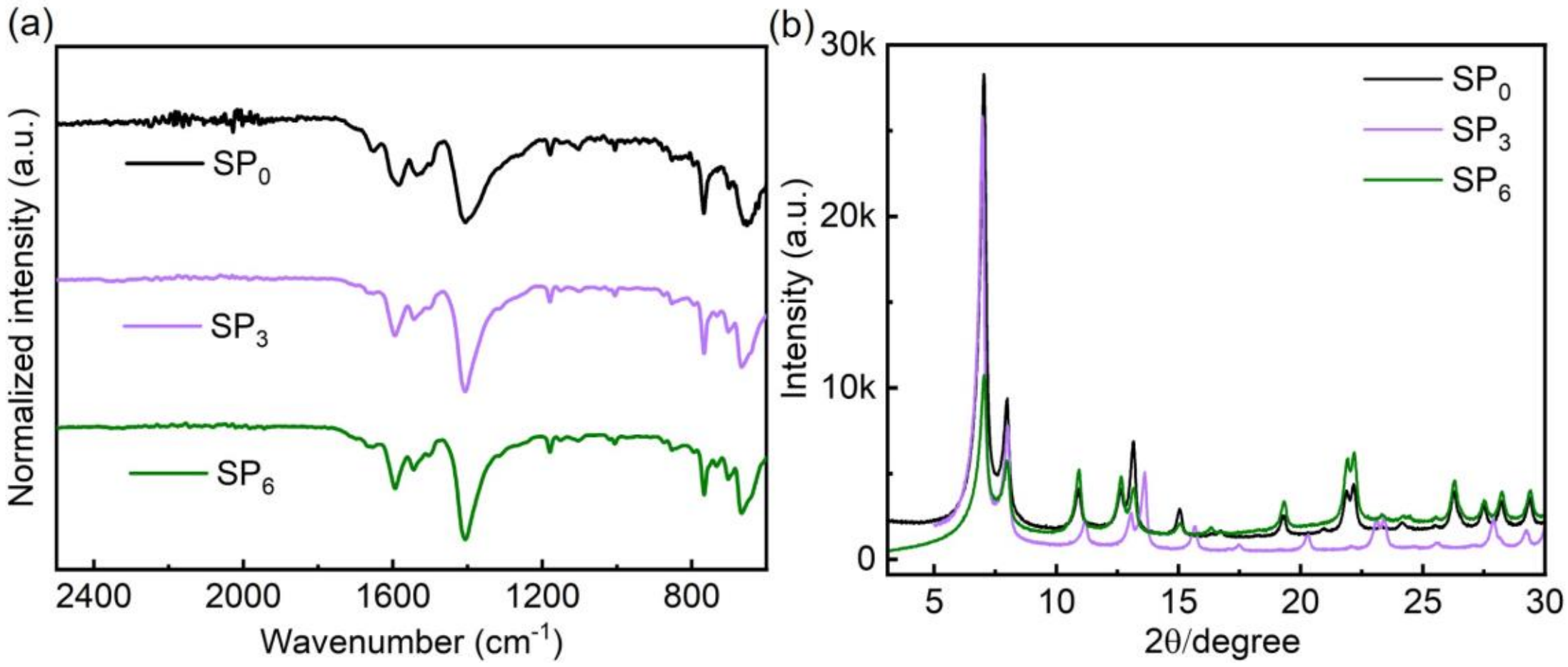

Figure S29. (a) FTIR of P-OMe-UiO after 3 and 6 cyclic testing. (b) PXRD patterns of P-OMe-UiO collected at different photo switching cycle stages. FT-IR spectra showed no observable changes after cycles, indicating no chemical degradation of the framework or the SP units. PXRD pattern of P-OMe-UiO after cyclic testing. The PXRD patterns also retained their primary features after cycling, with only a modest broadening of approximately 4% in full width at half maximum (FWHM). All characteristic diffraction peaks are fully retained with no shift in peak positions, confirming that the UiO-67 framework topology and unit cell dimensions are intact after three cycles. The slight increase in FWHM corresponds to a minor reduction in coherent domain size, attributable to localised MC aggregation within the pore channels. After extending cycling experiments to 6, a more pronounced reduction in primary peak intensity is observed, broadly corresponding to the 35% reduction in working capacity recorded at SP6. We attribute this to the cumulative photochemical effects of prolonged UV irradiation: each operando CO_2 adsorption measurement requires approximately 2.5–3 hours of continuous UV irradiation to maintain the MC state, as MC undergoes spontaneous thermal relaxation to SP in the absence of light. This extended irradiation progressively bleaches the SP chromophore and promotes MC aggregation, reducing the population of photoswitchable SP units and increasing diffuse X-ray scattering at low angles. Critically, however, all characteristic framework diffraction peaks remain present and unshifted at SP6, and FTIR spectra recorded after six cycles show no changes in the framework absorption bands.

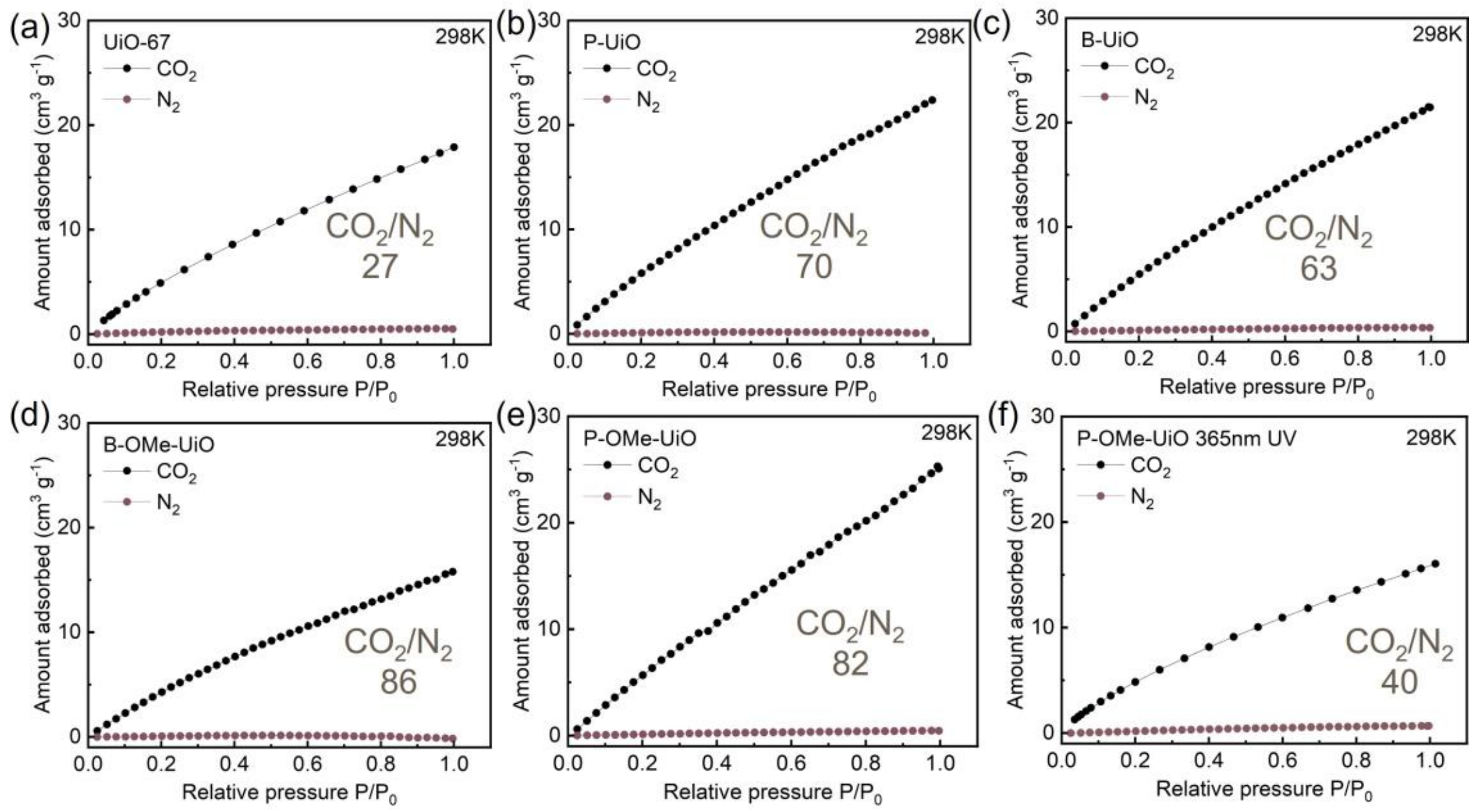

Figure S30. The N_2 and CO_2 adsorption isotherms of (a) parent UiO-67; (b) P-UiO; (c) B-UiO; (d) B-OMe-UiO; (e) P-OMe-UiO and (f) P-OMe-UiO after UV at 298 K.The CO_2/N_2 selectivity of all SP-UiO samples was investigated by testing the N_2 and CO_2 adsorption isotherms. As shown in Figure S30, all SP-UiO samples exhibited markedly lower N_2 uptake, indicative of a preferential binding affinity for CO_2 molecules and thus a high intrinsic CO_2/N_2 selectivity. The CO_2/N_2 selectivity values were investigated by Henry's constants under low-pressure conditions. The parent UiO-67 showed the lowest CO_2/N_2 selectivity. Incorporation of various SP moieties not only enhanced the CO_2 uptake capacity but also significantly improved CO_2/N_2 selectivity. Notably, the CO_2/N_2 selectivity of P-OMe-UiO increased by 200% compared to parent UiO-67.

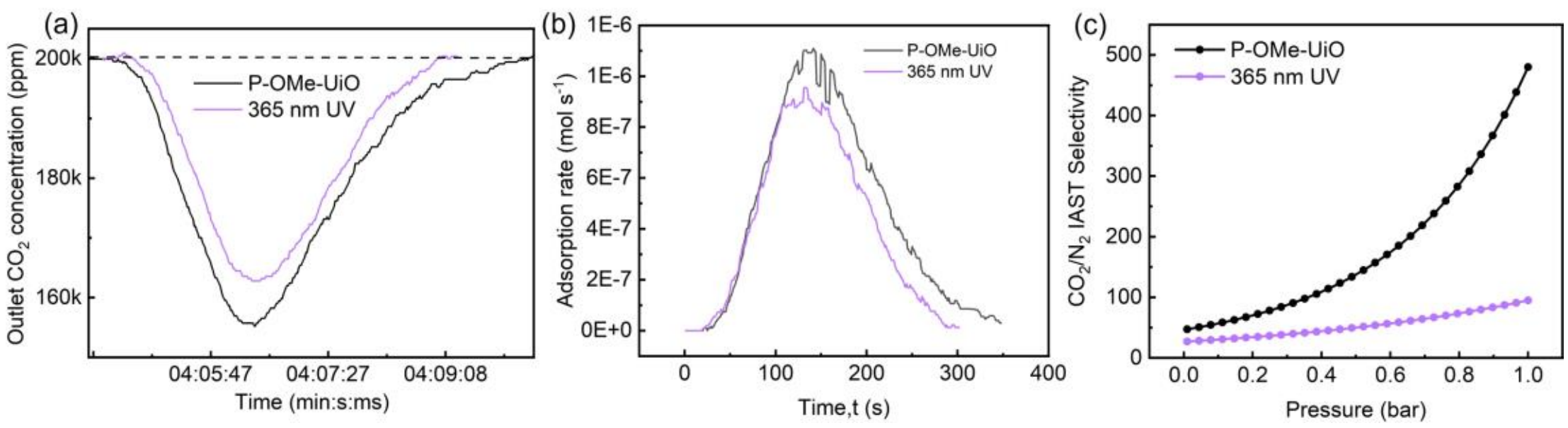

Figure S31. (a) Breakthrough experiments of P-OMe-UiO using a simulated flue gas mixture containing 20 vol% CO_2 in N_2. The gas stream was passed through a quartz tube packed with 400 mg of P-OMe-UiO, and the outlet CO_2 oncentration was monitored over time. (b) CO_2 adsorption rate of P-OMe-UiO using a simulated flue gas mixture containing 20 vol% CO_2 in N_2. (c) IAST CO_2/N_2 selectivity of P-OMe-UiO after UV calculated using 20:80 CO_2 and N_2 mixtures at 298 K. After UV irradiation, the CO_2 adsorption rate of the P-OMe-UiO decreased, further corroborating the weaker CO_2 adsorption capability in the MC state. When the gas mixture passed through the cell, a pronounced decrease in the outlet CO_2 concentration was observed, indicating that the sample possesses the ability to adsorb CO_2 from the mixed gas stream. A larger reduction in the outlet CO_2 concentration corresponds to a stronger adsorption

capacity. Upon UV irradiation, this decrease became less pronounced, suggesting a weakened CO_2 adsorption capability. This behavior further confirms the reduced CO_2 adsorption performance in the MC state.

2.6 Detailed discussion of LCA outcomes

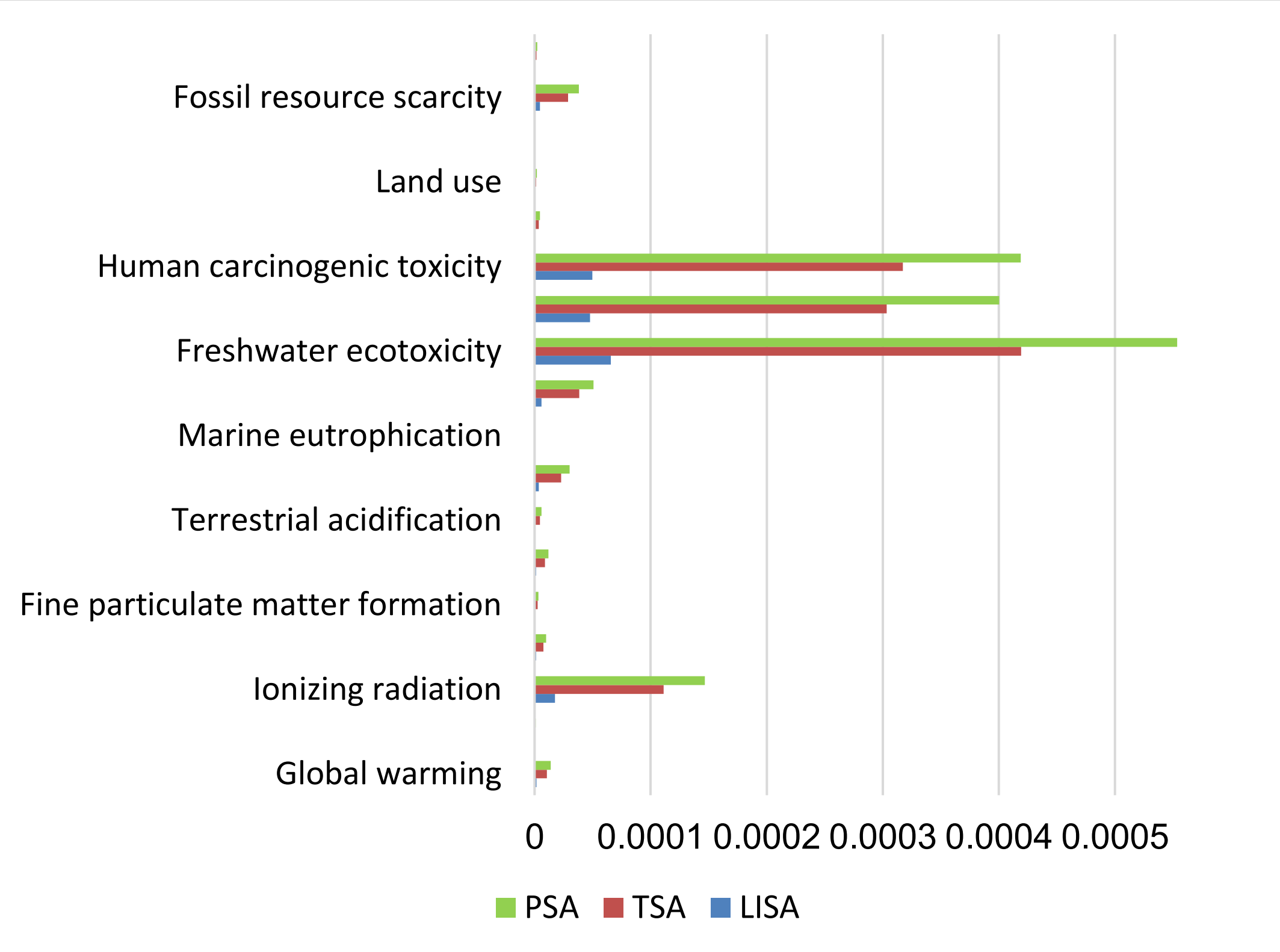

Figure S32. Comparison of midpoint environmental impacts from the operation and regeneration energy consumption of LISA, TSA, and PSA.

Figure S32 compares the normalized midpoint environmental impacts associated with the energy consumption of LISA, TSA, and PSA. Across nearly all impact categories, PSA exhibits the highest environmental burdens, followed by TSA, while LISA consistently demonstrates the lowest impacts. The results are tabulated in **Table S5**. This ranking is strongly correlated with the regeneration energy requirements of each process. Although PSA requires substantially fewer cycles due to its high CO_2 working capacity, its regeneration step is highly energy-intensive because of the significant compressor and vacuum pump work needed to operate at pressures up to 30 bar. As a result, PSA shows the greatest impacts in categories such as human carcinogenic toxicity, freshwater and marine ecotoxicity, and ionizing radiation. These elevated values are further explained by the structure of the United Kingdom electricity mix, which remains heavily dependent on fossil fuels and nuclear energy, with renewable sources accounting for only a minor share [16].

TSA shows moderate impacts that are significantly higher than LISA but lower than PSA. Although the per-cycle energy consumption for TSA is much lower than PSA, the number of required cycles per functional unit is higher due to its lower working capacity. Additionally, because TSA regeneration relies heavily on thermal energy, the environmental profile is sensitive to the electricity mix used to supply heat.

LISA demonstrates the lowest impacts predominantly due to its very low per-cycle energy demand which is only 60 J, despite having the smallest working capacity among the three processes. The cumulative energy required to capture 1 mol CO_2 is therefore significantly lower, producing correspondingly smaller environmental burdens. In the human carcinogenic toxicity category, for example, the environmental impact of LISA is approximately eight times lower than PSA and six times lower than TSA, reflecting the direct proportionality between energy consumption and environmental emissions. Similar downward trends are observed across ecotoxicity, ionizing radiation, and fossil resource scarcity categories, reaffirming that regeneration energy is the dominant contributor to environmental impacts in adsorption-based CO_2 capture.

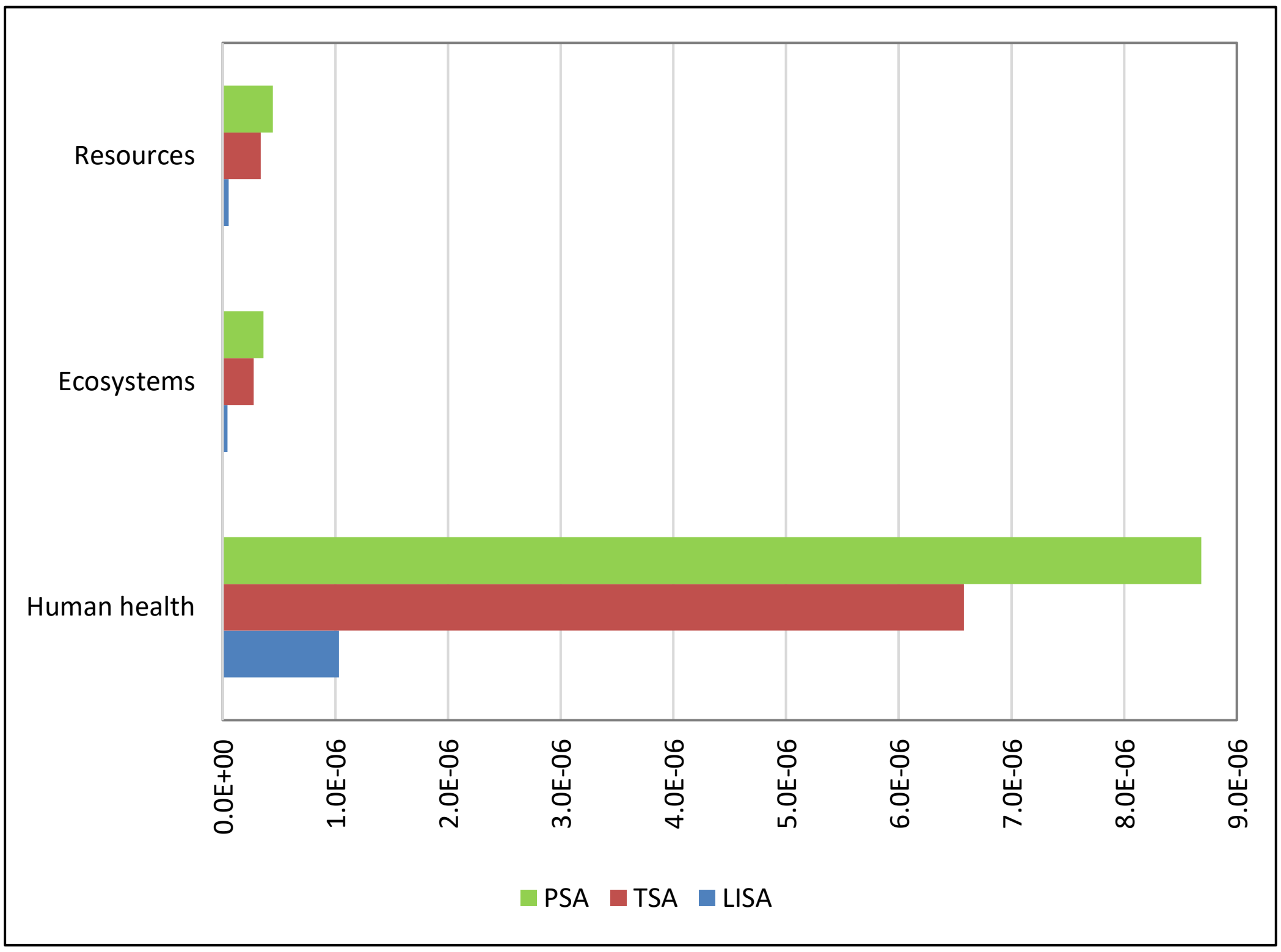

Figure S33. Comparison of endpoint environmental impacts from the operation and regeneration energy consumption of LISA, TSA, and PSA.

At the endpoint level, the normalisation results exhibit the same overall trend as observed in the midpoint analysis, thereby reinforcing the robustness of the conclusions drawn. As shown in **Figure S33** and **Table S6**, PSA results in the highest damage across all three endpoint categories, which are human health, ecosystems, and resources, followed by TSA, while LISA consistently demonstrates the lowest endpoint impacts. Among these categories, damage to human health dominates the overall endpoint profile, reflecting the aggregation of multiple energy-sensitive midpoint indicators, including human toxicity, particulate matter formation, ionizing radiation, and global warming.

Collectively, these results highlight that regeneration energy consumption plays a central role in determining the environmental sustainability of adsorption-driven CO_2 capture technologies. A high working capacity alone does not ensure favourable environmental performance if regeneration

energy is excessive. Conversely, adsorbents with lower capacities may still achieve superior environmental outcomes if their energy requirements are minimal. Based on the present analysis, LISA offers clear advantages in terms of operational energy efficiency and corresponding environmental impact, underscoring its potential as a more sustainable route for low-energy carbon capture.

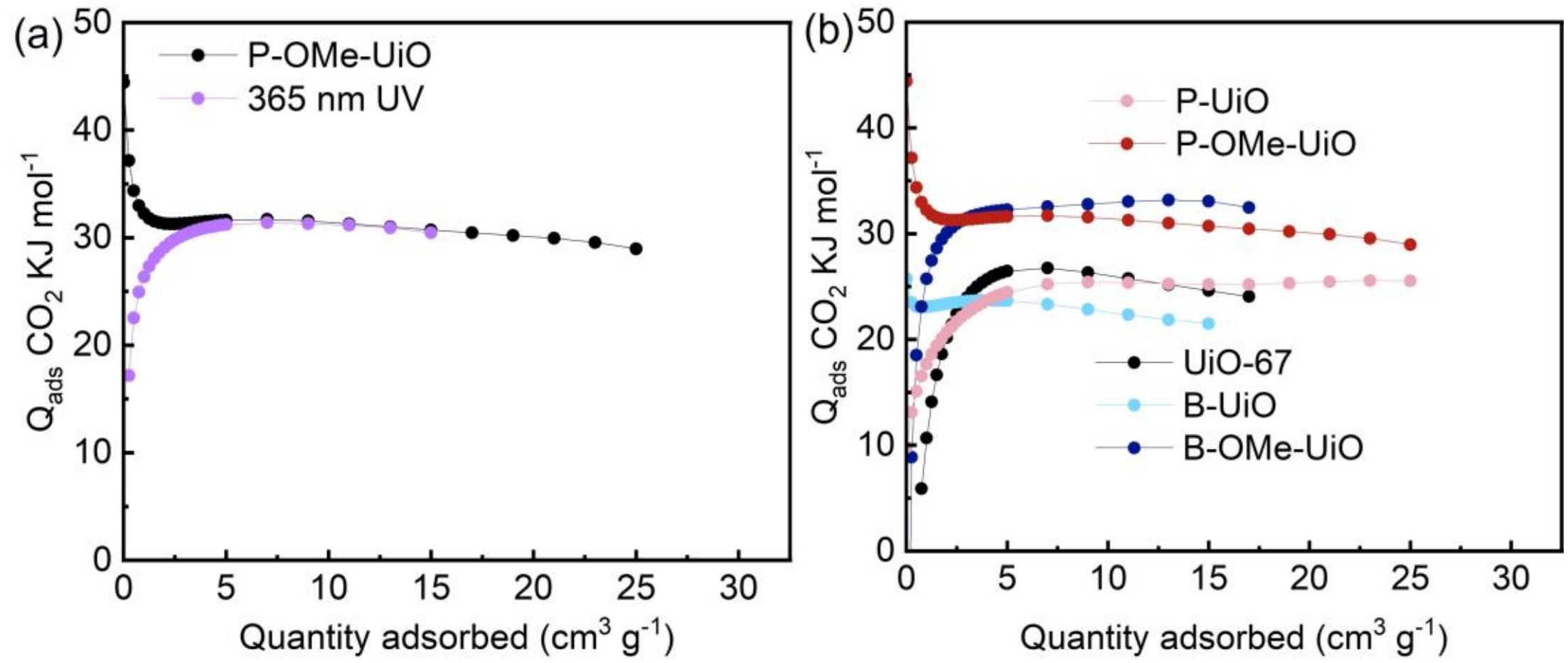

Figure S34. (a) The isosteric heat of adsorption enthalpy (Q_{ads}) of P-OMe-UiO CO_2 decreased from 45 to 16 KJ mol-1 upon UV-irradiation. (b) The CO_2 adsorption heat of SP-UiOs before and after UV irradiation. As can be seen from the figure, the parent UiO-67 exhibits a lower heat of adsorption compared with the SP-UiOs at high adsorption region. This is consistent with the enhanced CO_2 adsorption performance after the introduction of SP moieties into parent UiO-67. Notably, P-OMe-UiO shows the highest adsorption heat, indicating the strongest interaction with CO_2 molecules, also in good agreement with its superior CO_2 uptake.

3. Supplementary Tables

Table S1. The FWHM of the primary peak of SP-UiOs, and the calculated crystallite sizes via the Scherrer equation (D = Kλ/βcosθ)

Material	FWHM (°)	FWHM (rad)	Crystallite size D (nm)
P-UiO	0.406	0.00709	20.5
P-OMe-UiO	0.417	0.00728	19.9
B-UiO	1.748	0.03051	4.8
B-OMe-UiO	1.768	0.03086	4.7

Table S2. BET surface areas, pore volumes and CO_2 uptake capacities of different SP-UiOs and their changes upon UV and visible light irradiation.

**MOF**	**BET surface area (m^2 g^{-1})**	**UV △ (%)**	**Pore volume (cm^3 g^{-1})**	**UV△ (%)**	**CO_2 uptake (mmol g^{-1})**		**Working capacity (mmol g^{-1})**
					**273 K**	**UV△ (%)**	
UiO-67	1576	0	0.68	0	1.3	0	0
P-UiO	1622	19%🡻	0.69	18%🡻	2.2	28%🡻	0.6🡻
P-OMe-UiO	1632	12%🡻	0.69	11%🡻	2.7	40%🡻	1.1🡻
B-UiO	765	18%🡻	0.32	12%🡻	2.0	27%🡻	0.5🡻
B-OMe-UiO	664	16%🡻	0.29	11%🡻	1.8	30%🡻	0.5🡻

Table S3 Comparison of CO_2 working capacities of photo responsive MOFs reported in literature. AB = azobenzene, DET = diarylethene

	Photo-responsive moiety	Incorporation method	CO_2 uptake mmol/g	CO_2 working capacity	Cycles	Ref
F-azo-MIL-53(Al)	AB	Side group	0.8 (273K, 0.035 P/P_0)	0.12 (273 K)	1	[17]
$[Zn_2(3,3'$-bpeab)$(oba)_2]\cdot$DMF	AB	backbone	1.69 (298 K)	0.25 (298 K)	1	[18]
^{Azo}MOF	AB	Side group	19 (195K)	3.7 (195K)	1	[19]
$Zn_2(bdc)_2(LO)]_n$	DET	backbone	6.1 (195 K)	1.2 (195 K)	1	[20]
$Co_2L_2(AzoD)_2\cdot 2$DMF	AB	backbone	0.9 (273 K)	0.2 (273 K)	1	[21]
ECUT-30a	AB and DET		1.3 (273K)	0.4 (273K)	1	[22]
JUC-62	AB	backbone	2.1 (298 K)	0.7 (298 K)	1	[23]
Azo-UiO-66	AB	Side group	0.76 (298 K)	0.3 (298 K)	1	[24]
Azo-DMOF-1	AB	Side group	1.8 (298 K)	0.6 (298 K)	1	[25]
Zn(AzDC)(4,4'-BPE)$_{0.5}$	AB	backbone	1.3 (303K)	0.6 (303 K)	1	[2]
U-mazo	AB	Side group	0.83 (273K)	0.4 (273 K)	3	[2]
ECUT-15	AB	backbone	0.3 (298K)	0.1 (298 K)	1	[26]
T(7.5)/U-azo	AB	Side group	1.8 (273K)	0.8 (273 K)	3	[27]
PCN-250	AB	backbone	3.8 (298 K)	2.2 (298 K)	5	[28]
PCN-123	AB	Side group	1.0 (295K)	0.5 (295 K)	2	[29]
$PCTF_2$	AB	Side group	1.94 (273k)	0.4 (273 K)	3	[30]
P-OMe-UiO	SP	Linker	2.4 (273k)<br>1.03 (298 K)	1.1 (273 K)<br>0.4 (298 K)	6	This work

2.1. Supplementary Tables for LCA results

Table S4. CO_2 working capacities and energy consumptions for LISA of P-OMe-UiO in this work, TSA , and PSA[12].

Adsorption Type	Condition	CO_2 Working Capacity	Energy Consumption
**LISA**	298 K, 1 bar	0.38 mmol/g	60 J/cycle
**TSA**	298 K, 1 bar	1.03 mmol/g	1.67 kJ/cycle
	273K, 1 bar	2.7 mmol/g	
**PSA[12]**	298 K, 30 bar	16.2 mmol/g	20 kJ/cycle
	298 K, 1 bar	1 mmol/g	

Table S5. Environmental impacts of energy consumption for LISA in this work, TSA, and PSA in normalization across different midpoint impact categories.

**Impact category**	**LISA**	**TSA**	**PSA**
Global warming	1.67153E-06	1.06E-05	1.41E-05
Stratospheric ozone depletion	1.23232E-07	7.85E-07	1.04E-06
Ionizing radiation	1.74383E-05	0.000111	0.000147
Ozone formation, Human health	1.18459E-06	7.55E-06	9.96E-06
Fine particulate matter formation	4.03393E-07	2.57E-06	3.39E-06
Ozone formation, Terrestrial ecosystems	1.42275E-06	9.06E-06	1.2E-05
Terrestrial acidification	7.15404E-07	4.56E-06	6.01E-06
Freshwater eutrophication	3.58704E-06	2.28E-05	3.02E-05
Marine eutrophication	5.91134E-08	3.77E-07	4.97E-07
Terrestrial ecotoxicity	6.02773E-06	3.84E-05	5.07E-05
Freshwater ecotoxicity	6.58259E-05	0.000419	0.000553
Marine ecotoxicity	4.76221E-05	0.000303	0.0004
Human carcinogenic toxicity	4.98348E-05	0.000317	0.000419
Human non-carcinogenic toxicity	5.57297E-07	3.55E-06	4.69E-06
Land use	2.29164E-07	1.46E-06	1.93E-06
Mineral resource scarcity	5.14513E-10	3.28E-09	4.33E-09
Fossil resource scarcity	4.52782E-06	2.88E-05	3.81E-05
Water consumption	2.76338E-07	1.76E-06	2.32E-06

Table S6. Environmental impacts of energy consumption for LISA, TSA, and PSA in normalization across different endpoint impact categories.

**Impact category**	**LISA**	**TSA**	**PSA**
Human health	1.03E-06	6.58E-06	8.68E-06
Ecosystems	4.31E-08	2.74E-07	3.62E-07
Resources	5.32E-08	3.39E-07	4.48E-07

Table S7. The CO_2 binding energies results simulated based on different modelling SBUs structures of SP-UiO.

MOF SBUs	Complex EE (hartree)	Ligand EE (hartree)	CO_2 Binding Energy (hartree)	CO_2 Binding Energy (eV)
P-UiO	-4874.62	-4686.15	-0.007	-0.20
P-UiO (MC)	-5798.14	-5609.66	-0.017	-0.48
P-OMe-UiO	-5450.78	-5262.30	-0.011	-0.32
P-OMe-UiO (MC)	-5681.62	-5493.14	-0.016	-0.45
B-UiO	-5297.64	-5109.16	-0.008	-0.23
B-UiO (MC)	-5759.03	-5570.56	-0.009	-0.26
B-OMe-UiO	-5448.97	-5260.49	-0.015	-0.41
B-OMe-UiO (MC)	-5910.32	-5721.84	-0.007	-0.20

Table S8. Periodic DFT-calculated CO_2 binding energies (kJ mol^{-1}) for SP and MC states of SP-UiO MOFs and the parent defective UiO-67 framework.

Material	SP state CO_2 binding energy (kJ mol^{-1})	MC state CO_2 binding energy (kJ mol^{-1})	ΔE MC - SP (kJ mol^{-1})
Defective UiO-67	-34.58	--	--
P-OMe-UiO	-37.49	-35.73	+1.76
P-UiO	-33.66	-30.42	+3.24
B-UiO	-39.24	-37.53	+1.71
B-OMe-UiO	-37.65	-35.58	+2.07

ΔE = E(MC state) - E(SP state). Positive ΔE indicates weaker CO_2 binding in the MC state. — indicates not applicable (no photoswitchable unit present).

4. Supplementary References

[1] A. L. Myers, J. M. Prausnitz, *AIChE J.* **2004**, *11*, 121–127.

[2] R. Lyndon, K. Konstas, B. P. Ladewig, P. D. Southon, P. C. Kepert, M. R. Hill, *Angew Chem Int Ed Engl* **2013**, *52*, 3695–3698.

[3] A. Liu, G. Lu, X. Fan, C. H. Lau, *Journal of Materials Chemistry A* **2023**, *11*, 20559–20567.

[4] S. Øien, D. Wragg, H. Reinsch, S. Svelle, S. Bordiga, C. Lamberti, K. P. Lillerud, *Crystal Growth & Design* **2014**, *14*, 5370–5372.

[5] BIOVIA, *Dassault Systèmes, Material Studio, San Diego: Dassault Systèmes* **2019**.

[6] A. K. Rappi$, C. J. Casewit, K. S. Colwell, W. A. Goddard III, W. M. Skid, *J. Am. Chem. SOC* **1992**, *114*, 10024–10039.

[7] S. Grimme, S. Ehrlich, L. Goerigk, *J. Comput. Chem.* **2011**, *32*, 1456–1465.

[8] J. P. Perdew, K. Burke, M. Ernzerhof, *Phys. Rev. Lett.* **1996**, *77*, 3865–3868.

[9] G. Kresse, D. Joubert, *Phys. Rev. B* **1999**, *59*, 1758–1775.

[10] *Gaussian 09, M. J. Frisch, G. W. Trucks, H. B. Schlegel, G. E. Scuseria, M. A. Robb, J. R. Cheeseman, G. Scalmani, V. Barone, G. A. Petersson, H. Nakatsuji, X. Li, M. Caricato, A. Marenich, J. Bloino, B. G. Janesko, R. Gomperts, B. Mennucci, H. P. Hratchian, J. V. Ortiz, A. F. Izmaylov, J. L. Sonnenberg, D. Williams-Young, F. Ding, F. Lipparini, F. Egidi, J. Goings, B. Peng, A. Petrone, T. Henderson, D. Ranasinghe, V. G. Zakrzewski, J. Gao, N. Rega, G. Zheng, W. Liang, M. Hada, M. Ehara, K. Toyota, R. Fukuda, J. Hasegawa, M. Ishida, T. Nakajima, Y. Honda, O. Kitao, H. Nakai, T. Vreven, K. Throssell, J. A. Montgomery, Jr., J. E. Peralta, F. Ogliaro, M. Bearpark, J. J. Heyd, E. Brothers, K. N. Kudin, V. N. Staroverov, T. Keith, R. Kobayashi, J. Normand, K. Raghavachari, A. Rendell, J. C. Burant, S. S. Iyengar, J. Tomasi, M.*

Cossi, J. M. Millam, M. Klene, C. Adamo, R. Cammi, J. W. Ochterski, R. L. Martin, K. Morokuma, O. Farkas, J. B. Foresman, and D. J. Fox, Gaussian, Inc., Wallingford CT, **2016**.

[11] L. Sarkisov, J. Kim, *Chem. Eng. Sci.* **2015**, *121*, 322–330.

[12] A.-M. Banu, D. Friedrich, S. Brandani, T. Düren, *Industrial & Engineering Chemistry Research* **2013**, *52*, 9946–9957.

[13] K. C. Park, C. R. Martin, G. A. Leith, G. C. Thaggard, G. R. Wilson, B. J. Yarbrough, B. K. P. Maldeni Kankanamalage, P. Kittikhunnatham, A. Mathur, I. Jatoi, M. A. Manzi, J. Lim, I. Lehman-Andino, A. Hernandez-Jimenez, J. W. Amoroso, D. P. DiPrete, Y. Liu, J. Schaeperkoetter, S. T. Misture, S. R. Phillpot, S. Hu, Y. Li, A. Leydier, V. Proust, A. Grandjean, M. D. Smith, N. B. Shustova, *J Am Chem Soc* **2022**, *144*, 16139–16149.

[14] A. Kazemi, F. Moghadaskhou, M. A. Pordsari, F. Manteghi, A. Tadjarodi, A. Ghaemi, *Sci Rep* **2023**, *13*, 19891.

[15] T. N. M. Tran, J. Kim, T. K. Vo, *Inorg. Chem. Commun.* **2023**, *153*.

[16] aJ. Liu, J. Wang, J. Cardinal, *Renewable and Sustainable Energy Reviews* **2022**, *161*, 112317; bI. C. Tsai, *Energy Policy* **2024**, *188*, 114065.

[17] S. Castellanos, A. Goulet-Hanssens, F. Zhao, A. Dikhtiarenko, A. Pustovarenko, S. Hecht, J. Gascon, F. Kapteijn, D. Bleger, *Chemistry* **2016**, *22*, 746–752.

[18] W. C. Song, X. Z. Cui, Z. Y. Liu, E. C. Yang, X. J. Zhao, *Sci Rep* **2016**, *6*, 34870.

[19] H. Huang, H. Sato, T. Aida, *J Am Chem Soc* **2017**, *139*, 8784–8787.

[20] Y. Zheng, H. Sato, P. Wu, H. J. Jeon, R. Matsuda, S. Kitagawa, *Nat Commun* **2017**, *8*, 100.

[21] L.-L. Dang, X.-J. Zhang, L. Zhang, J.-Q. Li, F. Luo, X.-F. Feng, *J. Coord. Chem.* **2016**, *69*, 1179–1187.

[22] C. B. Fan, Z. Q. Liu, L. L. Gong, A. M. Zheng, L. Zhang, C. S. Yan, H. Q. Wu, X. F. Feng, F. Luo, *Chem Commun (Camb)* **2017**, *53*, 763–766.

[23] N. Prasetya, B. P. Ladewig, *Sci Rep* **2017**, *7*, 13355.

[24] N. Prasetya, B. C. Donose, B. P. Ladewig, *Journal of Materials Chemistry A* **2018**, *6*, 16390–16402.

[25] N. Prasetya, B. P. Ladewig, *ACS Appl Mater Interfaces* **2018**, *10*, 34291–34301.

[26] L. Gong le, X. F. Feng, F. Luo, *Inorg Chem* **2015**, *54*, 11587–11589.

[27] Y. Jiang, P. Tan, S. C. Qi, X. Q. Liu, J. H. Yan, F. Fan, L. B. Sun, *Angew Chem Int Ed Engl* **2019**, *58*, 6600–6604.

[28] H. Li, M. R. Martinez, Z. Perry, H. C. Zhou, P. Falcaro, C. Doblin, S. Lim, A. J. Hill, B. Halstead, M. R. Hill, *Chemistry* **2016**, *22*, 11176–11179.

[29] J. Park, D. Yuan, K. T. Pham, J. R. Li, A. Yakovenko, H. C. Zhou, *J Am Chem Soc* **2012**, *134*, 99–102.

[30] Q. Huang, Z. Zhan, R. Sun, J. Mu, B. Tan, C. Wu, *Angew Chem Int Ed Engl* **2023**, *62*, e202305500.
