# Supplementary material for: Photoreversible defect complexation dynamically modulates guest adsorption in metal-organic frameworks
Source: Nat Commun. 2026 Oct 15;17:11629. doi: 10.1038/s41467-026-78352-9 (PMC13754871; doi:10.1038/s41467-026-78352-9)
Supplement: Supplementary file 2 [file 41467_2026_78352_MOESM2_ESM.pdf]

nature portfolio

Peer Review File

Photoreversible Defect Complexation Dynamically Modulates Guest Adsorption in Metal-Organic Frameworks

Corresponding Author: Dr Qi Huang

This file contains all reviewer reports in order by version, followed by all author rebuttals in order by version.

Version 0:

Reviewer comments:

Reviewer #1

(Remarks to the Author)
The contribution by Lau et al. presents significant work on modulating gas adsorption in Metal-Organic Frameworks (MOFs). By functionalizing defective UiO-67 with light-responsive spiropyran derivatives, the authors propose a reversible light-induced spiropyran–merocyanine (SP–MC) isomerization to modulate CO_2 adsorption. The authors present an interesting concept of "photo-reversible defect complexation" as a mechanism distinct from conventional pore-blocking or polarity-driven effects. While the conceptual framework is potentially significant for the development of smart adsorbent materials, several critical inconsistencies in the characterization and the resulting mechanistic claims must be addressed before publication can be considered.
Issues to address before publication:
1) The authors mentioned, "This mechanism delivers an 83% enhancement in photo-modulated CO_2 working capacity, reaching 1.1 mmol g^{-1}". The presentation of the 83% enhancement requires clarification, since the comparison between P-OMe and P-UiO involves structurally distinct baselines that may obscure the true origin of the improvement.
Furthermore, the authors state in the Introduction that the "MC-Zr complexation reach 1.1 mmol g-1, double of those of non-defect switchable analogues." However, the manuscript fails to explicitly define the "non-defect switchable analogues" used for this comparison. The authors must clearly identify the control material; is it a non-defective UiO-67, or a different framework entirely?
2) The authors attribute the enhanced adsorption in the grafted materials to the introduction of polar functional groups (specifically the methoxy and nitro groups) on the spiropyran ligand. However, the Merocyanine (MC) isomer produced under UV irradiation is characterized by a higher dipole moment and greater electronic density on the phenolate oxygen than the closed Spiropyran (SP) form. Theoretically, this should strengthen the electrostatic interactions with CO2 and increase the adsorption capacity. The authors must justify how blocking a metal site can energetically outweigh the presence of a more polar functional group in the same pore.
3) The authors claim that the BET surface area increases from 1576 m^2/g to over 1620 m^2/g by grafting bulky organic ligands 1 and 2. Given that the introduction of ligands adds substantial mass (up to 12.7 wt.%) and occupies existing pore volume, a decrease in surface area is the expected physical outcome. The authors should provide an explanation for this increase. Furthermore, the argument that alkyl chains reduce steric hindrance to enhance crystallinity lacks quantitative evidence.
4) The authors state that ex-situ XPS shift of the Zr 3d5/2 binding energy of approximately 0.07 eV is proof of chemical bonding. In the context of standard XPS instrumentation, such a marginal shift often lies within the margin of error or at the instrument's resolution limit. The authors must justify the statistical significance of this value and clarify whether that might account for potential charging effects or light-induced thermal shifts during measurement.
5) The manuscript's claim toward the material's durability is not sufficiently supported by the limited cycling data provided. To substantiate the claim that this framework is truly robust and possesses practical utility, the authors must provide extended cycling experiments to demonstrate that the crystallinity stabilizes rather than continues to degrade
6) The authors have reported the working capacity and gas adsorption isotherms exclusively at 273 K. It is unclear why this specific temperature was chosen for a material intended for carbon capture applications.
Overall, the science disclosed in this manuscript is appropriate for the broad readership of Nature Communications. This work represents a clever application of coordination chemistry within a porous framework, likely to inspire further efforts to develop 'smart' adsorbents. However, prior to publication, the authors must address the significant concerns delineated above to ensure the mechanistic claims are as robust as the conceptual framework.

Reviewer #2

(Remarks to the Author)
In this manuscript, the authors report a conceptually novel mechanism of photo-reversible defect complexation in which spiropyran (SP) photoswitches grafted onto open Zr sites in defective UiO-67 MOFs enabling reversible coordination of the MC phenolate oxygen with adjacent open Zr sites. This directly modulates active binding site availability rather than relying on conventional pore geometry or polarity changes. The resulting CO2 working capacity of 1.1 mmol g-1 represents an 83% enhancement over non-defect-switchable analogues, and the generality of the mechanism is demonstrated across both gas-phase and solution-phase cargo. The work is supported by a comprehensive and well-integrated characterization method including ex-situ, in-situ, and NAP-XPS, DFT simulations, lifecycle assessment, and breakthrough experiments. The manuscript is well-written and the central hypothesis is clearly articulated. I consider this work to be a significant conceptual advance in the field of stimuli-responsive porous materials and recommend publication in Nature Communications subject to revision addressing the points below.
1. On page 12, the text states "P-UiO showed significantly lower Na+ uptake of only ~0.07 mmol g^{-1}" but the subject of the comparison should be P-OMe-UiO, not P-UiO. This appears to be a typographical error that inverts the intended comparison and should be corrected carefully as it affects the logical flow of the argument. There are also some other typing errors that need to be revised: page 18: "This apparent paradox is resolve by" should be "resolved". page 17 contains a typographical error ("pore volume loos" should be "pore volume loss")

2. The lifecycle assessment (LCA) results are cited as supporting evidence for the low environmental impact of this approach, yet the LCA methodology, system boundaries, and key assumptions are relegated to the Supporting Information. Given that the LCA is presented as a key differentiator of this work, the authors should include at least a brief description of the LCA methodology and its key assumptions in the main text, alongside a statement of any limitations. This is particularly important given that the 60 J energy figure cited in the main text differs from the 240 J figure stated in the Table of Contents entry, which requires clarification.

3. The caption for Figure 4 does not match the corresponding image. Please either insert the correct figure related to metal adsorption experiments or modify the caption accordingly.

4. The authors present impressive CO2/N2 selectivity data (IAST = 500) for P-OMe-UiO under dark conditions in Figure 5e, which is a key strength of this material. However, a critical aspect requires further investigation. The proposed photo-reversible defect complexation mechanism demonstrates that UV irradiation substantially reduces the binding affinity of open Zr sites for CO2, which would logically result in diminished CO2/N2 selectivity under illumination. To fully validate the practical viability of this photoswitchable approach, the authors should provide supplementary discussion on the IAST mixed-gas selectivity after SP to MC isomerization and explain whether this reduced selectivity would affect the purity in practical gas separation, for instance, whether significant amounts of N2 would be co-desorbed during the desorption phase.

5. The manuscript reports CO2 adsorption enthalpy decreasing from 45 to 16 kJ mol-1 upon UV irradiation, which is crucial evidence for the LISA mechanism. However, the calculation methodology for these isosteric heat (Qst) values requires clarification. The authors should specify in the Supporting Information or methods section: the calculation approach used (e.g., Clausius-Clapeyron analysis), temperature conditions. This documentation is essential for reproducibility and validation of these key energetic parameters.

6. The authors report IAST selectivity at 298 K but do not provide the CO_2 working capacity of the lead material P-OMe-UiO at this temperature. Since working capacity is the central performance metric of this study, it is recommended that the authors include the photo-modulated CO_2 working capacity of P-OMe-UiO at 298 K for a brief comparison, which would substantially enhance the demonstration of its practical application potential.

Version 1:

Reviewer comments:

Reviewer #1

(Remarks to the Author)
The authors have satisfactorily addressed all of my comments and concerns. I have no further comments and support publication of the manuscript in its revised form.

Reviewer #2

(Remarks to the Author)
The authors have satisfactorily addressed the concerns I raised throughout the review process. I therefore recommend acceptance of this manuscript for publication in Nature Communications with no further revisions required.

Open Access This Peer Review File is licensed under a Creative Commons Attribution 4.0 International License, which permits use, sharing, adaptation, distribution and reproduction in any medium or format, as long as you give appropriate credit to the original author(s) and the source, provide a link to the Creative Commons license, and indicate if changes were made.
In cases where reviewers are anonymous, credit should be given to 'Anonymous Referee' and the source.
The images or other third party material in this Peer Review File are included in the article's Creative Commons license, unless indicated otherwise in a credit line to the material. If material is not included in the article's Creative Commons license and your intended use is not permitted by statutory regulation or exceeds the permitted use, you will need to obtain permission directly from the copyright holder.
To view a copy of this license, visit https://creativecommons.org/licenses/by/4.0/

Response to Reviewer Comments for Manuscript # NCOMMS-26-024638

We thank both reviewers for taking time to review this manuscript and provide comments to improve the quality of this work. We have addressed these comments (shown in blue) and revised the manuscript (in red) as follows:

Reviewer 1

The contribution by Lau et al. presents significant work on modulating gas adsorption in Metal-Organic Frameworks (MOFs). By functionalizing defective UiO-67 with light-responsive spiropyran derivatives, the authors propose a reversible light-induced spiropyran-merocyanine (SP-MC) isomerization to modulate CO_2 adsorption. The authors present an interesting concept of "photo-reversible defect complexation" as a mechanism distinct from conventional pore-blocking or polarity-driven effects. While the conceptual framework is potentially significant for the development of smart adsorbent materials, several critical inconsistencies in the characterization and the resulting mechanistic claims must be addressed before publication can be considered. Issues to address before publication:

1) The authors mentioned, "This mechanism delivers an 83% enhancement in photo-modulated CO_2 working capacity, reaching 1.1 mmol g^{-1}". The presentation of the 83% enhancement requires clarification, since the comparison between P-OMe and P-UiO involves structurally distinct baselines that may obscure the true origin of the improvement.

Furthermore, the authors state in the Introduction that the "MC-Zr complexation reach 1.1 mmol g^{-1}, double of those of non-defect switchable analogues." However, the manuscript fails to explicitly define the "non-defect switchable analogues" used for this comparison. The authors must clearly identify the control material; is it a non-defective UiO-67, or a different framework entirely?

Response: We clarify that P-UiO serves as the primary control for P-OMe-UiO. These two materials are structurally analogous, differing only in the presence or absence of the methoxy (OMe) substituent beside the phenyl oxygen. The 83% enhancement directly reflects this difference: P-OMe-UiO achieves 1.1 mmol g^{-1} versus 0.6 mmol g^{-1} for P-UiO.

Figure 5g shows that there is no correlation between photo-modulated CO_2 working capacities against UV-induced pore volume changes across all four SP-UiO materials, ruling out pore as a governing factor. Figure 5h instead reveals a direct linear correlation between working capacity and UV-modulated Zr $3d_{5/2}$ binding energy shifts.

P-OMe-UiO uniquely exhibits MC-Zr complexation, the largest binding energy shift and highest working capacity; P-UiO shows minimal shift and lower working capacity. This identifies MC-Zr complexation as the mechanistic determinant, with the OMe group as the critical structural feature that activates this improvement.

The parent defective UiO-67 (without SP) serves as a separate blank control to confirm that photo-responsive CO_2 switching originates from SP rather than the MOF framework itself. As such, we have revised the Abstract and Introduction with the following red texts:

Abstract Page 3 Line 8: This mechanism delivers an 83% enhancement in photo-modulated CO_2 working capacity, reaching 1.1 mmol g^{-1}, nearly double the chemical structurally analogue of P-UiO that does not enable photo-reversible MC-defect switching.

Introduction Page 5 Line 1: At 273 K, the CO_2 working capacities of MOFs displaying photo-reversible MC-Zr complexation reach 1.1 mmol g^{-1}, 83% higher than the structurally analogous P-UiO. Experimental correlation analysis confirms that MC-Zr complexation underpins this enhancement, with

the methoxy substituent in P-OMe-UiO identified as the critical structural feature that activates it.

2) The authors attribute the enhanced adsorption in the grafted materials to the introduction of polar functional groups (specifically the methoxy and nitro groups) on the spiropyran ligand. However, the Merocyanine (MC) isomer produced under UV irradiation is characterized by a higher dipole moment and greater electronic density on the phenolate oxygen than the closed Spiropyran (SP) form. Theoretically, this should strengthen the electrostatic interactions with CO_2 and increase the adsorption capacity. The authors must justify how blocking a metal site can energetically outweigh the presence of a more polar functional group in the same pore.

Response: Periodic DFT calculations of CO_2 binding energies across all SP-UiO materials show how blocking a metal site energetically outweighs the presence of a more polar functional group in the same pore. These values are summarised in Table S8 below and included in Page 44 of the revised SI.

Table S8. Periodic DFT-calculated CO_2 binding energies (kJ mol^{-1}) for SP and MC states of SP-UiO MOFs and the parent defective UiO-67 framework.

**Material**	**SP state CO_2 binding energy (kJ mol^{-1})**	**MC state CO_2 binding energy (kJ mol^{-1})**	**ΔE MC - SP (kJ mol^{-1})**
Defective UiO-67	-34.58	--	--
P-OMe-UiO	-37.49	-35.73	+1.76
P-UiO	-33.66	-30.42	+3.24
B-UiO	-39.24	-37.53	+1.71
B-OMe-UiO	-37.65	-35.58	+2.07

ΔE = E(MC state) - E(SP state). Positive ΔE indicates weaker CO_2 binding in the MC state. — indicates not applicable (no photoswitchable unit present).

In the SP state, P-OMe-UiO exhibits a CO_2 binding energy of -37.49 kJ mol^{-1}, reflecting strong OMS-mediated binding at open Zr sites. Upon UV-induced MC-Zr complexation, this weakens to -35.73 kJ mol^{-1}. This indicates that MC-Zr complexation substantially reduces access to the high-energy adsorption sites. In free MC, the high dipole moment (+14 D) arises from the zwitterionic charge-separated state and greater electronic density on the phenolate oxygen. Upon MC-Zr complexation, the phenolate oxygen coordinates directly to the open Zr site, simultaneously blocking the high-energy OMS and neutralising its electronic density on the phenolate oxygen. Both effects reduce CO_2 affinity under UV irradiation in P-OMe-UiO.

For B-UiO and B-OMe-UiO, periodic calculations yield weaker binding in the MC state (ΔE = +1.71 and +2.07 kJ mol^{-1} respectively). This is attributed to steric restriction by the extended MC form (around 12 Å vs 8 Å for SP), which reduces BET surface areas by >50%, leaving the phenolate oxygen geometrically inaccessible for CO_2 interaction. We therefore conclude that CO_2 adsorption capacity in SP-UiO MOFs cannot be predicted from molecular dipole moments alone: the three-dimensional pore architecture and the site-accessibility hierarchy consistently dominate.

The following text has been added to the Results and Discussion section on Page 18:

Results and Discussion Page 18 Line 19: This energetic dominance of OMS blocking over MC polarity arises from two compounding effects. First, MC-Zr complexation directly engages the phenolate oxygen in a Zr-O coordination bond, simultaneously blocking the OMS and neutralising the intrinsic polarity advantage of MC for CO_2 interaction in P-OMe-UiO. Second, periodic DFT calculations across all four SP-UiO materials consistently yield lower CO_2 binding energies in the MC state than the SP state (Table S8), confirming that the confined pore environment systematically prevents MC's higher molecular dipole moment from translating into enhanced CO_2 binding affinity. Together, these effects establish OMS blocking as the energetically dominant mechanism governing CO_2 working capacity in P-OMe-UiO.

3) The authors claim that the BET surface area increases from 1576 m^2/g to over 1620 m^2/g by grafting bulky organic ligands 1 and 2. Given that the introduction of ligands adds substantial mass (up to 12.7 wt.%) and occupies existing pore volume, a decrease in surface area is the expected physical outcome. The authors should provide an explanation for this increase. Furthermore, the argument that alkyl chains reduce steric hindrance to enhance crystallinity lacks quantitative evidence.

Response: We first quantified the framework ordering across all four SP-UiO materials by Scherrer analysis of background-subtracted PXRD data (Figure 1b) using the FWHM of the primary peak at $2\theta = 6.6°$ ($D = K\lambda/\beta\cos\theta$). As shown in Table S1, P-UiO and P-OMe-UiO exhibit crystallite sizes of ~ 20 nm, roughly four times larger than B-UiO and B-OMe-UiO. We attribute this to the conformational flexibility of the alkyl chain in ligands **1** and **2**, which relieves steric constraints at the grafting site and facilitates ordered coordination onto the Zr nodes. The rigid benzene ring in ligands **3** and **4**, by contrast, induces steric congestion at the defect site, restricting ordered assembly.

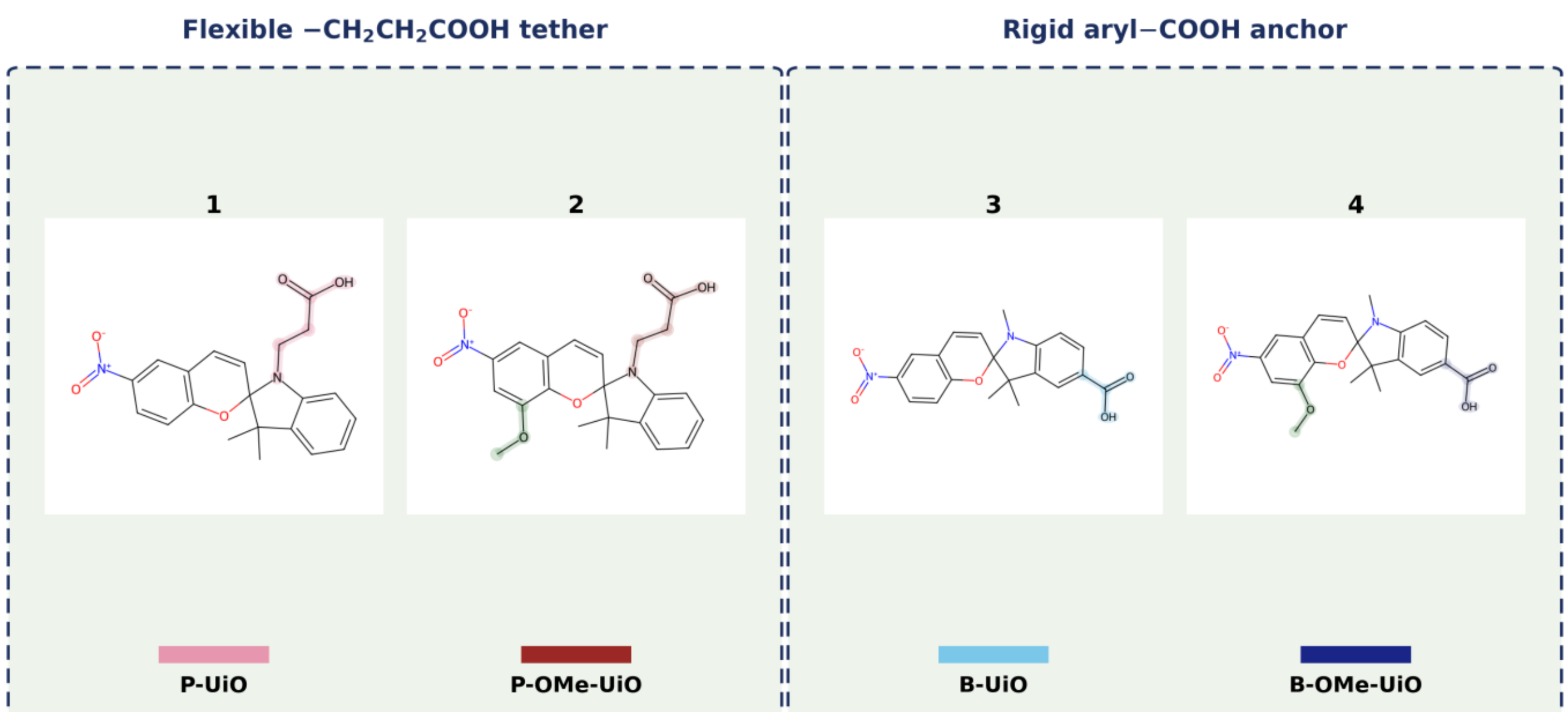

Figure S7. The chemical structures of 1 – 4 and the difference between the carboxyl chain.

Table S1. The FWHM of the primary peak of SP-UiOs, and the calculated crystallite sizes via the Scherrer equation ($D = K\lambda/\beta\cos\theta$)

Material	FWHM (°)	FWHM (rad)	Crystallite size D (nm)
P-UiO	0.406	0.00709	20.5
P-OMe-UiO	0.417	0.00728	19.9
B-UiO	1.748	0.03051	4.8
B-OMe-UiO	1.768	0.03086	4.7

The BET increase is not anomalous when the parent material, defective UiO-67, is considered. Defective UiO-67 contains missing-linker sites with coordinatively unsaturated Zr nodes, residual modulator molecules, and localized disorder.[1] Grafting ligands **1** and **2** produces two concurrent benefits that offset the added mass (⩽ 12.7 wt.%). First, the carboxylic acid groups displace residual modulator species at open Zr sites via solvent-assisted ligand exchange, reopening previously occluded pore channels. This is a well-precedented effect reported in literature: Shearer et al increased the BET surface area of benzoate-compensated defective UiO-66 from 1515 to 1903 $m^2\ g^{-1}$ by this approach[2], and Yuan et al. demonstrated that post-synthetic linker installation at missing-linker sites in PCN-700 increased H_2 uptake by 57% without sacrificing porosity[3]. Second, coordination of the ligands to open Zr nodes restores partial framework connectivity and reduces local disorder, evidenced directly by the narrower PXRD peak widths of P-UiO and P-OMe-UiO relative to defective UiO-67. Together, these effects increase the accessible, ordered pore surface area by more than the mass loading of grafted ligands.

Based on this response, we have revised the manuscript as follows:

Results and Discussion Page 6 Line 18: The P-series materials exhibit four times smaller FWHM of the primary diffraction peak at 2θ = 6.6° than the B-series, corresponding to four times larger crystallite sizes by Scherrer analysis (Table S1). This directly demonstrates that alkyl chain connectivity promotes significantly more ordered framework assembly than the rigid benzene-ring-tethered carboxylic acid in ligands **3** and **4**. The alkyl chains in ligands **1** and **2** could reduce steric hindrance and enable grafting onto defect sites in the UiO-67, enhancing the framework ordering of resultant MOFs.

Results and Discussion Page 7 Line 1: Grafting **1** and **2** onto missing-linker sites in defective UiO-67 thus slightly enhances rather than reduces porosity. Their carboxylic acid groups can displace residual modulator species at open Zr sites, reopening previously occluded pore channels.[1, 2, 3] Meanwhile coordination to the open Zr nodes restores partial framework connectivity and reduces local disorder. These are evidenced by the narrower PXRD peak widths of P-UiO and P-OMe-UiO relative to the parent framework.

4) The authors state that ex-situ XPS shift of the Zr $3d_{5/2}$ binding energy of approximately 0.07 eV is proof of chemical bonding. In the context of standard XPS instrumentation, such a marginal shift often lies within the margin of error or at the instrument's resolution limit. The authors must justify the statistical significance of this value and clarify whether that might account for potential charging effects or light-induced thermal shifts during measurement.

Response: The real-time detector operates in snapshot mode with a 0.02 eV step size and noise-limited error of ± 0.02 eV. This was well below the observed 0.07 eV Zr $3d_{5/2}$ shift, which was reproduced across multiple independent acquisitions; shifts of this magnitude are demonstrably resolvable in this configuration. A thermal origin is excluded by the low UV power at the sample (1.65 µW, no measurable temperature change, ± 0.1 °C): the 60-fold more powerful white-light source (0.1 mW) reverses rather

than amplifies the shift. Charging is likewise excluded: samples were grounded with flood-source charge suppression, and the O 1s and Zr $3d_{5/2}$ peaks shift in opposite directions — inconsistent with a charging artefact. Critically, this anti-correlated shift occurs exclusively in P-OMe-UiO and not in P-UiO, B-UiO, or B-OMe-UiO (Figure 3h), confirming that the observed shifts reflect genuine MC-Zr complexation.

To address the reviewer's comments, the following text has been added to the Supporting Information:

The real-time XPS is conducted with a custom, real-time detector in a snapshot mode across a 10 eV window, with a step size resolution of 0.02 eV. This allows us to accurately obtain information across the core levels quickly, which has a much greater level of accuracy than is typical of XPS, and other real-time XPS methods for measuring the exact shifts. The shifts observed in the XPS before and after UV exposure in the high-resolution core level spectra match the data provided by the in-situ real-time exposure. For the P-OMe-UiO sample, the shift after exposure to UV is approximately 0.07 eV, which matches the value seen in the core level spectra. The average error in the real-time spectra due to noise is ± 0.02 eV. Samples measured in UHV before and after UV exposure, and during real-time measurements, remain stationary and in position to ensure high accuracy. Sample measurements are repeated and show the same effect.

As shown in S. Astley et al.[4], binding energy shifts of small values can be detected as low as the values we observe and can arise due to a variety of factors. Importantly, even when thermally annealing the sample up to 600°C with gold alloys, shifts of only 0.06-0.07 eV are observed, demonstrating the instrument's sensitivity at this scale. The power of the tunable light source at the UV wavelength used was measured at 1.65 μW at the sample. At such low powers, shifting due to thermal effects is undetectable. Sample temperatures were monitored continuously during experiments via direct contact measurement of the mounting plates, and no thermal change was observed (within ±0.1°C accuracy) before, during, or after UV exposure. The white light source is considerably more powerful at 0.1 mW, yet the Zr 3d spectra return to the exact same energy as pre-UV conditions during white light illumination, and no temperature change was observed. This indicates that any shifts are entirely due to the molecular transition rather than thermal or charging effects.

Samples are mounted onto a grounded conductive plate to compensate for any intrinsic charging. A twin-anode flood source X-ray gun was used during measurements in the UHV system; these typically generate a large number of stray electrons which suppress potential charging effects in XPS. In cases where charging has been observed, peaks have been shifted with respect to charge referencing of the C 1s spectrum. If charging were the cause of the observed shifts, the O 1s would shift in the same direction as the Zr peak, and the peaks would not alternate between higher and lower kinetic energies. Charging would also be expected to produce effects across the full range of samples rather than specifically in P-OMe-UiO.

Astley S, et al. Identifying chemical and physical changes in wide-gap semiconductors using real-time and near ambient-pressure XPS. Faraday Discuss 236, 191-204 (2022).

5) The manuscript's claim toward the material's durability is not sufficiently supported by the limited cycling data provided. To substantiate the claim that this framework is truly robust and possesses practical utility, the authors must provide extended cycling experiments to demonstrate that the crystallinity stabilizes rather than continues to degrade

Response: We thank the reviewer for this important comment on cycling durability. We have performed extended cycling experiments up to six complete SP/MC switching cycles, with post-cycling PXRD and FT-IR characterization at SP_0, SP_4, and SP_6. These additional experimental data confirm that P-OMe-UiO retains meaningful photo-modulated CO_2 working capacity across all six cycles (0.74 – 1.07 mmol

g^{-1}, Figure 5c). A more pronounced decline beyond six cycles is accompanied by visible lightening of the sample. This is attributed to partial MC aggregate accumulation and irreversible photobleaching of the SP chromophore after ~ 15 h cumulative UV exposure. Even so, six-cycle performance still exceeds most photo-responsive porous materials reported to date (Table S3). Since photostability is critical for application, strategies including more photostable SP derivatives,[5] covalent immobilisation to suppress MC leaching,[6] and polymer-tethered SP systems[7] are promising routes to extend cycling range and are currently under investigation in our group.

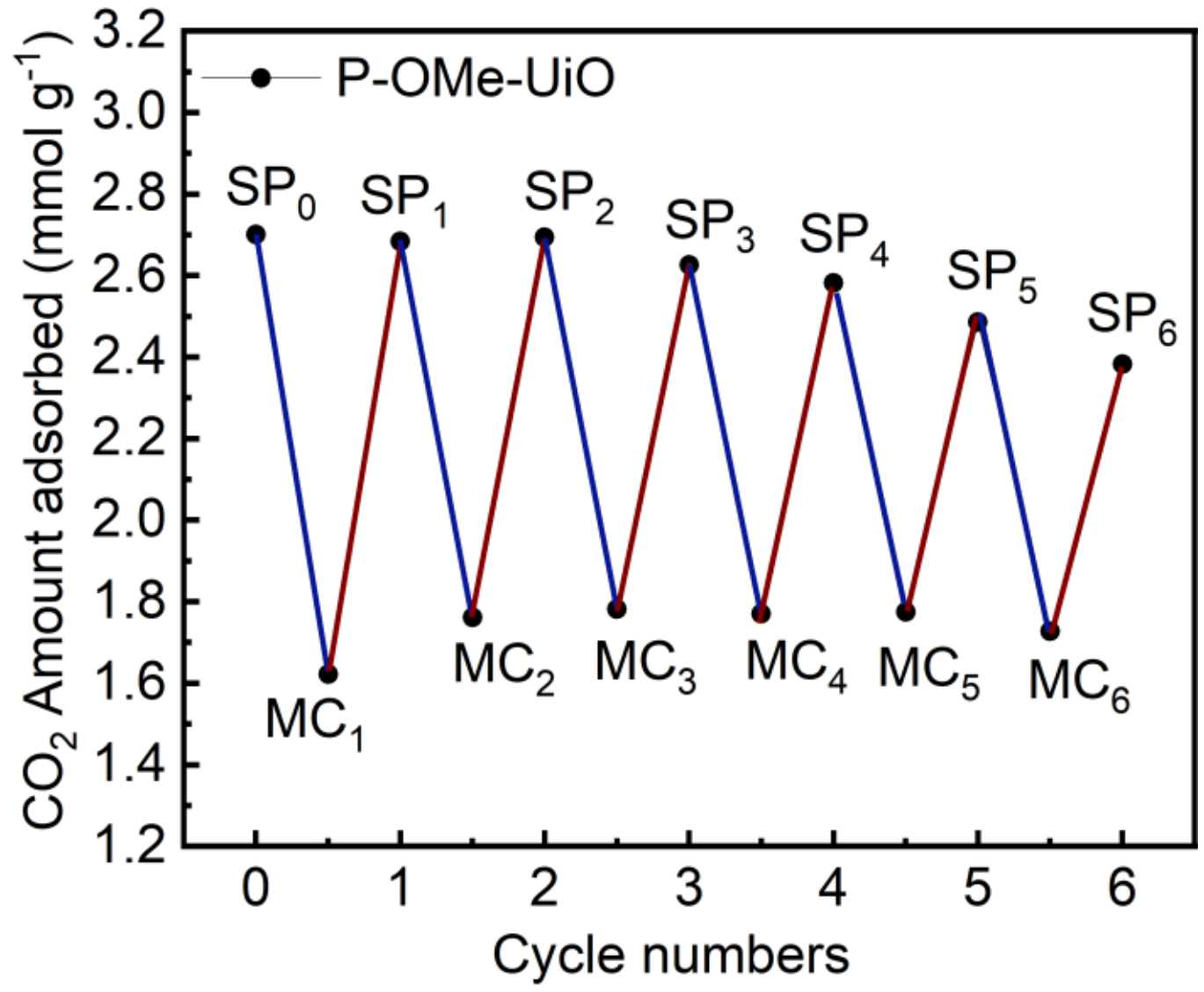

Figure 5c. CO_2 cycling adsorption performance of P-OMe-UiO over multiple photoswitching cycles.

Table S3 *Comparison of CO_2 working capacities of photo responsive MOFs reported in literature. AB = azobenzene, DET = diarylethene*

	**Photo-responsive moiety**	**Incorporation method**	**CO_2 uptake mmol/g**	**CO_2 working capacity**	**Cycles**	**Ref**
F-azo-MIL-53(Al)	AB	Side group	0.8 (273K, 0.035 P/P$_0$)	0.12 (273 K)	1	8
[Zn_2(3,3′-bpeab)$(oba)_2$]·DMF	AB	backbone	1.69 (298 K)	0.25 (298 K)	1	9
^{Azo}MOF	AB	Side group	19 (195K)	3.7 (195K)	1	10
$Zn_2(bdc)_2(LO)]_n$	DET	backbone	6.1 (195 K)	1.2 (195 K)	1	11
$Co_2L_2(AzoD)_2$·2DMF	AB	backbone	0.9 (273 K)	0.2 (273 K)	1	12
ECUT-30a	AB and DET		1.3 (273K)	0.4 (273K)	1	13
JUC-62	AB	backbone	2.1 (298 K)	0.7 (298 K)	1	14
Azo-UiO-66	AB	Side group	0.76 (298 K)	0.3 (298 K)	1	15

Azo-DMOF-1	AB	Side group	1.8 (298 K)	0.6 (298 K)	1	[16]
Zn(AzDC)(4,4'-BPE)$_{0.5}$	AB	backbone	1.3 (303K)	0.6 (303 K)	1	[17]
U-mazo	AB	Side group	0.83 (273K)	0.4 (273 K)	3	[17]
ECUT-15	AB	backbone	0.3 (298K)	0.1 (298 K)	1	[18]
T(7.5)/U-azo	AB	Side group	1.8 (273K)	0.8 (273 K)	3	[19]
PCN-250	AB	backbone	3.8 (298 K)	2.2 (298 K)	5	[20]
PCN-123	AB	Side group	1.0 (295K)	0.5 (295 K)	2	[21]
$PCTF_2$	AB	Side group	1.94 (273K)	0.4 (273 K)	3	[22]
P-OMe-UiO	SP	Linker	2.4 (273K)<br>1.03 (298 K)	1.1 (273 K)<br>0.4 (298 K)	6	This work

We also evaluated the framework integrity of P-OMe-UiO after six cycles of photo-induced swing adsorption. FT-IR and PXRD characterisation confirms that framework integrity is preserved across all six cycles (Figure S29). After three cycles, the primary peak at 2θ = 6.6° broadens only modestly (4% FWHM increase) with all characteristic peaks fully retained and unshifted. After six cycles, a more pronounced drop in primary-peak intensity is observed, broadly matching the 35% reduction in working capacity at SP6. This is attributed to localised MC aggregation within pore channels reducing crystalline domain size.[23] Critically, all characteristic framework peaks remain present and unshifted at SP6, and FT-IR framework absorption bands are unchanged. This confirms that the primary framework connectivity and chemical integrity are fully preserved. The observed crystallinity reduction reflects cumulative UV-induced photobleaching of the SP chromophore. This progressively depletes photoswitchable SP units and promotes MC aggregation, rather than irreversible MOF decomposition.

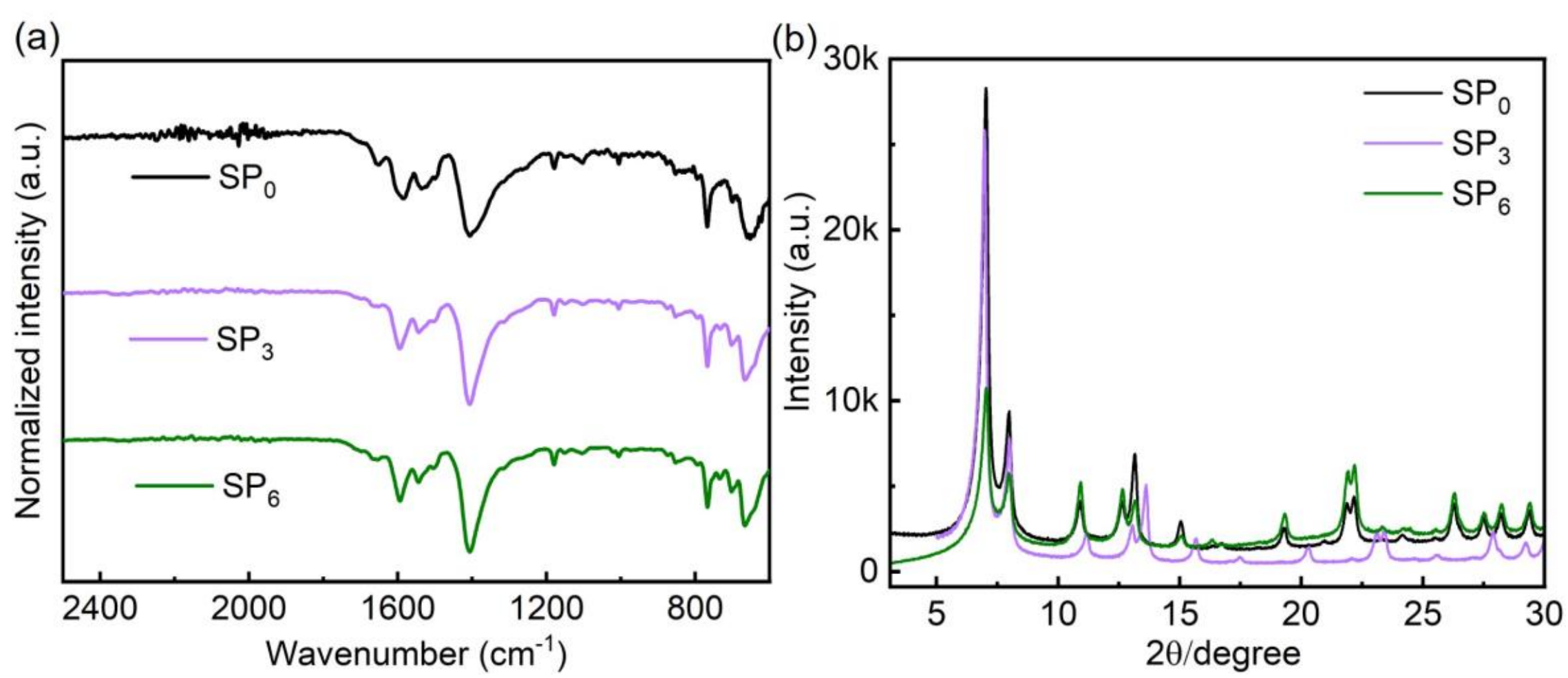

Figure S29. (updated in supporting information) (a) FTIR of P-OMe-UiO after 3 and 6 cyclic testing. (b) Powder X-ray diffraction (PXRD) patterns of P-OMe-UiO collected at different photo switching cycle stages.

The revised durability statement in the main text now reads:

Results and Discussion Page 14 Line 17: The photo-modulated CO_2 working capacity of P-OMe-UiO is sustained across six SP/MC switching cycles, retaining 88% of initial SP-state CO_2 uptake (0.74 mmol g^{-1} after 15 h cumulative UV irradiation). Post-cycling PXRD and FT-IR confirm full preservation of UiO-67 framework topology and chemical integrity, with all characteristic diffraction peaks and absorption bands unchanged. The progressive capacity reduction is attributed to UV-induced photobleaching of the SP chromophore and MC aggregation rather than framework degradation[23], with more pronounced decline beyond six cycles defining the current operational boundary. This cycling performance compares favourably with the majority of photo-responsive MOFs reported to date (Table S3), and could be further extended through incorporation of more photostable SP derivatives[5] or polymer-tethered SP systems[7] to suppress aggregation-induced degradation.

6) The authors have reported the working capacity and gas adsorption isotherms exclusively at 273 K. It is unclear why this specific temperature was chosen for a material intended for carbon capture applications.

Response: As shown in Table S3 (Part of our response to Reviewer 1's Comment #5 on Page 7 of this document), 273 K is the widely adopted benchmark for CO_2 adsorption in the MOF literature, enabling direct comparison with comparable materials. We have additionally measured CO_2 working capacity at 298 K for P-OMe-UiO (Figure S27a), yielding a photo-modulated working capacity of 0.40 mmol g^{-1}. This value serves as the basis for our lifecycle assessment (LCA), confirming the energetic and environmental benefits of light-driven CO_2 capture and regeneration. At 298 K, CO_2/N_2 IAST selectivity after UV irradiation remains ~ 98. This is consistent with breakthrough experiments using a 20:80 CO_2/N_2 mixture, where dark-state CO_2 uptake of 0.40 mmol g^{-1} falls to 0.32 mmol g^{-1} after UV irradiation. Together, these data establish the practical viability of P-OMe-UiO under flue gas separation conditions.

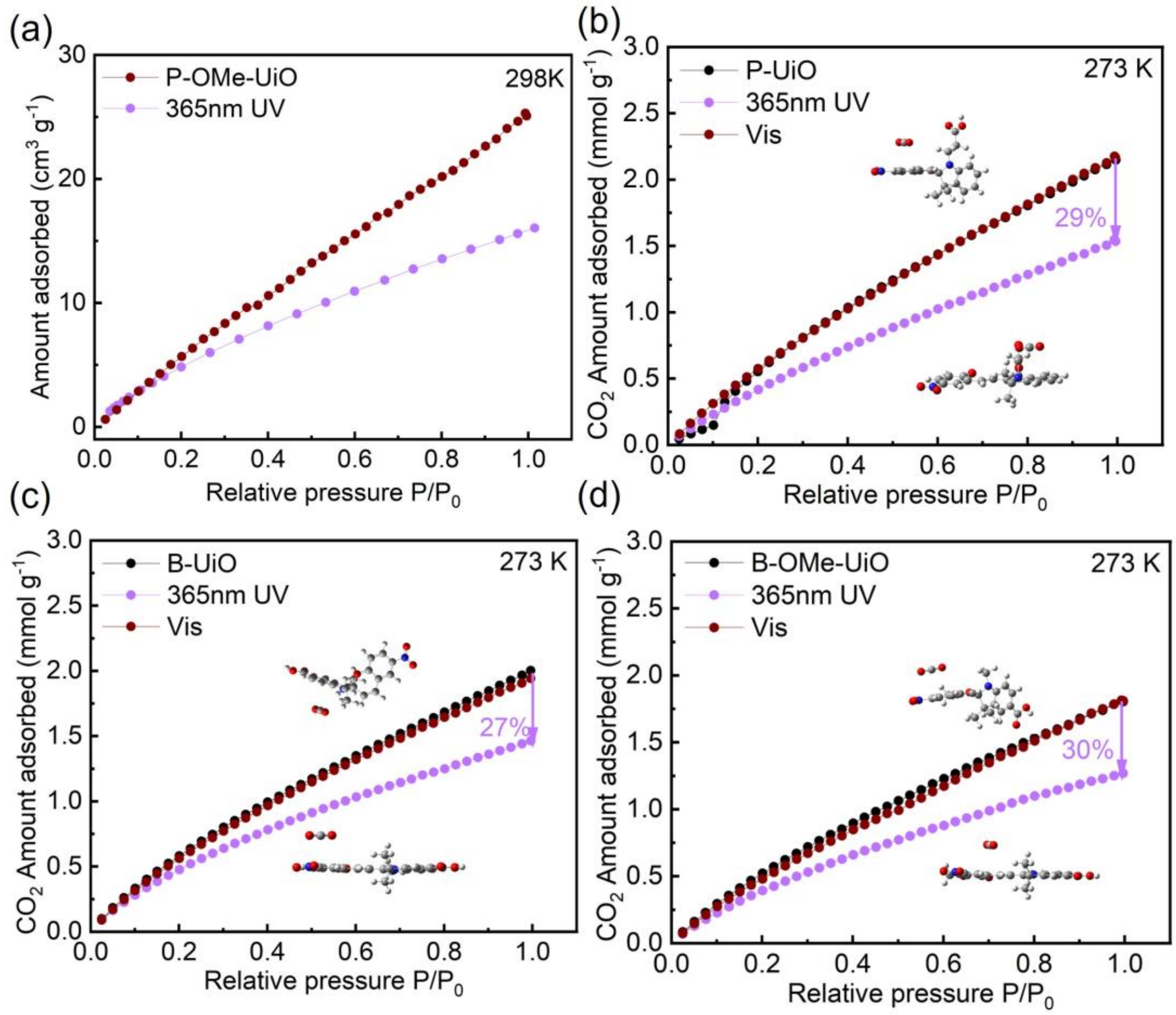

Figure S27. CO_2 adsorption isotherms comparing materials before (365 nm UV) and after (450 nm visible light) photo-irradiation of (a) P-OMe-UiO at 298 K, (b) P-UiO, (c) B-UiO and (d) B-OMe-UiO at 273 K. All materials show Type I behavior with reduced uptake in MC state.

The following text has been inserted into the main manuscript in Page 16:

Results and Discussion Page 16 Line 11: In the SP state, P-OMe-UiO achieves a CO_2/N_2 selectivity of 500, 51% higher than P-UiO, with negligible N_2 adsorption in both SP and MC states. UV irradiation reduces selectivity to ~98, reflecting selective modulation of CO_2 adsorption by MC-Zr complexation while N_2 rejection is preserved.

To evaluate performance under realistic conditions, breakthrough experiments were conducted using a 20:80 CO_2/N_2 mixture (Figure 5f, S31), building on the photo-modulated working capacity of 0.40 mmol g^{-1} and IAST selectivity of up to 500 measured at 298 K (Figure S26a).

Overall, the science disclosed in this manuscript is appropriate for the broad readership of Nature Communications. This work represents a clever application of coordination chemistry within a porous framework, likely to inspire further efforts to develop 'smart' adsorbents. However, prior to publication, the authors must address the significant concerns delineated above to ensure the mechanistic claims are as robust as the conceptual framework.

Response: Based on our revisions to the manuscript and SI above, we strongly believe that all comment and concerns of Reviewer 1 have been addressed robustly.

Reviewer 2

In this manuscript, the authors report a conceptually novel mechanism of photo-reversible defect complexation in which spiropyran (SP) photoswitches grafted onto open Zr sites in defective UiO-67 MOFs enabling reversible coordination of the MC phenolate oxygen with adjacent open Zr sites. This directly modulates active binding site availability rather than relying on conventional pore geometry or polarity changes. The resulting CO_2 working capacity of 1.1 mmol g^{-1} represents an 83% enhancement over non-defect-switchable analogues, and the generality of the mechanism is demonstrated across both gas-phase and solution-phase cargo. The work is supported by a comprehensive and well-integrated characterization method including ex-situ, in-situ, and NAP-XPS, DFT simulations, lifecycle assessment, and breakthrough experiments. The manuscript is well-written and the central hypothesis is clearly articulated. I consider this work to be a significant conceptual advance in the field of stimuli-responsive porous materials and recommend publication in Nature Communications subject to revision addressing the points below.

1. On page 12, the text states "P-UiO showed significantly lower Na^+ uptake of only 0.07 mmol g^{-1}" but the subject of the comparison should be P-OMe-UiO, not P-UiO. This appears to be a typographical error that inverts the intended comparison and should be corrected carefully as it affects the logical flow of the argument. There are also some other typing errors that need to be revised: page 18: "This apparent paradox is resolve by" should be "resolved". page 17 contains a typographical error ("pore volume loos" should be "pore volume loss")

Response: We sincerely thank the reviewer for their careful reading. All three corrections have been made in the revised manuscript:

Page 12 Line 20: In contrast, P-OMe-UiO showed significantly lower Na^+ uptake of only 0.07 mmol g^{-1} under identical conditions, consistent with MC-Zr complexation suppressing phenolate oxygen accessibility.

Page 18: The sentence containing this typographical error has been rewritten in full as part of our response to Reviewer 1's Comment #2.

Page 17 Line 8: This decoupling of pore volume loss from..

2. The lifecycle assessment (LCA) results are cited as supporting evidence for the low environmental impact of this approach, yet the LCA methodology, system boundaries, and key assumptions are relegated to the Supporting Information. Given that the LCA is presented as a key differentiator of this work, the authors should include at least a brief description of the LCA methodology and its key assumptions in the main text, alongside a statement of any limitations. This is particularly important given that the 60 J energy figure cited in the main text differs from the 240 J figure stated in the Table of Contents entry, which requires clarification.

Response: We thank the reviewer for this constructive suggestion. We have revised the manuscript accordingly. Specifically, a dedicated methodological paragraph has been added to page 16 of the revised manuscript as follows:

Page 16 Line 20: A cradle-to-gate LCA was conducted to evaluate and compare the environmental impacts associated with the operational energy consumption of LISA, temperature swing adsorption, and pressure swing adsorption CO_2 capture systems, and the functional unit defined as the amount of adsorbent required to capture 1 mol of CO_2. The system boundary was confined to the operational phase, encompassing energy consumption associated with adsorption-desorption cycling and adsorbent

regeneration. Key assumptions include constant adsorbent performance over repeated cycling and energy modelled using the UK electricity grid mix. Compared to traditional carbon capture processes like TSA and PSA, LCAs indicated that the environmental impact of carbon capture was significantly lower when light-induced swing adsorption enabled with P-OMe-UiO was used. This was due to the low energy demand of using P-OMe-UiO to create a CO_2 working capacity, requiring only 60 J per adsorption-desorption cycle (240 J cumulative over four cycles).

A full discussion of study limitations has been provided in the Supporting Information (Section 1.5.1) as follows:

The system boundary was limited to the operational phase, specifically the energy consumption associated with adsorption-desorption cycling and adsorbent regeneration, while upstream and downstream processes such as adsorbent synthesis, equipment fabrication, infrastructure, and end-of-life treatment were excluded. Key assumptions include:

1. Adsorbent materials (P-OMe-UiO and UiO-67) were assumed to maintain structural integrity and adsorption capacity during repeated adsorption-desorption cycles, with no loss of activity, material degradation, or replacement requirement considered.

2. All energy demands were modelled using the United Kingdom electricity grid mix available in the SimaPro database, assuming a constant grid composition throughout the assessment period.

3. Thermal regeneration in the TSA process was assumed to be supplied entirely through electric heating to maintain methodological consistency and comparability among the LISA, TSA, and PSA systems.

4. Ideal energy transfer was assumed during regeneration processes, where no additional energy losses from auxiliary equipment, heat dissipation, or system inefficiencies were considered unless explicitly reported in the source data.

5. Operational energy demand was assumed to be the dominant contributor within the defined system boundary.

Nevertheless, several limitations should be acknowledged. The present assessment focused solely on operational energy consumption and therefore did not capture potential environmental burdens associated with adsorbent synthesis, solvent usage, material replacement, or infrastructure requirements. Furthermore, the assumption of constant adsorbent performance neglected possible capacity decay and regeneration inefficiencies during long-term operation.

To avoid ambiguity, we have revised the Table of Contents caption which now reads:

Page 26: Photo-reversible complexation of merocyanine with open metal sites in defective UiO-67 MOF can deliver a light-induced CO_2 capture process that consumes only 60 J per adsorption-desorption cycle (240 J cumulative over four cycles).

3. The caption for Figure 4 does not match the corresponding image. Please either insert the correct figure related to metal adsorption experiments or modify the caption accordingly.

Response: We sincerely thank the reviewer for identifying this error. We have now supplied the correct figure in the revised manuscript.

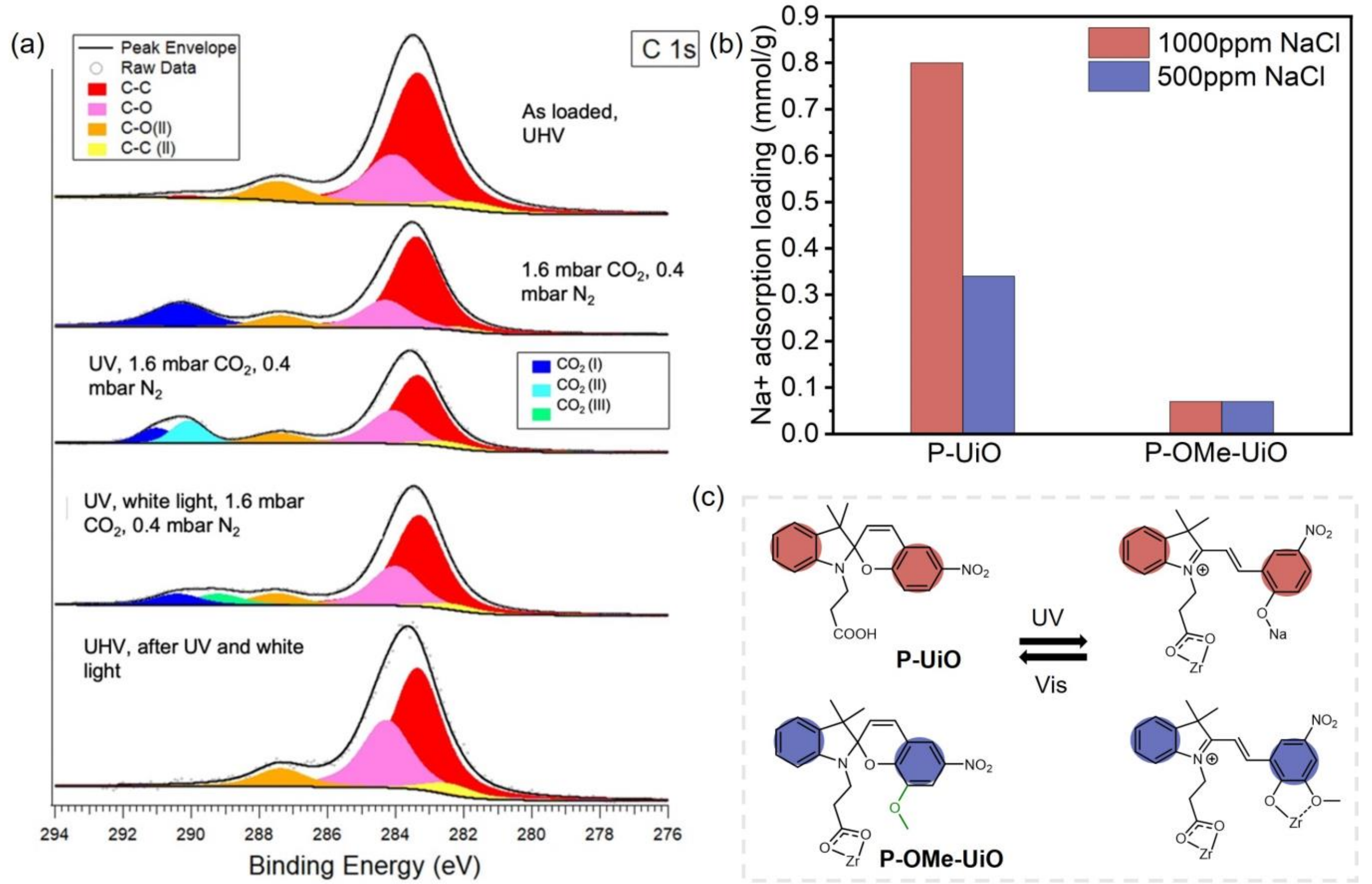

Figure 4. Evidence for defect complexation mechanism. (a) NAP-XPS C 1s spectra tracking C 1s binding energies of CO_2 under operando conditions: (i) P-OMe-UiO loaded in ultra-high vacuum. (ii) Exposing P-OMe-UiO to 1.6 mbar CO_2/0.4 mbar N_2 atmosphere. This mimicked CO_2 adsorption in darkness. (iii) UV irradiation of CO_2-loaded P-OMe-UiO in an atmosphere comprising 1.6 mbar CO_2/0.4 mbar N_2 gases. This mimicked using UV-irradiation to lower CO_2 adsorption and create a CO_2 working capacity. (iv) Vis-irradiation of CO_2-loaded P-OMe-UiO under 1.6 mbar CO_2/0.4 mbar N_2. This mimicked regeneration of P-OMe-UiO. (v) After evacuation (UHV). UV-irradiation released CO_2 adsorption as evidenced by increased peak intensity and complexity. (b) Na^+ adsorption capacities (mmol g^{-1}) of P-UiO and P-OMe-UiO in aqueous NaCl solutions with concentrations of 500 ppm and 1000 ppm. (c) Schematic illustration of the photoresponsive behavior of P-UiO and P-OMe-UiO under UV and visible light.

4. The authors present impressive CO_2/N_2 selectivity data (IAST = 500) for P-OMe-UiO under dark conditions in Figure 5e, which is a key strength of this material. However, a critical aspect requires further investigation. The proposed photo-reversible defect complexation mechanism demonstrates that UV irradiation substantially reduces the binding affinity of open Zr sites for CO_2, which would logically result in diminished CO_2/N_2 selectivity under illumination. To fully validate the practical viability of this photoswitchable approach, the authors should provide supplementary discussion on the IAST mixed-gas selectivity after SP to MC isomerization and explain whether this reduced selectivity would affect the purity in practical gas separation, for instance, whether significant amounts of N_2 would be co-desorbed during the desorption phase.

Response: We thank the reviewer for this insightful question. To directly evaluate the effect of SP/MC isomerization on selectivity, we measured CO_2 and N_2 isotherms of UV-irradiated P-OMe-UiO at 298 K (Figure S30). Upon UV irradiation, CO_2 uptake is substantially reduced while N_2 uptake remains negligibly low and effectively unchanged. This reflects that N_2 has no significant affinity for the open Zr

sites modulated by photo switching. Using these single-component isotherms for both states, we calculated the IAST CO_2/N_2 selectivity for a 20:80 mixture at 298 K (Figure S31): UV irradiation lowers the selectivity from 500 to 98. Although this is a marked decrease, a retained selectivity of 98 remains highly favourable for CO_2/N_2 separation and is consistent with our breakthrough tests. Therefore, no significant N_2 co-adsorption or co-desorption occurs in either phase.

The following text has been added to the main manuscript after the existing selectivity discussion (Page 16):

In the SP state, P-OMe-UiO achieves a CO_2/N_2 selectivity of 500, 51% higher than P-UiO, with negligible N_2 adsorption in both SP and MC states. UV irradiation reduces selectivity to ~98, reflecting selective modulation of CO_2 adsorption by MC-Zr complexation while N_2 rejection is preserved.

To evaluate performance under realistic conditions, breakthrough experiments were conducted using a 20:80 CO_2/N_2 mixture (Figure 5f, S31), building on the photo-modulated working capacity of 0.40 mmol g^{-1} (Figure S26a) and IAST selectivity of up to 500 measured at 298 K (Figure S30c).

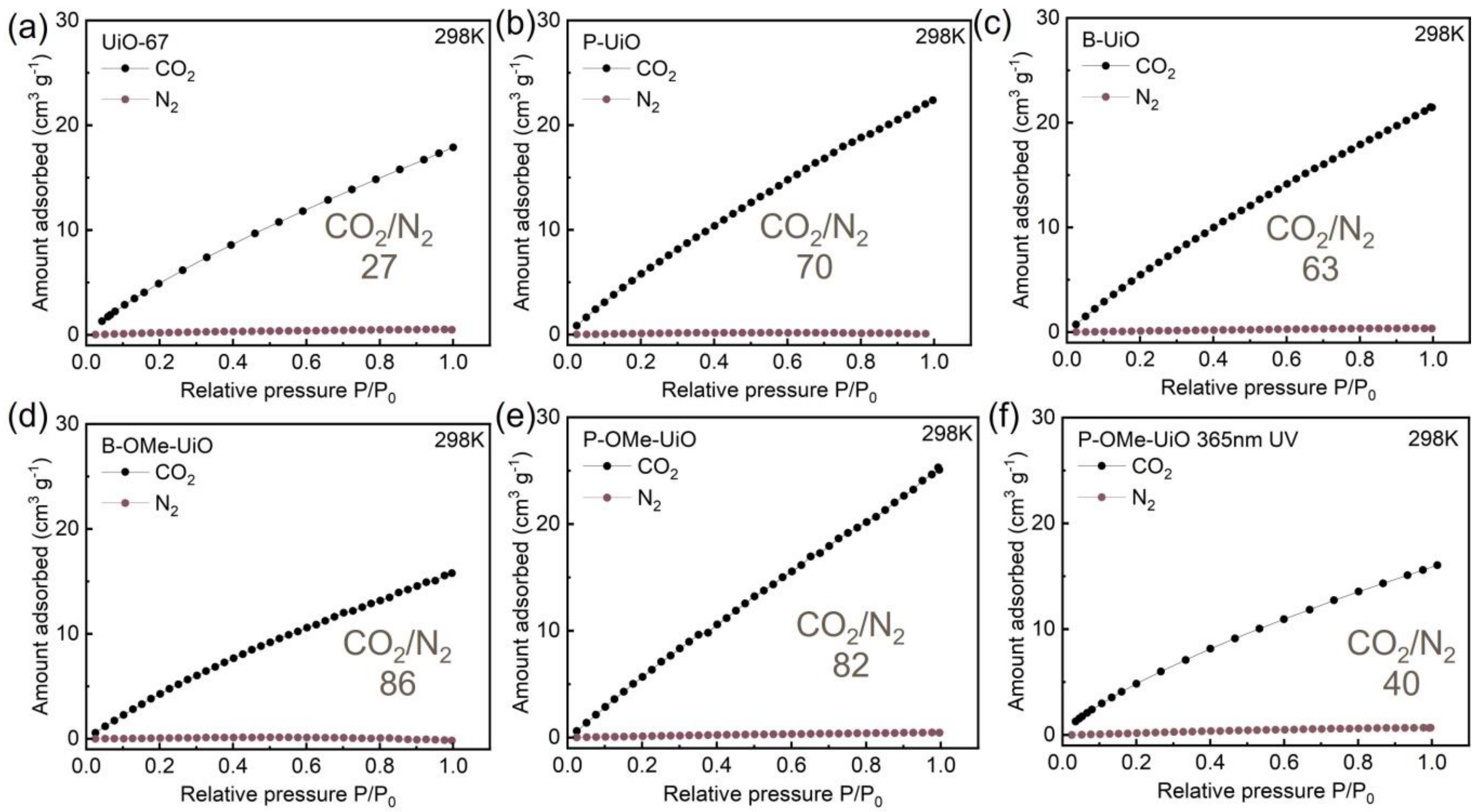

Figure S30. The N_2 and CO_2 adsorption isotherms of (a) parent UiO-67; (b) P-UiO; (c) B-UiO; (d) B-OMe-UiO; (e) P-OMe-UiO and (f) P-OMe-UiO after UV at 298 K. The CO_2/N_2 selectivity of all SP-UiO samples was investigated by testing the N_2 and CO_2 adsorption isotherms. As shown in Figure, all SP-UiO samples exhibited markedly lower N_2 uptake, indicative of a preferential binding affinity for CO_2 molecules and thus a high intrinsic CO_2/N_2 selectivity. The CO_2/N_2 selectivity values were investigated by Henry's constants under low-pressure conditions. The parent UiO-67 showed lowest CO_2/N_2 selectivity. Incorporation of various SP moieties not only enhanced the CO_2 uptake capacity but also significantly improved CO_2/N_2 selectivity. Notably, the CO_2/N_2 selectivity P-OMe-UiO increased by 200% compared to parent UiO-67.

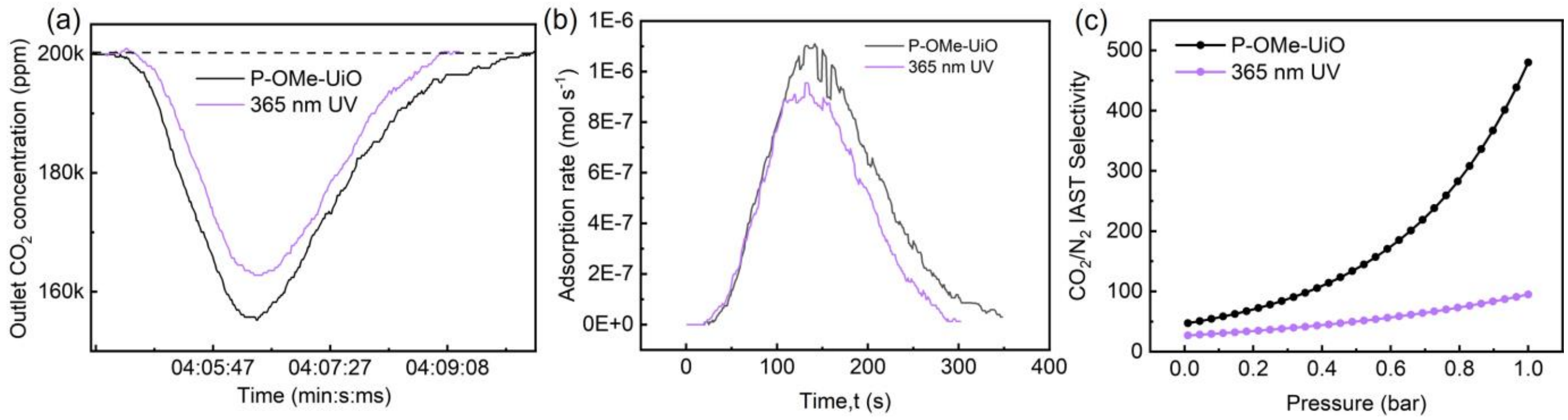

Figure S31. (a) Breakthrough experiments of P-OMe-UiO using a simulated flue gas mixture containing 20 vol% CO_2 in N_2. The gas stream was passed through a quartz tube packed with 400 mg of P-OMe-UiO, and the outlet CO_2 concentration was monitored over time. (b) CO_2 adsorption rate of P-OMe-UiO using a simulated flue gas mixture containing 20 vol% CO_2 in N_2. (c) IAST CO_2/N_2 selectivity of P-OMe-UiO after UV calculated using 20:80 CO_2 and N_2 mixtures at 298 K.

5. The manuscript reports CO_2 adsorption enthalpy decreasing from 45 to 16 kJ mol^{-1} upon UV irradiation, which is crucial evidence for the LISA mechanism. However, the calculation methodology for these isosteric heat (Q_{st}) values requires clarification. The authors should specify in the Supporting Information or methods section: the calculation approach used (e.g., Clausius-Clapeyron analysis), temperature conditions. This documentation is essential for reproducibility and validation of these key energetic parameters.

We thank the reviewer for this important comment regarding methodological transparency. We confirm that the isosteric enthalpy of adsorption (Q_{st}) values reported in the manuscript were calculated using the Clausius-Clapeyron analysis, based on CO_2 adsorption isotherms measured at two temperatures (273 K and 298 K). The Clausius-Clapeyron equation was applied as follows:

$$Q_{st}=-R\frac{\partial lnP}{\partial(1/T)}\Big|_n$$

where R is the universal gas constant, P is the equilibrium pressure, T is the absolute temperature, and n is the adsorbed amount held constant across isotherms.

Thus, a methodology paragraph to ensure that the calculation methodology, temperature conditions, and fitting procedure are fully documented and reproducible has been added to the Supporting Information (section 1.3.10) as follows:

The isosteric enthalpy of adsorption (Q_{st}) for CO_2 was determined using the Clausius-Clapeyron analysis. CO_2 adsorption isotherms were collected at 273 K and 298 K for all SP-UiO samples, both prior to and following UV irradiation. At each loading point, the isosteric heat was calculated according to the Clausius-Clapeyron equation:

$$Q_{st}=-R\frac{\partial lnP}{\partial(1/T)}\Big|_n$$

where R is the universal gas constant (8.314 J mol^{-1} K^{-1}), P is the equilibrium pressure at a given adsorbed amount n, and T is the absolute temperature. Adsorption isotherms were fitted using a dual-site Langmuir model prior to Q_{st} calculation to enable interpolation at consistent loading points across both temperatures.

6. The authors report IAST selectivity at 298 K but do not provide the CO_2 working capacity of the lead material P-OMe-UiO at this temperature. Since working capacity is the central performance metric of this study, it is recommended that the authors include the photo-modulated CO_2 working capacity of P-OMe-UiO at 298 K for a brief comparison, which would substantially enhance the demonstration of its practical application potential.

We thank the reviewer for this constructive suggestion. Please refer to our response to Reviewer 1's Point 6. P-OMe-UiO yields a photo-modulated CO_2 working capacity of 0.4 mmol g^{-1} at 298 K. We have also revised the main manuscript accordingly.

Reference

1. Yuan S, *et al.* Sequential linker installation: precise placement of functional groups in multivariate metal-organic frameworks. *J Am Chem Soc* **137**, 3177–3180 (2015).
2. Shearer GC, Vitillo JG, Bordiga S, Svelle S, Olsbye U, Lillerud KP. Functionalizing the Defects: Postsynthetic Ligand Exchange in the Metal Organic Framework UiO-66. *Chemistry of Materials* **28**, 7190–7193 (2016).
3. Yuan S, *et al.* Linker Installation: Engineering Pore Environment with Precisely Placed Functionalities in Zirconium MOFs. *J Am Chem Soc* **138**, 8912–8919 (2016).
4. Astley S, *et al.* Identifying chemical and physical changes in wide-gap semiconductors using real-time and near ambient-pressure XPS. *Faraday Discuss* **236**, 191–204 (2022).
5. Cui L, *et al.* Substituent effect on the acid-induced isomerization of spiropyran compounds. *Spectrochim Acta A Mol Biomol Spectrosc* **202**, 13–17 (2018).
6. Das G, *et al.* Light-driven self-assembly of spiropyran-functionalized covalent organic framework. *Nat Commun* **14**, 3765 (2023).
7. Ou R, *et al.* A sunlight-responsive metal–organic framework system for sustainable water desalination. *Nature Sustainability* **3**, 1052–1058 (2020).
8. Castellanos S, *et al.* Structural Effects in Visible-Light-Responsive Metal-Organic Frameworks Incorporating ortho-Fluoroazobenzenes. *Chemistry* **22**, 746–752 (2016).
9. Song WC, Cui XZ, Liu ZY, Yang EC, Zhao XJ. Light-triggered Supramolecular Isomerism in a Self-catenated Zn(II)-organic Framework: Dynamic Photo-switching CO_2 Uptake and Detection of Nitroaromatics. *Sci Rep* **6**, 34870 (2016).
10. Huang H, Sato H, Aida T. Crystalline Nanochannels with Pendant Azobenzene Groups: Steric or Polar Effects on Gas Adsorption and Diffusion? *J Am Chem Soc* **139**, 8784–8787 (2017).
11. Zheng Y, Sato H, Wu P, Jeon HJ, Matsuda R, Kitagawa S. Flexible interlocked porous frameworks allow quantitative photoisomerization in a crystalline solid. *Nat Commun* **8**, 100 (2017).
12. Dang L-L, Zhang X-J, Zhang L, Li J-Q, Luo F, Feng X-F. Photo-responsive azo MOF exhibiting high selectivity for CO2and xylene isomers. *J Coord Chem* **69**, 1179–1187 (2016).
13. Fan CB, *et al.* Photoswitching adsorption selectivity in a diarylethene-azobenzene MOF. *Chem Commun (Camb)* **53**, 763–766 (2017).
14. Prasetya N, Ladewig BP. Dynamic photo-switching in light-responsive JUC-62 for CO_2 capture. *Sci Rep* **7**, 13355 (2017).
15. Prasetya N, Donose BC, Ladewig BP. A new and highly robust light-responsive Azo-UiO-66 for highly selective and low energy post-combustion CO_2 capture and its application in a mixed matrix membrane for CO_2/N_2 separation. *Journal of Materials Chemistry A* **6**, 16390–16402 (2018).
16. Prasetya N, Ladewig BP. New Azo-DMOF-1 MOF as a Photoresponsive Low-Energy CO_2 Adsorbent and Its Exceptional CO_2/N_2 Separation Performance in Mixed Matrix Membranes. *ACS Appl Mater Interfaces* **10**, 34291–34301 (2018).
17. Lyndon R, Konstas K, Ladewig BP, Southon PD, Kepert PC, Hill MR. Dynamic photo-switching in metal-organic frameworks as a route to low-energy carbon dioxide capture and release. *Angew Chem Int Ed Engl* **52**, 3695–3698 (2013).
18. Gong le L, Feng XF, Luo F. Novel azo-Metal-Organic Framework Showing a 10-Connected bct Net, Breathing Behavior, and Unique Photoswitching Behavior toward CO_2. *Inorg Chem* **54**, 11587–11589 (2015).
19. Jiang Y, *et al.* Metal-Organic Frameworks with Target-Specific Active Sites Switched by Photoresponsive Motifs: Efficient Adsorbents for Tailorable CO_2 Capture. *Angew Chem Int Ed Engl* **58**, 6600–6604 (2019).
20. Li H, *et al.* A Robust Metal-Organic Framework for Dynamic Light-Induced Swing Adsorption of Carbon Dioxide. *Chemistry* **22**, 11176–11179 (2016).
21. Park J, Yuan D, Pham KT, Li JR, Yakovenko A, Zhou HC. Reversible alteration of CO_2 adsorption upon photochemical or thermal treatment in a metal-organic framework. *J Am Chem Soc* **134**, 99–102 (2012).
22. Huang Q, Zhan Z, Sun R, Mu J, Tan B, Wu C. Light Triggered Pore Size Tuning in Photoswitching Covalent Triazine Frameworks for Low Energy CO(2) Capture. *Angew Chem*

Int Ed Engl **62**, e202305500 (2023).

23. Scandura G*, et al.* Photo-responsive metal–organic frameworks – design strategies and emerging applications in photocatalysis and adsorption. *Materials Advances* **4**, 1258–1285 (2023).
